# Synthesis and Biological Evaluation of Novel Synthetic Indolone Derivatives as Anti-Tumor Agents Targeting p53-MDM2 and p53-MDMX

**DOI:** 10.3390/molecules27123721

**Published:** 2022-06-09

**Authors:** Yali Wang, Bo Ji, Zhongshui Cheng, Lianghui Zhang, Yingying Cheng, Yingying Li, Jin Ren, Wenbo Liu, Yuanyuan Ma

**Affiliations:** 1 School of Pharmacy and Life Sciences, Jiujiang University, Jiujiang 332000, China; xhawx233@163.com (B.J.); csz13479842156@163.com (Z.C.); jjbio@126.com (L.Z.); m15807983494@163.com (Y.C.); 19142049457@163.com (Y.L.); yanjiushengrj@126.com (J.R.); bowenliu2008@163.com (W.L.); 2 Institute of Medicinal Biotechnology, Chinese Academy of Medical Science and Peking Union Medical College, Beijing 100050, China; mayuanyuancircle@163.com

**Keywords:** p53-MDM2, p53-MDMX, indolone derivatives, anti-tumor

## Abstract

A series of novel indolone derivatives were synthesized and evaluated for their binding affinities toward MDM2 and MDMX. Some compounds showed potent MDM2 and moderate MDMX activities. Among them, compound A13 exhibited the most potent affinity toward MDM2 and MDMX, with a *K_i_* of 0.031 and 7.24 μM, respectively. A13 was also the most potent agent against HCT116, MCF7, and A549, with IC_50_ values of 6.17, 11.21, and 12.49 μM, respectively. Western blot analysis confirmed that A13 upregulated the expression of MDM2, MDMX, and p53 by Western blot analysis. These results indicate that A13 is a potent dual p53-MDM2 and p53-MDMX inhibitor and deserves further investigation.

## 1. Introduction

Tumor suppressor p53 plays a crucial role in the regulation of DNA repair, cell cycle, apoptosis, and differentiation [1,2]. It is inactivated in the majority of human cancers, resulting from mutations or the overexpression of its negative regulators, murine double minute 2 (MDM2) and/or murine double minute X (MDMX) [3]. Approximately 50% of human cancers have a p53 gene mutation and may be combated using gene therapy. For the remaining cancer types, the function of p53 can be reactivated by disrupting the interaction with MDM2 and MDMX proteins using small-molecule inhibitors [4]. Therefore, the interfaces of p53-MDM2 and p53-MDMX have emerged as important targets for the development of novel chemotherapeutic agents.

**Figure 1:**
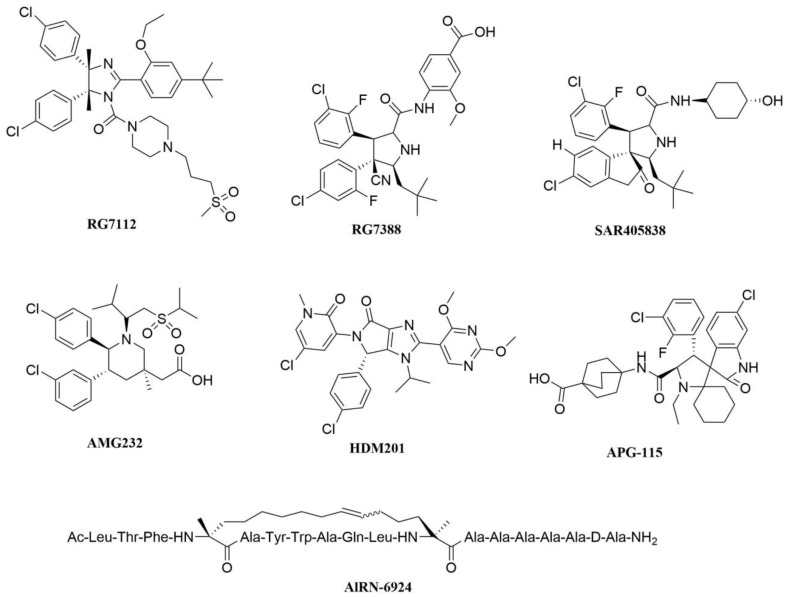
Representative structures of the p53-MDM2 and p53-MDM2/MDMX inhibitors entering clinical studies.

To date, over 20 chemotypes have been reported as p53-MDM2 inhibitors including nutlins [5], spiro-oxindole [6], benzodiazepinedione [7], isoquinolin-1-one [8], chromenotriazolopyrimidines [9], imidazolyl indole [10], piperidine [11], chalcone [12], diketopiperazines [13], morpholinone [14], pyrrolin-2-one [15], and many others. Several p53-MDM2 inhibitors such as RG7112 [16], RG7388 [17], SAR405838 [18], AMG-232 [19], HDM201 [20], and APG-115 [21] (Figure 1) have progressed into clinical trials. However, p53-MDM2 inhibitors are inadequate when MDMX is also expressed at a high level in some cancer cells. A few p53-MDMX inhibitors have also been reported such as SJ-172550 [22], NSC207895 [23], and NSC146109 [24].

Studies have verified that dual MDM2/MDMX inhibitors can activate p53 better than selective inhibitors that only target MDM2 or MDMX [25]. Currently, some progress has been made in designing dual inhibitors of MDM2 and MDMX [26,27,28,29,30]. ALRN-6924 (Figure 1), a dual inhibitor of MDM2 and MDMX, has gone into a phase I clinical trial (NCT04022876) in patients with solid tumors [31]. However, there are many challenges that need to be overcome before use in clinical practice and novel inhibitors need to be developed.

Indole, as a heterocyclic molecule, is a significant source of the pharmacologically active compound. Due to its chemical reactivity, indole could be modified to obtain many indole derivatives, exhibiting a variety of bioactivities. Especially as anti-cancer agents, indoles are effective against a variety of cancers including acute myeloid leukemia [32,33], lung cancer [34], cervical cancer [35], colon cancer, breast cancer [36], and others. The targets of indoles as anti-cancer agents were also reported such as the Mcl-1, Pim, HDAC, SIRT, tubulin [32,37], RAF-MEK-ERK, and PI3K-PDK1-AKT pathways [34], and so on. Some indole derivatives have even been identified as very promising candidates for phototherapy application against highly aggressive and resistant cancers [38]. 

**Figure 2:**
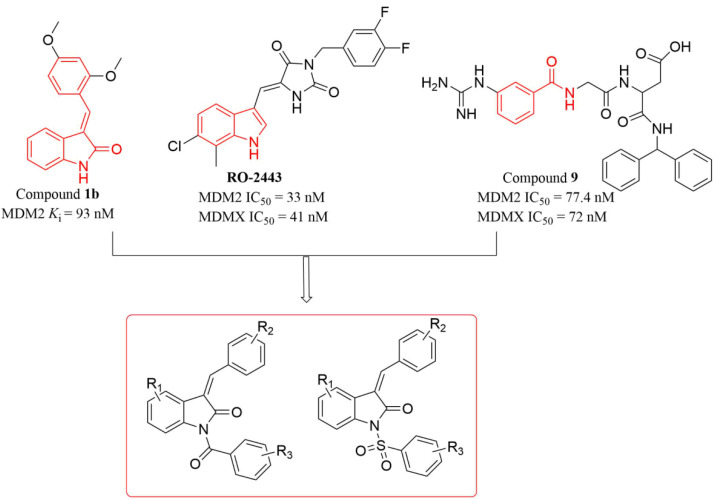
The design strategy of the target compounds.

As shown in Figure 2, some indole derivatives, which are reported as anti-cancer agents targeting MDM2 and MDMX (1b, RO-2443) [36,39], have been modified to improve the activities and explore new mechanisms. In the present study, novel indolone compounds were identified and the derivatives were synthesized and evaluated for their affinities toward MDM2 and MDMX. The most active compounds were tested for their in vitro anti-tumor activity against the MCF7, HCT116, and A549 cell lines to verify their antiproliferative activity.

## 2. Results and Discussion

### 2.1. Chemistry

**Scheme 1:**
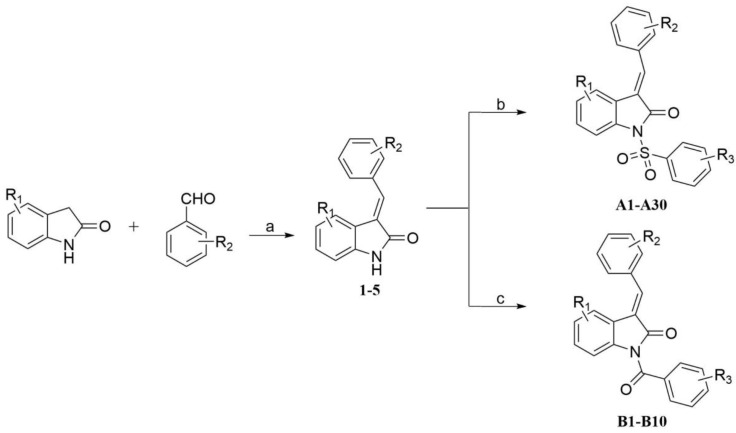
The synthetic route for target compounds **A1**–**A30** and **B1**–**B10**. Reagents and conditions: (**a**) EtOH, NaOH, 60 °C, 4 h; (**b**) Substituted benzenesulfonyl chloride, NaOH, THF, 0~5 °C, 1~1.5 h; (**c**) Substituted benzoyl chloride, NaOH, THF, 0~5 °C, 1~1.5 h.

Compounds **A1–A30** and **B1–B10** were synthesized according to Scheme 1. A condensation reaction was carried out to obtain intermediate compounds **1–5** with the commercially available substituted oxindole and benzaldehyde materials. Next, compounds **1–5** were sulfonylated or acylated to obtain **A1–A30** and **B1–B10**, with corresponding benzene sulfonyl chloride or benzoyl chloride in the presence of sodium hydroxide in tetrahydrofuran. In total, 40 derivatives were synthesized and the compound structures are listed in Table 1. The ^1^H NMR spectrum, ^13^C NMR spectrum, and mass spectrum of compounds **1**–**5**, **A1**–**A30**, and **B1**–**B10** were listed in the Supplementary Materials.

### 2.2. In Vitro Enzymatic Assays and Structure–Activity Relationships

The MDM2 and MDMX binding evaluation for the target compounds was carried out using the fluorescence polarization assay. The bioactivity data are summarized in Table 1, where nutlin-3a was used as the positive control. The assay was validated by nutlin-3a with a *K_i_* value of 0.15 μM, which is comparable to the previously reported value [40]. In general, all of the synthes0-0-0- ized compounds (**A1**–**A30** and **B1**–**B10**) showed a moderate to high binding affinity to MDM2. Most of the **A1**–**A30** compounds showed a moderate binding affinity to MDMX. None of the **B1**–**B10** compounds showed MDMX activities. 

Compound **A1** (R_1_ = H, R_2_ = 2-Cl, R_3_ = H) demonstrated a moderate binding affinity to MDM2 (*K_i_* = 4.39 μM). The introduction of methyl on sulfonyl moiety (**A2**–**A4**, R_3_ = 2-CH_3_, 3-CH_3_, 4-CH_3_) led to a lower potency compared to that of **A1**. However, compounds **A5**–**A7** with a chlorine group (R_3_ = 2-Cl, 3-Cl, 4-Cl) showed greater activity than **A1**. Compared to **A1**, when the R_2_ 2-Cl group was replaced with 3-Cl, **A8** exhibited a potency that was three-fold higher. Surprisingly, the introduction of a chlorine group to R_3_ of **A8** (**A12**–**A14**, R_3_ = 2-Cl, 3-Cl, 4-Cl) contributed to an obvious enhancement in activity, with a *K_i_* of 0.19, 0.031 and 0.082 μM, respectively. **A13** and **A14** especially showed a greater potency than nutlin-3a. However, the results were not satisfactory when the methyl group was introduced to the same position (**A9**–**A11**). This phenomenon indicates that an electron-withdrawing group might be needed to increase the enzyme affinity. Other electron-withdrawing groups were introduced in subsequent experiments including the bromine, fluorine, trifluoromethyl, and nitro groups. Compounds **A16**, **A19**, and **A21** with the 3-substituted electron-withdrawing R_3_ group exhibited more potency than the compound with 2-substituted or 4-substituted group (**A15**, **A17**, **A18**, and **A20**). **A16**, **A19**, and **A21** exhibited an even higher potency than nutlin-3a. Compared to **A1** and **A8**, when the 2-Cl or 3-Cl group of R_2_ was replaced with 4-Cl, **A28** exhibited a lower activity than **A8**, but greater than **A1**. Next, structure modification was carried out at the position of R_3_ (**A23**–**A27)** with *K_i_* values of >1 μM. **A29** and **A30** showed a lower activity than **A13** with the introduction of the chlorine atom to R_1_ (5-Cl and 6-Cl). Among the **B1**–**B10** compounds with benzoyl groups, **B3** showed the most potency with a *K_i_* of 7.86 μM, which was lower than the A series with benzene sulfonyl groups. 

Potency analysis of the compounds toward MDMX was carried out in the following assays. **A13** was the most potent MDMX binder with a *K_i_* of 7.24 μM. The *K_i_* values of some compounds (**A5–A8**, **A12–A24**, and **A27–A30**) ranged from 10.33 to 43.18 μM, while the rest of the compound values were >50 μM. Despite the significant homology of MDM2 and MDMX, the affinities of the same compound were notably different.

In general, the results listed in Table 1 showed that the activity of compounds **A1–A30** was better than that of compounds **B1–B10**, demonstrating that the introduction of the benzene sulfonyl chloride group led to a greater potency than the benzoyl chloride group. Compound **A13** showed the most potent affinity toward MDM2 and MDMX, with a *K_i_* of 0.031 and 7.24 μM, respectively.

### 2.3. In Vitro Anti-Tumor Activities

The anti-tumor activities of the most active compounds with *K_i_* < 1 μM toward MDM2 in the fluorescence polarization assay were evaluated against the MCF7, HCT116, and A549 cell lines in vitro using the standard MTT assay, with nutlin-3a as a positive control.

As illustrated in Table 2, all compounds showed anti-tumor activities. Except for **A15**, **A16**, and **A17**, all of the other tested compounds showed a greater potency against HCT116 than nutlin-3a. **A13**, in particular, exhibited satisfactory potency, with an IC_50_ of 6.17 μM, which was about three-fold greater compared to that of nutlin-3a. The IC_50_ values of the tested compounds against MCF7 ranged from 11.21 to 37.70 μM. Twelve compounds showed a greater activity than nutlin-3a. **A13** and **A21** were the most potent agents against A549, with IC_50_ values of 12.49 and 13.48 μM, respectively, which were superior to the positive control.

In general, all of the tested compounds showed a better potency against HCT116 than MCF7 and A549. **A13** was the most potent compound against HCT116, MCF7, and A549, with IC_50_ values of 6.17, 11.21, and 12.49 μM, respectively. Surprisingly, **A13** exhibited the best enzymatic affinities as well as anti-tumor activities. Further research on **A13** was carried out in the following experiment.

### 2.4. Western Blotting Analyses

**Figure 3:**
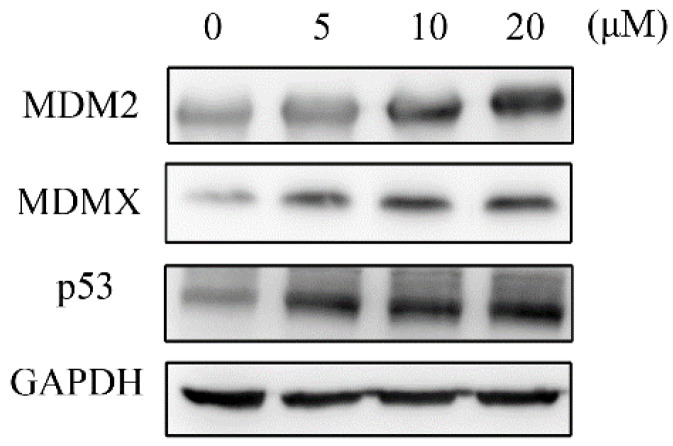
The Western blot analysis showing the expression of MDM2, MDMX, and p53 in the HCT116 cells treated with compound A13. GAPDH was included as a loading control.

The dual inhibition of the p53-MDM2 and p53-MDMX interactions is expected to activate p53, increasing its level in the cells with wild-type p53. In addition, p53 activation can also result in an increase in the levels of the MDM2 and MDMX proteins. To verify these predictions, Western blotting was used to assess the effects of compound **A13** in the HCT 116 cells. The cells were treated with compound **A13** for 48 h. Subsequently, the cells were lysed and protein extracts were analyzed for the levels of p53, MDM2, and MDMX. As shown in Figure 3, compound **A13** caused a dose-dependent increase in the level of p53 proteins in HCT116 cells, indicating a good activation of p53. Expression of MDM2 and MDMX as well as the p53 downstream gene was also induced in a dose-dependent manner, which is consistent with the prediction. According to these results, it can be concluded that compound **A13** might exhibit anti-cancer activity through interrupting the p53-MDM2 and p53-MDMX interactions.

### 2.5. Molecular Docking Analyses

**Figure 4:**
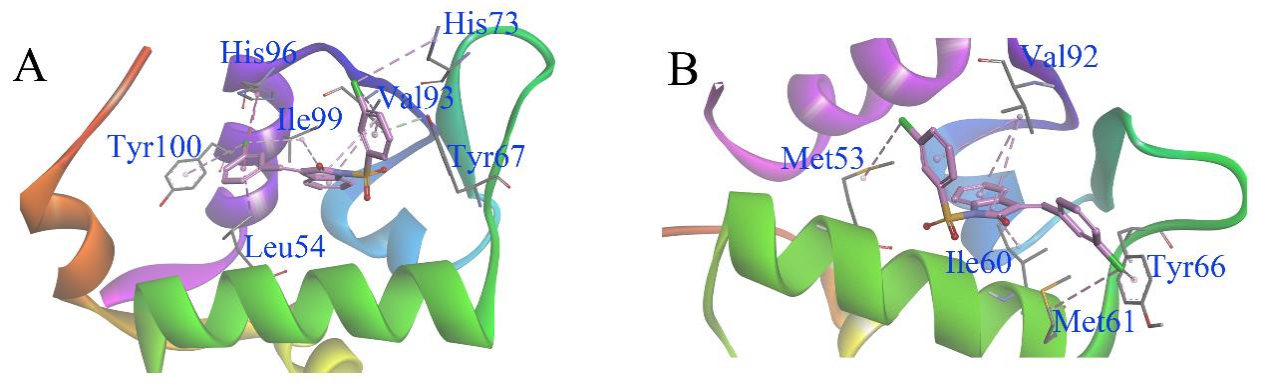
(**A**) The binding mode of compound **A13** in the active site of MDM2. (**B**) The binding mode of compound **A13** in the active site of MDMX. Compound **A13** is represented as stick diagrams with carbon atoms shown in pink.

Molecular docking was carried out and analyzed in order to observe the binding modes of compound **A13** in the active site of MDM2 and MDMX. The binding mode of compound **A13** in the MDM2 active site is shown in Figure 4A. The phenyl ring connected with the sulfonyl formed a non-classical H-bond with Tyr67, resulting in a hydrophobic interaction with Val93. The chlorine atom had σ–π and σ–σ hydrophobic interactions with His73 and Val93, respectively. The benzimidazole group formed a σ–π hydrophobic interaction with Vai93 and σ–σ hydrophobic interactions with Val93 and Ile99. The phenyl connected with a double bond formed a σ–π hydrophobic interaction with His96, and the chlorine atom made σ–π, σ–σ, and σ–π hydrophobic interactions with His96, Ile99, and Tyr100, respectively. The binding mode of compound **A13** in the MDMX active site is shown in Figure 4B. The chlorobenzene group connected with sulfonyl formed σ–π and σ–σ hydrophobic interactions with Val92 and Met53. The benzimidazole group made σ–π hydrophobic interactions with Val92 and Ile60. The chlorobenzene connected with a double bond made σ–σ and σ–π hydrophobic interactions with Met61 and Tyr66, respectively. The predicted *K_i_* values of compound **A13** binding to MDM2 and MDMX were 0.018 and 5.18 μM, consistent with the test values.

From the docking results exhibited in Figure 4, the interactions between compound **A13** and MDM2 were obviously stronger than those of **A13** and MDMX, which might explain the different enzymatic activities of **A13** targeting MDM2 and MDMX.

## 3. Experimental Section

### 3.1. General Procedures

The mass spectra were acquired using an Agilent 6210 ESI/TOF or ThermoFisher Q Exactive mass spectrometer. ^1^H and ^13^C NMR spectra were recorded on a Bruker BioSpin GmbH spectrometer at 400 or 600 MHz. Column chromatography was performed on silica gel (200–300 mesh) from Qingdao Ocean Chemical. Unless specified, all reagents were obtained from commercially available sources.

### 3.2. General Procedure for Preparation of Intermediates (**1**–**5**)

A mixture of indolone (7.51 mmol), 2-chlorobenzaldehyde (7.51 mmol), and sodium hydroxide (7.51 mmol) in ethanol (30 mL) was stirred at 60 °C for 4 h. Then, the reaction mixture was allowed to cool to room temperature and added to the ice water. The precipitate was collected by filtration and purified using recrystallization with methanol to obtain the intermediates **1**–**5**.

(Z)-3-(2-chlorobenzylidene)indolin-2-one (**1**): Yellow solid; yield: 85.2%; mp: 148.7–150.3 °C; ^1^H NMR (400 MHz, DMSO-*d_6_*): *δ* 7.77–7.74 (m, 1H), 7.63 (dd, *J* = 7.8, 1.9 Hz, 1H), 7.57 (s, 1H), 7.52–7.47 (m, 2H), 7.22 (td, *J* = 7.7, 1.0 Hz, 1H), 7.12 (d, *J* = 7.6 Hz, 1H), 6.86 (d, *J* = 7.7 Hz, 1H), 6.79 (td, *J* = 7.6, 0.9 Hz, 1H); ^13^C NMR (101 MHz, DMSO-*d_6_*): *δ* 167.90, 143.06, 132.66, 131.19, 131.03, 130.42, 130.01, 129.61, 129.38, 127.21, 122.34, 121.02, 120.31, 110.07; MS calcd for C_15_H_11_ClNO (M+H)^+^ 256.05, found 256.05.

(Z)-3-(3-chlorobenzylidene)indolin-2-one (**2**): Yellow solid; yield: 79.3%; mp: 145.3–146.5 °C; ^1^H NMR (400 MHz, DMSO-*d_6_*): *δ* 10.64 (s, 1H), 7.78–7.64 (m, 2H), 7.57–7.47 (m, 2H), 7.38 (d, *J* = 7.5 Hz, 1H), 7.24–6.96 (m, 2H), 6.87–6.80 (m, 2H). ^13^C NMR (101 MHz, DMSO-*d_6_*): *δ* 165.82, 144.72, 141.08, 138.51, 138.03, 131.95, 131.62, 130.37, 128.75, 127.14, 126.47, 124.24, 122.48, 114.21, 110.57; HRMS calcd for C_15_H_11_ClNO (M+H)^+^ 256.0529, found 256.0531.

(Z)-3-(4-chlorobenzylidene)indolin-2-one (**3**): Yellow solid; yield: 80.5%; mp: 144.6–145.7 °C; ^1^H NMR (400 MHz, DMSO-*d_6_*): *δ* 10.64 (s, 1H), 7.72 (d, *J* = 8.4 Hz, 2H), 7.57 (d, *J* = 8.2 Hz, 3H), 7.47 (d, *J* = 7.6 Hz, 1H), 7.23 (t, *J* = 7.6 Hz, 1H), 6.88–6.83 (m, 2H); ^13^C NMR (101 MHz, DMSO-*d_6_*): *δ* 168.35, 142.95, 134.21, 134.01, 133.23, 131.02, 130.29, 128.75, 128.07, 122.36, 121.13, 120.53, 110.11; HRMS calcd for C_15_H_11_ClNO (M+H)^+^ 256.0529, found 256.0520.

(Z)-5-chloro-3-(4-chlorobenzylidene)indolin-2-one (**4**): Yellow solid; yield: 69.3%; mp: 192.9–193.8 °C; ^1^H NMR (400 MHz, DMSO-*d_6_*): *δ* 8.65 (s, 1H), 8.17 (d, *J* = 6.6 Hz, 1H), 7.92 (s, 1H), 7.67–7.65 (m, 1H), 7.57 (d, *J* = 5.0 Hz, 1H), 7.51 (d, *J* = 6.7 Hz, 1H), 6.89 (d, *J* = 9.0 Hz, 1H), 6.83 (d, *J* = 8.2 Hz, 1H); ^13^C NMR (101 MHz, DMSO-*d_6_*): *δ* 166.69, 141.87, 139.61, 136.74, 135.58, 133.50, 130.93, 130.07, 129.94, 128.79, 127.57, 125.47, 121.83, 120.12, 110.85; HRMS calcd for C_15_H_10_Cl_2_NO (M+H)^+^ 290.0139, found 290.0139.

(Z)-6-chloro-3-(4-chlorobenzylidene)indolin-2-one (**5**): Yellow solid; yield: 65.1%; mp: 195.6–197.1 °C; ^1^H NMR (400 MHz, DMSO-*d_6_*): *δ* 7.84–7.71 (m, 1H), 7.67–7.63 (m, 2H), 7.55–7.49 (m, 2H), 7.37 (d, *J* = 8.2 Hz, 1H), 6.91 (dd, *J* = 10.2, 2.0 Hz, 1H), 6.89–6.84 (m, 1H); ^13^C NMR (101 MHz, DMSO-*d_6_*): *δ* 168.26, 144.46, 136.38, 134.80, 133.51, 130.69, 129.50, 128.89, 127.60, 123.60, 121.01, 120.99, 119.47, 110.20, 109.45; HRMS calcd for C_15_H_10_Cl_2_NO (M+H)^+^ 290.0139, found 290.0140.

### 3.3. General Procedure for Preparation of Target Compounds (**A1–A30**)

Sodium hydroxide (1.96 mmol) and corresponding benzene sulfonyl chloride (3 mmol) were added to a stirred solution of appropriate intermediates **1**–**5** (1.96 mmol) in 10 mL of anhydrous THF at 0 °C. The reaction mixture was stirred for 2 h. The organic solvent of the mixture was removed in vacuo and the residue was purified using column chromatography with petroleum ether/ethyl acetate (40:1–20:1) to obtain **A1**–**A30**.

(E)-3-(2-chlorobenzylidene)-1-(phenylsulfonyl)indolin-2-one (**A1**): solid; yield: *46.9%;* mp: 138.2–139.9 °C; ^1^H NMR (600 MHz, DMSO-*d_6_*): *δ* 8.11 (d, *J* = 7.6 Hz, 2H), 7.92 (d, *J* = 8.3 Hz, 1H), 7.82 (t, *J* = 7.5 Hz, 1H), 7.75–7.70 (m, 4H), 7.66 (d, *J* = 7.7 Hz, 1H), 7.55 (t, *J* = 7.2 Hz, 1H), 7.47 (m, 2H), 7.23 (d, *J* = 7.7 Hz, 1H), 7.10 (t, *J* = 7.7 Hz, 1H); ^13^C NMR (151 MHz, DMSO-*d_6_*): *δ* 165.12, 138.61, 137.49, 135.39, 135.28, 132.89, 132.09, 132.03, 131.35, 130.23, 130.01, 129.93, 127.65, 127.45, 127.31, 126.09, 124.88, 122.86, 120.76, 113.50; HRMS calcd for C_21_H_14_ClNNaO_3_S (M+Na)^+^ 418.0281, found 418.0263.

(E)-3-(2-chlorobenzylidene)-1-(o-tolylsulfonyl)indolin-2-one (**A2**): Yellow solid; yield: 59.3%; mp: 139.9–141.3 °C; ^1^H NMR (600 MHz, DMSO-*d_6_*): *δ* 8.08–7.99 (m, 1H), 7.89 (d, *J* = 8.4 Hz, 1H), 7.79 (d, *J* = 7.1 Hz, 1H), 7.76–7.73 (m, 1H), 7.71 (s, 1H), 7.69–7.66 (m, 1H), 7.57–7.56 (m, 2H), 7.52–7.48 (m, 3H), 7.26 (dd, *J* = 7.5 Hz, 1H), 7.12 (q, *J* = 7.7, 7.5, 6.8 Hz, 1H), 2.52 (s, 3H); ^13^C NMR (151 MHz, DMSO-*d_6_*): *δ* 139.69, 135.89, 135.35, 133.41, 133.29, 132.52, 132.47, 131.68, 131.17, 130.75, 130.70, 130.44, 128.08, 127.92, 127.23, 126.39, 125.15, 123.40, 114.06, 113.91; HRMS calcd for C_22_H_16_ClNNaO_3_S (M+Na)^+^ 432.0437, found 432.0417. 

(E)-3-(2-chlorobenzylidene)-1-(m-tolylsulfonyl)indolin-2-one (**A3**): Yellow solid; yield: 49.1%; mp: 140.2–141.9 °C; ^1^H NMR (600 MHz, DMSO-*d_6_*): *δ* 7.89 (t, *J* = 3.9 Hz, 2H), 7.73–7.71 (m, 2H), 7.64–7.61 (m, 2H), 7.59–7.52 (m, 3H), 7.48–7.44 (m, 2H), 7.20 (d, *J* = 7.7 Hz, 1H), 7.08 (t, *J* = 7.7 Hz, 1H), 2.40 (s, 3H); ^13^C NMR (151 MHz, DMSO-*d_6_*): *δ* 165.50, 140.29, 139.03, 137.85, 136.36, 135.79,133.28, 132.48, 132.45, 131.78, 130.61, 130.42, 130.18, 128.07, 127.80, 126.53, 125.26, 125.07, 123.24, 121.10, 112.93, 21.24; HRMS calcd for C_22_H_16_ClNNaO_3_S (M+Na)^+^ 432.0437, found 432.0417.

(E)-3-(2-chlorobenzylidene)-1-tosylindolin-2-one (**A4**): Yellow solid; yield: 40.4%; mp: 148.9–150.5 °C; ^1^H NMR (600 MHz, DMSO-*d_6_*): *δ* 7.98 (d, *J* = 8.4 Hz, 2H), 7.90 (d, *J* = 8.3 Hz, 1H), 7.73 (d, *J* = 7.6 Hz, 1H), 7.71 (s, 1H), 7.64 (d, *J* = 8.0 Hz, 1H), 7.55–7.52 (m, 1H), 7.50–7.46 (m, 4H), 7.21 (d, *J* = 7.7 Hz, 1H), 7.09 (t, *J* = 7.7 Hz, 1H), 2.39 (s, 3H); ^13^C NMR (151 MHz, DMSO-*d_6_*): *δ* 165.50, 146.62, 139.09, 135.69, 135.03, 133.31, 132.53, 132.42, 131.74, 130.74, 130.64, 130.43, 128.06, 127.93, 126.56, 125.22, 123.25, 121.13, 113.90, 21.65; HRMS calcd for C_22_H_16_ClNNaO_3_S (M+Na)^+^ 432.0437, found 432.0416.

(E)-3-(2-chlorobenzylidene)-1-((2-chlorophenyl)sulfonyl)indolin-2-one (**A5**): Yellow solid; yield: 65.2%; mp: 157.2–158.1 °C; ^1^H NMR (600 MHz, DMSO-*d_6_*): *δ* 8.35 (dd, *J* = 8.0, 1.4 Hz, 1H), 7.82–7.79 (m, 2H), 7.76–7.70 (m, 4H), 7.65 (d, *J* = 8.2 *Hz*, 1H), 7.56–7.54 (m, 1H), 7.50–7.44 (m, 2H), 7.26 (d, *J* = 7.8 Hz, 1H), 7.11 (t, *J* = 7.7 Hz, 1H); ^13^C NMR (151 MHz, DMSO-d_6_): *δ* 165.4, 139.64, 136.92, 136.16, 135.37, 133.38, 133.29, 132.62, 132.52, 132.42, 131.80, 131.62, 130.65, 130.46, 128.65, 128.11, 126.13, 125.23, 123.30, 120.91, 114.44; HRMS calcd for C_21_H_13_Cl_2_NNaO_3_S (M+Na)^+^ 451.9891, found 451.9874.

(E)-3-(2-chlorobenzylidene)-1-((3-chlorophenyl)sulfonyl)indolin-2-one (**A6**): Yellow solid; yield: 45.2%; mp: 164.0–165.4 °C; ^1^H NMR (600 MHz, DMSO-*d_6_*): *δ* 8.11 (t, *J* = 1.8 Hz, 1H), 8.08 (d, J = 8.0 Hz, 1H), 7.91 (d, *J* = 8.2 Hz, 2H), 7.75–7.72 (m, 3H), 7.65 (d, *J* = 8.0 Hz, 1H), 7.56–7.53 (m, 1H), 7.50–7.46 (m, 2H), 7.23 (d, *J* = 7.6 Hz, 1H), 7.11 (t, *J* = 7.4 Hz, 1H); ^13^C NMR (151 MHz, DMSO-*d_6_*): *δ* 165.58, 138.84, 135.91, 135.67, 134.67, 133.34, 132.51, 132.48, 132.40, 131.75, 130.64, 130.45, 128.08, 127.52, 126.76, 126.48, 125.39, 123.28, 121.33, 113.94; HRMS calcd for C_21_H_13_Cl_2_NNaO_3_S (M+Na)^+^ 451.9891, found 451.9875. 

(E)-3-(2-chlorobenzylidene)-1-((4-chlorophenyl)sulfonyl)indolin-2-one (**A7**): Yellow solid; yield: 43.6%; mp: 172.4–173.1 °C; ^1^H NMR (600 MHz, DMSO-*d_6_*): δ 8.11 (d, *J* = 8.7 Hz, 1H), 7.98 (d, *J* = 8.6 Hz, 1H), 7.89 (d, *J* = 8.3 Hz, 1H), 7.77 (d, *J* = 8.7 Hz, 2H), 7.71–7.70 (m, 2H), 7.65 (d, *J* = 8.0 Hz, 1H), 7.59 (d, *J* = 8.4 Hz, 1H), 7.56–7.53 (m, 1H), 7.36 (d, *J* = 8.3 Hz, 1H), 7.23 (d, *J* = 7.7 Hz, 1H), 7.10 (t, *J* = 7.7 Hz, 1H); ^13^C NMR (151 MHz, DMSO-*d_6_*): *δ* 165.56, 139.93, 139.57, 135.84, 133.33, 132.47, 131.75, 130.63, 130.49, 130.44, 129.95, 129.91, 128.15, 128.07, 127.94, 125.36, 123.29, 113.87; HRMS calcd for C_21_H_14_Cl_2_NO_3_S (M+H)^+^ 430.0071, found 430.1069.

(E)-3-(3-chlorobenzylidene)-1-(phenylsulfonyl)indolin-2-one (**A8**): Yellow solid; yield: 60.2%; mp: 115.1–117.3 °C; ^1^H NMR (600 MHz, DMSO-*d_6_*): *δ* 8.07 (d, *J* = 7.7 Hz, 2H), 8.04 (d, *J* = 7.7 Hz, 1H), 7.98 (s, 1H), 7.91 (d, *J* = 8.2 Hz, 1H), 7.86–7.84 (m, 1H), 7.79 (t, *J* = 7.4 Hz, 1H), 7.72 (s, 1H), 7.70–7.68 (m, 1H), 7.63–7.61 (m, 1H), 7.51 (d, *J* = 7.7 Hz, 2H), 7.44 (t, *J* = 7.6 Hz, 1H), 7.10 (t, *J* = 7.7 Hz, 1H); ^13^C NMR (151 MHz, DMSO-*d_6_*): *δ* 165.20, 138.37, 137.84, 137.35, 135.57, 135.01, 134.88, 133.43, 130.97, 130.59, 130.03, 129.91, 129.68, 129.65, 128.77, 127.44, 124.59, 122.39, 120.75, 113.30; HRMS calcd for C_21_H_15_ClNO_3_S (M+H)^+^ 396.0461, found 396.0459.

(E)-3-(3-chlorobenzylidene)-1-(o-tolylsulfonyl)indolin-2-one (**A9**): Yellow solid; yield: 50.7%; mp: 140.9–143.2 °C; ^1^H NMR (400 MHz, DMSO-*d_6_*): *δ* 8.20–8.13 (m, 1H), 8.05 (s, 1H), 8.00–7.93 (m, 1H), 7.92–7.90 (m, 1H), 7.74–7.71 (m, 2H), 7.66 (d, *J* = 7.5 Hz, 1H), 7.56–7.51 (m, 3H), 7.49–7.44 (m, 2H), 7.16–7.10 (m, 1H); ^13^C NMR (101 MHz, DMSO-*d_6_*): *δ* 137.94, 135.62, 134.53, 133.44, 132.75, 130.89, 130.63, 130.06, 129.93, 128.79, 127.51, 127.27, 126.57, 124.45, 120.73, 120.59, 19.46; HRMS calcd for C_21_H_16_ClNNaO_3_S (M+Na)^+^ 432.0437, found 432.0428.

(E)-3-(3-chlorobenzylidene)-1-(m-tolylsulfonyl)indolin-2-one (**A10**): Yellow solid; yield: 59.6%; mp: 147.4–149.2 °C; ^1^H NMR (600 MHz, DMSO-*d_6_*): *δ* 8.11 (d, 1H), 7.91–7.85 (m, 4H), 7.72 (m, 1H), 7.63–7.59 (m, 1H), 7.57–7.50 (m, 3H), 7.48–7.43 (m, 2H), 7.29–7.10 (m, 1H), 2.37 (d, 3H); ^13^C NMR (151 MHz, DMSO-*d_6_*): *δ* 165.76, 139.02, 138.41, 137.94, 136.27, 136.21, 136.15, 134.03, 131.57, 131.31, 131.21, 131.00, 130.65, 130.51, 130.11, 129.38, 128.05, 127.81, 127.63, 125.23, 125.16, 125.06, 125.02, 122.99, 113.55, 21.25; HRMS calcd for C_21_H_16_ClNNaO_3_S (M+Na)^+^ 432.0437, found 432.0428.

(E)-3-(3-chlorobenzylidene)-1-tosylindolin-2-one (**A11**): Yellow solid; yield: 51.5%; mp: 136.3–138.1 °C; ^1^H NMR (600 MHz, DMSO-*d_6_*): *δ* 8.00 (d, *J* = 5.5 Hz, 1H), 7.96 (d, *J* = 8.2 Hz, 1H), 7.91 (t, *J* = 8.3 Hz, 1H), 7.86 (q, *J* = 8.3 Hz, 1H), 7.73 (d, *J* = 5.0 Hz, 1H), 7.65–7.55 (m, 2H), 7.53–7.49 (m, 3H), 7.48–7.44 (m, 2H), 7.30–7.12 (m, 1H); ^13^C NMR (151 MHz, DMSO-*d_6_*): *δ* 165.36, 146.13, 137.94, 130.87, 130.80, 130.27, 130.24, 130.08, 128.96, 127.63, 127.45, 127.38, 127.74, 124.13, 120.89, 113.48, 21.19; HRMS calcd for C_22_H_17_ClNO_3_S (M+H)^+^ 410.0618, found 410.0612.

(E)-3-(3-chlorobenzylidene)-1-((2-chlorophenyl)sulfonyl)indolin-2-one (**A12**): Yellow solid; yield: 45.3%; mp: 125.1–126.3 °C; ^1^H NMR (400 MHz, DMSO-*d_6_*): *δ* 8.34 (t, *J* = 6.7, 1.2 Hz, 1H), 7.81 (t, *J* = 8.1 Hz, 2H), 7.72 (m, 4H), 7.55 (d, *J* = 7.0 Hz, 3H), 7.48–7.44 (m, 2H), 7.15 (t, *J* = 7.7 Hz, 1H); ^13^C NMR (101 MHz, DMSO-*d_6_*): *δ* 165.01, 139.04, 138.19, 137.54, 136.20, 135.50, 134.86, 133.45, 132.78, 131.95, 131.18, 130.81, 130.63, 130.34, 130.00, 128.82, 127.94, 127.49, 124.51, 122.42, 120.45, 113.83, 113.46; HRMS calcd for C_21_H_14_Cl_2_NO_3_S (M+H)^+^ 430.0071, found 430.0065.

(E)-3-(3-chlorobenzylidene)-1-((3-chlorophenyl)sulfonyl)indolin-2-one (**A13**): Yellow solid; yield: 48.8%; mp: 146.7–148.0 °C; ^1^H NMR (400 MHz, DMSO-*d_6_*): *δ* 8.21–8.12 (m, 2H), 8.06 (s, 1H), 7.95 (dd, *J* = 7.0 Hz, 1H), 7.82–7.74 (m, 2H), 7.71–7.65 (m, 1H), 7.53–7.47 (m, 4H), 7.33–7.25 (m, 1H), 7.15–7.11 (m, 1H); ^13^C NMR (101 MHz, DMSO-*d_6_*): *δ* 163.86, 139.36, 138.66, 137.59, 135.42. 133.36, 131.31, 131.24, 130.66, 128.08, 125.81, 125.78, 125.24, 124.61, 121.20, 114.08, 113.72; HRMS calcd for C_21_H_13_Cl_2_NNaO_3_S (M+Na)^+^ 451.9891, found 451.9897.

(E)-3-(3-chlorobenzylidene)-1-((4-chlorophenyl)sulfonyl)indolin-2-one (**A14**): Yellow solid; yield: 39.2%; mp: 137.5–139.6 °C; ^1^H NMR (400 MHz, DMSO-*d_6_*): *δ* 8.32 (d, *J* = 7.9 Hz, 1H), 8.10 (d, *J* = 10.3 Hz, 2H), 7.94 (d, *J* = 7.7 Hz, 1H), 7.80–7.75 (m, 2H), 7.74–7.68 (m, 3H), 7.53 (d, *J* = 8.5 Hz, 2H), 7.44 (t, *J* = 7.6 Hz, 1H), 7.30 (t, *J* = 7.6 Hz, 1H); ^13^C NMR (101 MHz, DMSO-*d_6_*): *δ* 139.53, 137.39, 136.09, 133.54, 132.69, 131.89, 131.06, 129.63, 128.38, 128.02, 124.50, 120.51, 113.41; HRMS calcd for C_22_H_13_Cl_2_NNaO_3_S (M+Na)^+^ 451.9891, found 451.9884.

(E)-1-((2-bromophenyl) sulfonyl)-3-(3-chlorobenzylidene))indolin-2-one (**A15**): Yellow solid; yield: 41.6%; mp: 139.2–140.3 °C; ^1^H NMR (400 MHz, DMSO-*d_6_*): *δ* 8.37 (dd, *J* = 7.8, 1.7 Hz, 1H), 7.82 (d, *J* = 8.2 Hz, 1H), 7.73–7.69 (m, 3H), 7.69–7.65 (m, 2H), 7.57–7.54 (m, 3H), 7.48–7.44 (m, 2H), 7.14 (td, *J* = 7.7, 0.76 Hz, 1H); ^13^C NMR (101 MHz, DMSO-*d_6_*): *δ* 164.96, 139.20, 138.11, 136.54, 136.00, 135.44, 135.40, 135.35, 133.41, 133.11, 130.75, 130.73, 130.58, 130.31, 130.00, 129.96, 128.79, 128.40, 128.33, 127.45, 124.56, 124.41, 122.36, 120.39, 119.58, 114.04; HRMS calcd for C_21_H_13_ClBrNNaO_3_S (M+Na)^+^ 495.9386, found 495.9383.

(E)-1-((3-bromophenyl) sulfonyl)-3-(3-chlorobenzylidene))indolin-2-one (**A16**): Yellow solid; yield: 47.4 %; mp: 111.3–112.9 °C; ^1^H NMR (400 MHz, DMSO-*d_6_*): *δ* 8.24–8.21 (m, 1H), 8.17–8.08 (m, 1H), 8.06–7.92 (m, 2H), 7.91–7.84 (m, 1H), 7.77–7.64 (m, 3H), 7.63–7.54 (m, 2H), 7.51–7.45 (q, 2H), 7.32–7.09 (m, 1H); ^13^C NMR (101 MHz, DMSO-*d_6_*): *δ* 165.27, 139.03, 138.06, 137.92, 135.59, 131.00, 130.74, 130.66, 130.12, 130.07, 129.62, 128.82, 127.49, 126.43, 124.81, 124.75, 123.88, 122.43, 122.25, 120.92, 113.33, 112.94; HRMS calcd for C_21_H_13_ClBrNNaO_3_S (M+Na)^+^ 495.9386, found 495.9385.

(E)-1-((4-bromophenyl) sulfonyl)-3-(3-chlorobenzylidene))indolin-2-one (**A17**): Yellow solid; yield: 50.5%; mp: 131.7–132.5 °C; ^1^H NMR (400 MHz, DMSO-*d_6_*): *δ* 8.25–8.02 (m, 2H), 7.98 (d, *J* = 3.9 Hz, 1H), 7.97–7.92 (m, 1H), 7.92–7.83 (m, 3H), 7.73 (d, *J* = 7.8 Hz, 1H), 7.66–7.49 (m, 3H), 7.49–7.45 (m, 1H), 7.32–7.12 (td, *J* = 7.6 Hz, 1H); ^13^C NMR (101 MHz, DMSO-*d_6_*): *δ* 165.20, 138.54, 137.92, 136.68, 136.51, 136.42, 135.55, 132.77, 130.69, 130.62, 130.40, 129.24, 128.76, 127.44, 124.99, 124.74, 124.68, 122.41, 120.85, 120.61, 113.25, 112.85; HRMS calcd for C_21_H_13_ClBrNNaO_3_S (M+Na)^+^ 495.9386, found 495.9388.

(E)-3-(3-chlorobenzylidene)-1-((2-fluorophenyl)sulfonyl)indolin-2-one (**A18**): Yellow solid; yield: 36.7%; mp: 158.2–160.3 °C; ^1^H NMR (400 MHz, DMSO-*d_6_*): *δ* 8.23–8.02 (m, 4H), 7.90–7.85 (m, 3H), 7.76–7.72 (m, 2H), 7.55–7.46 (m, 3H), 7.24–7.10 (m, 1H); ^13^C NMR (101 MHz, DMSO-*d_6_*): *δ* 165.23, 138.96, 138.57, 137.98, 134.87, 134.04, 133.43, 131.74, 130.62, 130.05, 128.76, 127.44, 126.87, 126.06, 124.77, 124.71, 122.41, 120.90, 120.62, 113.32; HRMS calcd for C_21_H_14_ClFNO_3_S (M+H)^+^ 414.0367, found 414.0364.

(E)-3-(3-chlorobenzylidene)-1-((3-fluorophenyl)sulfonyl)indolin-2-one (**A19**): Yellow solid; yield: 35.0%; mp: 176.6–178.2 °C; ^1^H NMR (400 MHz, DMSO-*d_6_*): *δ* 8.23–7.98 (m, 2H), 7.94–7.82 (m, 2H), 7.74–7.62 (m, 4H), 7.53 (d, *J* = 5.5 Hz, 2H), 7.46 (t, *J* = 8.4 Hz, 2H), 7.12 (t, *J* = 7.3 Hz, 1H); ^13^C NMR (101 MHz, DMSO-*d_6_*): *δ* 165.02, 138.41, 137.97, 137.78, 135.59, 133.25, 132.60, 131.99, 130.79, 130.54, 130.45, 129.87, 129.76, 128.58, 127.27, 124.82, 124.53, 123.40, 122.22, 120.69, 113.18, 112.78; HRMS calcd for C_21_H_13_ClFNNaO_3_S (M+Na)^+^ 436.0186, found 436.0184.

(E)-3-(3-chlorobenzylidene)-1-((4-fluorophenyl)sulfonyl)indolin-2-one (**A20**): Yellow solid; yield: 40.1%; mp: 141.7–143.1 °C; ^1^H NMR (400 MHz, DMSO-*d_6_*): *δ* 8.20–8.12 (m, 3H), 8.00 (d, *J* = 7.6 Hz, 1H), 7.93–7.84 (m, 2H), 7.73 (d, *J* = 9.1 Hz, 2H), 7.64 (d, *J* = 7.6 Hz, 1H), 7.55–7.48 (m, 3H), 7.12 (q, *J* = 7.6 Hz, 1H); ^13^C NMR (101 MHz, DMSO-*d_6_*): *δ* 165.37, 138.65, 138.43, 138.01, 136.90, 135.72, 135.01, 133.72, 133.58, 132.93, 130.85, 128.93, 127.60, 124.84, 124.78, 122.55, 117.26, 117.03, 113.43, 106.96; HRMS calcd for C_21_H_13_ClFNNaO_3_S (M+Na)^+^ 436.0186, found 436.0185.

(E)-3-(3-chlorobenzylidene)-1-((3-(trifluoromethyl)phenyl)sulfonyl)indolin-2-one (**A21**): Yellow solid; yield: 38.9%; mp: 182.3–183.7 °C; ^1^H NMR (400 MHz, DMSO-*d_6_*): *δ* 8.18 (t, *J* = 3.6 Hz, 1H), 7.81 (s, 1H), 7.74–7.64 (m, 3H), 7.56–7.49 (m, 4H), 7.32 (d, *J* = 6.9 Hz, 1H), 7.26–7.20 (m, 1H), 7.00 (t, *J* = 7.6 Hz, 1H), 6.83 (d, *J* = 7.7 Hz, 1H); ^13^C NMR (101 MHz, DMSO-*d_6_*): *δ* 166.86, 140.93, 135.79, 135.49, 134.66, 133.37, 132.72, 130.67, 130.57, 130.52, 130.45, 129.87, 129.72, 129.35, 128.69, 128.03, 127.50, 124.41, 121.07, 119.99, 109.38; HRMS calcd for HRMS calcd for C_22_H_14_ClF_3_NO_3_S (M+H)^+^ 464.0335, found 464.0331.

(E)-3-(3-chlorobenzylidene)-1-((3-nitrophenyl)sulfonyl)indolin-2-one (**A22**): Yellow solid; yield: 48.3%; mp: 195.3–197.0 °C; ^1^H NMR (400 MHz, DMSO-*d_6_*): *δ* 8.75–8.60 (m, 2H), 8.52 (d, *J* = 8.0 Hz, 1H), 8.03–7.87 (m, 3H), 7.78 (d, *J* = 8.7 Hz, 1H), 7.71 (d, *J* = 8.1 Hz, 1H), 7.55 (d, *J* = 6.5 Hz, 2H), 7.50 (d, *J* = 5.0 Hz, 2H), 7.16 (t, *J* = 7.6 Hz, 1H); ^13^C NMR (101 MHz, DMSO-*d_6_*): *δ* 165.31, 147.82, 138.45, 138.12, 138.02, 135.55, 133.35, 131.68, 131.00, 130.66, 129.98, 129.45, 128.76, 127.44, 122.49, 122.45, 121.03, 113.23, 109.37; HRMS calcd for HRMS calcd for C_21_H_13_ClN_2_NaO_5_S (M+Na)^+^ 463.0131, found 463.0131.

(E)-3-(3-chlorobenzylidene)-1-((4-methoxyphenyl)sulfonyl)indolin-2-one (**A23**): Yellow solid; yield: *46.6%;* mp: 175.1–176.7 °C; ^1^H NMR (400 MHz, DMSO-*d_6_*): *δ* 8.05–8.00 (m, 2H), 7.91 (dd, *J* = 8.1, 3.1 Hz, 1H), 7.73–7.70 (m, 1H), 7.64 (d, *J* = 7.1 Hz, 1H), 7.55–7.49 (m, 2H), 7.47–7.43 (m, 2H), 7.20–7.14 (m, 3H), 7.09 (m, 1H); ^13^C NMR (101 MHz, DMSO-*d_6_*): *δ* 164.21, 138.63, 137.67, 135.69, 135.01, 133.48, 131.17, 130.67, 130.09, 129.94, 129.89, 129.84, 129.75, 128.84, 127.51, 126.07, 124.58, 122.68, 120.54, 114.91, 114.86, 114.83, 113.33, 55.88; HRMS calcd for HRMS calcd for C_22_H_17_ClNO_4_S (M+H)^+^ 426.0567, found 426.0566.

(E)-3-(4-chlorobenzylidene)-1-(o-tolylsulfonyl)indolin-2-one (**A24**): Yellow solid; yield: 41.2%; mp: 155.2–157.1 °C; ^1^H NMR (400 MHz, DMSO-*d_6_*): *δ* 8.17–8.10 (m, 2H), 7.98–7.89 (m, 1H), 7.87–7.82 (m, 1H), 7.73–7.68 (m, 2H), 7.58–7.49 (m, 3H), 7.42 (t, *J* = 8.1 Hz, 3H), 7.15–7.09 (m, 1H), 2.43 (d, 3H); ^13^C NMR (101 MHz, DMSO-*d_6_*): *δ* 165.18, 138.92, 138.34, 134.88, 134.58, 133.52, 132.71, 132.23, 131.12, 131.06, 130.69, 130.52, 130.03, 128.82, 128.30, 127.23, 1265.52, 124.51, 124.41, 122.55, 122.39, 113.38, 113.23, 19.43; HRMS calcd for C_22_H_16_ClNNaO_3_SN_3_O_2_ (M+Na)^+^ 432.0437, found 432.0431.

(E)-3-(4-chlorobenzylidene)-1-(m-tolylsulfonyl)indolin-2-one (**A25**): Yellow solid; yield: 50.9%; mp: 149.3–151.7 °C; ^1^H NMR (400 MHz, DMSO-*d_6_*): *δ* 8.15 (d, *J* = 8.6 Hz, 1H), 7.92–7.85 (m, 4H), 7.73 (t, *J* = 8.4 Hz, 2H), 7.59–7.55 (m, 4H), 7.48–7.43 (m, 1H), 7.30–7.11 (td, *J* = 7.6 Hz, 1H), 2.39 (d, 3H); ^13^C NMR (101 MHz, DMSO-*d_6_*): *δ* 139.56, 138.96, 138.28, 135.66, 133.53, 132.30, 131.13, 130.84, 129.52, 128.88, 128.37, 127.20, 127.04, 124.60, 124.44, 123.11, 122.46, 113.29, 112.93, 20.66; HRMS calcd for C_22_H_16_ClNNaO_3_SN_3_O_2_ (M+Na)^+^ 432.0437, found 432.0429.

(E)-3-(4-chlorobenzylidene)-1-tosylindolin-2-one (**A26**): Yellow solid; yield: 43.2%; mp: 134.1–135.6 °C; ^1^H NMR (400 MHz, DMSO-*d_6_*): *δ* 8.13 (d, *J* = 8.4 Hz, 1H), 7.99–7.85 (m, 4H), 7.70 (t, *J* = 5.8, 8.4 Hz, 2H), 7.56 (d, *J* = 8.4 Hz, 3H), 7.46 (d, *J* = 8.3 Hz, 2H), 7.20 (dd, *J* = 7.2 Hz, 1H), 2.36 (d, 3H); ^13^C NMR (101 MHz, DMSO-*d_6_*): *δ* 138.75, 133.36, 130.95, 129.92, 128.70, 128.18, 127.10, 124.43, 20.87; HRMS calcd for C_22_H_16_ClNNaO_3_SN_3_O_2_ (M+Na)^+^ 432.0437, found 432.0431.

(E)-3-(4-chlorobenzylidene)-1-((3-chlorophenyl)sulfonyl)indolin-2-one (**A27**): Yellow solid; yield: 44.9%; mp: 184.8–185.6 °C; ^1^H NMR (400 MHz, DMSO-*d_6_*): *δ* 8.09 (s, 1H), 8.08 (d, *J* = 8.1 Hz, 1H), 7.92–7.89 (m, 2H), 7.77 (s, 1H), 7.74–7.70 (m, 3H), 7.58 (d, *J* = 8.4 Hz, 3H), 7.46 (t, *J* = 8.0 Hz, 1H), 7.14 (t, *J* = 7.7 Hz, 1H); ^13^C NMR (101 MHz, DMSO-*d_6_*): *δ* 138.51, 135.01, 131.77, 131.15, 130.85, 128.89, 126.88, 126.07, 124.78, 113.28; HRMS calcd for C_22_H_13_Cl_2_NNaO_3_S (M+Na)^+^ 451.9891, found 451.9885.

(E)-3-(4-chlorobenzylidene)-1-(phenylsulfonyl)indolin-2-one (**A28**): Yellow solid; yield: 36.8%; mp: 135.4–136.8 °C; ^1^H NMR (600 MHz, DMSO-*d_6_*): *δ* 8.13 (d, *J* = 6.5 Hz, 1H), 8.08–7.99 (m, 2H), 7.91 (d, *J* = 7.1 Hz, 1H), 7.78–7.65 (m, 5H), 7.54 (m, 3H), 7.44 (d, *J* = 6.5 Hz, 1H), 7.11 (s, 1H); ^13^C NMR (151 MHz, DMSO-*d_6_*): *δ* 165.94, 139.61, 138.90, 136.38, 135.60, 135.49, 134.13, 132.83, 131.71, 131.43, 130.40, 130.28, 130.23, 128.93, 127.78, 125.22, 124.95, 123.05, 121.06, 113.85, 113.48; HRMS calcd for C_21_H_14_ClNNaO_3_S (M+Na)^+^ 418.0281 found 418.0270.

(E)-5-chloro-3-(3-chlorobenzylidene)-1-((3-chlorophenyl)sulfonyl)indolin-2-one (**A29**): Yellow solid; yield: 39.3%; mp: 177.8–178.7 °C; ^1^H NMR (400 MHz, DMSO-*d_6_*): *δ* 8.11 (d, 1H), 8.05–8.02 (m, 2H), 7.91 (d, J = 8.7 Hz, ^1^H), 7.88–7.80 (m, 2H), 7.75 (s, 1H), 7.73–7.65 (m, 1H), 7.58–7.48 (m, 3H), 7.39–7.36 (m, 1H); ^13^C NMR (101 MHz, DMSO-*d_6_*): *δ* 163.09, 140.38, 138.92, 135.33, 135.05, 134.73, 134.07, 132.92, 131.79, 131.76, 131.14, 130.98, 130.72, 130.26, 129.41, 129.34, 126.85, 126.24, 123.01, 120.59, 114.58; HRMS calcd for HRMS calcd for C_22_H_12_Cl_3_NNaO_3_S (M+H)^+^ 485.9501, found 485.9499.

(E)-6-chloro-3-(3-chlorobenzylidene)-1-((3-chlorophenyl)sulfonyl)indolin-2-one (**A30**): Yellow solid; yield: 42.8%; mp: 132.6–133.8 °C; ^1^H NMR (400 MHz, DMSO-*d_6_*): *δ* 8.14–8.08 (m, 2H), 7.94–7.91 (m, 2H), 7.80 (s, 1H), 7.76–7.71 (m, 2H), 7.65 (d, *J* = 7.1 Hz, 1H), 7.58–7.53 (m, 2H), 7.49 (d, *J* = 8.4 Hz, 1H), 7.25 (dd, *J* = 8.4, 2.0 Hz, 1H); ^13^C NMR (101 MHz, DMSO-*d_6_*): *δ* 139.39, 139.27, 136.01, 134.64, 134.11, 133.43, 132.31, 131.49, 131.34, 131.06, 130.78, 129.46, 128.08, 127.05, 125.27; HRMS calcd for HRMS calcd for C_22_H_12_Cl_3_NNaO_3_S (M+H)^+^ 485.9501, found 485.9501.

### 3.4. General Procedure for Preparation of Target Compounds **B1**–**B10**

Based on the procedure for compounds **A1**–**A30**, target compounds **B1**–**B10** were obtained from intermediates **1**–**3** and the corresponding benzoyl chloride in the presence of NaOH in THF.

(E)-1-(2-chlorobenzoyl)-3-(2-chlorobenzylidene)indolin-2-one (**B1**): Yellow solid; yield: 27.9%; mp: 214.5–216.2 °C; ^1^H NMR (400 MHz, DMSO-*d_6_*): *δ* 7.78 (d, *J* = 7.2 Hz, 1H), 7.69 (s, 1H), 7.67–7.63 (m, 2H), 7.57–7.55 (m, 2H), 7.54–7.51 (m, 3H), 7.50–7.46 (m, 2H), 7.33 (d, *J* = 7.7 Hz, 1H), 7.17 (t, *J* = 7.6 Hz, 1H); ^13^C NMR (101 MHz, DMSO-*d_6_*): *δ* 165.68, 139.39, 135.81, 134.45, 132.80, 132.46, 132.14, 131.77, 131.36, 130.87, 130.70, 130.50, 130.01, 129.88, 129.17, 129.02, 128.54, 127.51, 127.21, 127.10, 126.52, 125.23, 122.28, 121.42, 115.72; HRMS calcd for C_22_H_13_Cl_2_NNaO_2_ (M+Na)^+^ 416.0221, found 416.0213.

(E)-1-(3-chlorobenzoyl)-3-(2-chlorobenzylidene)indolin-2-one (**B2**): Yellow solid; yield: 37.3%; mp: 219.0–220.8 °C; ^1^H NMR (400 MHz, DMSO-*d_6_*): *δ* 8.26 (d, *J* = 8.1 Hz, 1H), 7.79–7.77 (m, 1H), 7.69–7.63 (m, 3H), 7.56–7.54 (m, 3H), 7.53–7.51 (m, 2H), 7.49–7.47 (m, 1H), 7.33 (d, *J* = 7.5 Hz, 1H), 7.17 (t, *J* = 7.6 Hz, 1H); ^13^C NMR (101 MHz, DMSO-*d_6_*): *δ* 165.65, 139.36, 135.78, 134.41, 132.78, 132.11, 131.72, 131.32, 130.82, 130.68, 130.47, 129.97, 129.84, 128.99, 128.50, 127.46, 127.17, 127.06, 125.18, 122.24, 121.39, 115.69; HRMS calcd for C_22_H_13_Cl_2_NNaO_2_ (M+Na)^+^ 416.0221, found 416.0204.

(E)-1-(4-chlorobenzoyl)-3-(2-chlorobenzylidene)indolin-2-one (**B3**): Yellow solid; yield: 33.5%; mp: 221.3–222.7 °C; ^1^H NMR (400 MHz, DMSO-*d_6_*): *δ* 8.36 (dd, *J* = 7.8, 1.4 Hz, 1H), 7.83–7.79 (m, 2H), 7.77–7.70 (m, 4H), 7.65 (dd, *J* = 8.0, 1.1 Hz, 1H), 7.56–7.53 (m, 1H), 7.51–7.44 (m, 2H), 7.27 (d, *J* = 7.6 Hz, 1H), 7.11 (td, *J* = 7.7, 0.68 Hz, 1H); ^13^C NMR (101 MHz, DMSO-*d_6_*): *δ* 164.78, 139.07, 136.27, 135.51, 134.81, 132.78, 132.71, 132.00, 131.88, 131.84, 131.21, 130.98, 130.05, 129.84, 128.02, 127.47, 125.53, 124.59, 122.69, 120.32, 113.84; HRMS calcd for C_22_H_13_Cl_2_NNaO_2_ (M+Na)^+^ 416.0221, found 416.0205.

(E)-3-(2-chlorobenzylidene)-1-(2-methylbenzoyl)indolin-2-one (**B4**): Yellow solid; yield: 36.7%; mp: 111.2–112.8 °C; ^1^H NMR (600 MHz, DMSO-*d_6_*): *δ* 8.09 (d, *J* = 7.7 Hz, 1H), 7.74 (d, *J* = 7.0 Hz, 1H), 7.62 (s, 1H), 7.52 (d, *J* = 7.0 Hz, 1H), 7.50–7.41 (m, 5H), 7.30 (d, *J* = 7.7 Hz, 2H), 7.29–7.26 (m, 1H), 7.13–6.98 (m, 1H), 2.29 (s, 3H); ^13^C NMR (151 MHz, DMSO-*d_6_*): *δ* 166.40, 140.43, 136.20, 135.46, 134.55, 133.33, 132.75, 132.29, 131.29, 130.98, 130.81, 130.55, 130.43, 128.06, 127.87, 127.48, 126.02, 125.39, 122.76, 121.85, 116.07; HRMS calcd for C_23_H_16_ClNNaO_2_ (M+Na)^+^ 396.0767, found 396.0750.

(E)-3-(2-chlorobenzylidene)-1-(3-methylbenzoyl)indolin-2-one (**B5**): Yellow solid; yield: 17.6%; mp: 128.2–129.7 °C; ^1^H NMR (400 MHz, DMSO-*d_6_*): *δ* 7.79–7.74 (m, 4H), 7.72 (s, 1H), 7.68–7.50 (m, 3H), 7.49–7.44 (m, 1H), 7.42 (t, *J* = 7.1 Hz, 3H), 7.36 (d, *J* = 7.5 Hz, 1H), 2.35 (s, 3H); ^13^C NMR (101 MHz, DMSO-*d_6_*): *δ* 167.28, 137.75, 133.31, 130.57, 129.59, 128.30, 126.31; HRMS calcd for C_23_H_16_ClNNaO_2_ (M+Na)^+^ 396.0767, found 396.0763.

(E)-3-(2-chlorobenzylidene)-1-(4-methylbenzoyl)indolin-2-one (**B6**): Yellow solid; yield: 20.7%; mp: 145.3–146.9 °C; ^1^H NMR (400 MHz, DMSO-*d_6_*): *δ* 7.80 (d, *J* = 6.6 Hz, 1H), 7.76–7.72 (m, 3H), 7.68 (s, 2H), 7.54 (t, *J* = 7.7, 6.6 Hz, 2H), 7.42 (t, *J* = 7.6 Hz, 1H), 7.34–7.29 (m, 3H), 7.10 (t, *J* = 7.6 Hz, 1H), 2.41 (s, 3H); ^13^C NMR (101 MHz, DMSO-*d_6_*): *δ* 157.01, 133.73, 131.65, 130.52, 129.71, 128.65, 127.50, 124.26, 122.28, 114.65, 21.24; HRMS calcd for C_23_H_16_ClNNaO_2_ (M+Na)^+^ 396.0767, found 396.0760.

(E)-1-(2-chlorobenzoyl)-3-(3-chlorobenzylidene)indolin-2-one (**B7**): Yellow solid; yield: 26.9%; mp: 108.3–109.7 °C; ^1^H NMR (400 MHz, DMSO-*d_6_*): *δ* 8.26 (d, *J* = 8.0 Hz, 1H), 7.75 (s, 1H), 7.72–7.68 (m, 2H), 7.64–7.60 (m, 2H), 7.58–7.54 (m, 3H), 7.51–7.46 (m, 3H), 7.22 (td, *J* = 7.7, 0.88 Hz, 1H); ^13^C NMR (101 MHz, DMSO-*d_6_*): *δ* 165.59, 139.27, 137.03, 135.79, 133.47, 131.33, 130.72, 130.68, 129.86, 129.13, 128.98, 128.78, 128.49, 127.43, 127.18, 125.66, 125.17, 122.02, 121.53, 115.65; HRMS calcd for C_22_H_13_Cl_2_NNaO_2_ (M+Na)^+^ 416.0221, found 416.0224.

(E)-1-(3-chlorobenzoyl)-3-(3-chlorobenzylidene)indolin-2-one (**B8**): Yellow solid; yield: 19.9%; mp: 112.4–114.0 °C; ^1^H NMR (400 MHz, DMSO-*d_6_*): *δ* 7.92–7.87 (m, 3H), 7.75 (s, 2H), 7.71–7.68 (m, 3H), 7.57 (d, *J* = 3.6 Hz, 2H), 7.52 (d, *J* = 7.7 Hz, 1H), 7.44 (t, *J* = 7.6 Hz, 1H); ^13^C NMR (101 MHz, DMSO-*d_6_*): *δ* 166.04, 140.08, 136.49, 136.07, 133.55, 132.66, 132.01, 130.76, 130.60, 130.48, 129.99, 128.84, 128.79, 128.76, 127.87, 127.67, 127.52, 124.62, 122.04, 121.63, 115.16; HRMS calcd for C_22_H_13_Cl_2_NNaO_2_ (M+Na)^+^ 416.0221, found 416.0222.

(E)-1-(4-chlorobenzoyl)-3-(3-chlorobenzylidene)indolin-2-one (**B9**): Yellow solid; yield: 22.5%; mp: 103.5–104.8 °C; ^1^H NMR (400 MHz, DMSO-*d_6_*): *δ* 7.93 (d, *J* = 8.2 Hz, 1H), 7.85 (t, *J* = 7.8, 8.1 Hz, 2H), 7.76 (s, 1H), 7.71 (s, 2H), 7.58 (d, *J* = 5.7 Hz, 5H), 7.45 (t, *J* = 7.7 Hz, 1H), 7.16 (t, *J* = 7.6 Hz, 1H); ^13^C NMR (101 MHz, DMSO-*d_6_*): *δ* 167.80, 140.24, 137.49, 136.60, 136.17, 133.63, 133.24, 131.36, 131.20, 130.88, 130.60, 129.93, 128.94, 128.81, 128.27, 127.62, 126.63, 124.65, 122.17, 121.64, 115.12; HRMS calcd for C_22_H_13_Cl_2_NNaO_2_ (M+Na)^+^ 416.0221, found 416.0220.

(E)-1-(3-chlorobenzoyl)-3-(4-chlorobenzylidene)indolin-2-one (**B10**): Yellow solid; yield: 27.1%; mp: 141.3–142.3 °C; ^1^H NMR (400 MHz, DMSO-*d_6_*): *δ* 8.21 (dd, *J* = 8.1, 8.0 Hz, 1H), 8.07 (t, *J* = 8.6, 6.2 Hz, 1H), 7.97–7.75 (m, 1H), 7.73–7.68 (m, 2H), 7.63–7.59 (m, 2H), 7.56–7.51 (m, 3H), 7.49–7.44 (m, 2H), 7.38–7.20 (m, 1H); ^13^C NMR (101 MHz, DMSO-*d_6_*): *δ* 166.20, 138.06, 136.43, 135.35, 133.99, 133.02, 131.67, 131.14, 129.48, 129.07, 128.92, 127.77, 125.76, 125.71, 125.56, 122.65, 122.19, 116.16; HRMS calcd for C_22_H_13_Cl_2_NNaO_2_ (M+Na)^+^ 416.0221, found 416.0223.

### 3.5. In Vitro Enzymatic Assays 

Dose-dependent MDM2 and MDMX binding evaluations were carried out using the fluorescence polarization assay. 

A total of 30 nM preincubated (30 min) MDM2 (or MDMX) and 10 nM PMDM-F peptide in reaction buffer (100 μL, potassium phosphate, 100 mM; bovine gamma globulin, 100 mg/mL; sodium azide, 0.02%) were added into a 96-well plate containing the tested compounds at pH 7.5. The plate was incubated at 37 °C for 30 min and the polarization values were measured (485 nm excitation, 528 nm static, and polarized filter). The *K_i_* values were calculated according to a reported method [41].

### 3.6. MTT Assay in Vitro

All compounds with *K_i_* values of <1 μM toward MDM2 in the fluorescence polarization assay were evaluated against the MCF7, HCT116, and A549 cell lines (ATCC, Manassas, VA, USA) in vitro using a standard MTT assay, with nutlin-3a as a positive control. Compounds were tested at six concentrations (0.001–100 μM). 

The cells were seeded into a 96-well plate and allowed to attach overnight, after which various concentrations of test compounds or DMSO were added to the culture medium and the cells were incubated for 48 h at 37 °C in a 5% CO_2_ humidified incubator. Then, 20 μL of 0.5% the fresh MTT solution was added to each well. After incubation for another 4 h, the culture medium was removed and 150 μL of DMSO was added to dissolve the formazan crystals. The absorbance at 540 nm was measured using a microplate reader. The results, expressed as IC_50_ values, were the average of three determinations and were calculated using nonlinear regression analysis.

### 3.7. Western Blotting Assay

The HCT116 cells with wild type p53 were treated with compound **A13** (0, 5, 10, and 20 μM) for 48 h. The cells were harvested and lysed with RIPA buffer (Sigma, St. Louis, MI, USA) supplemented with a protease inhibitor cocktail (Sigma), followed by centrifugation at 12,000× *g* for 10 min. The supernatants were heated to 100 °C with loading buffer and the total protein concentrations were determined using a Bio-Rad protein assay. Protein samples were subjected to SDS-PAGE and transferred to a PVDF membrane. The membrane was blocked with 5% non-fat milk, and then incubated with the corresponding primary antibody (MDM2, MDMX, p53, and GAPDH) at 4 °C overnight. After binding with an appropriate secondary antibody conjugated with peroxidase, proteins were visualized using the ECL chemiluminescence system. GAPDH was used as a loading control.

### 3.8. Molecular Docking

Molecular docking analysis was carried out using Sybyl-X2.0. The crystal structures of MDM2 (PDB code: 3LBL) and MDMX (PDB code: 2N0W) were processed. The ligand and the water molecules were first removed and the hydrogen atoms were added. The biopolymer was protonated at pH 7.4 and the Amber7 FF99 force field was applied. Other parameters were set at the default. The minimized protein and compound **A13** were subjected to the docking protocol using the Surflex-Dock Geom mode of Sybyl-X2.0.

## 4. Conclusions

In this study, a library of new indolone derivatives was synthesized and evaluated for their biological activity. The preliminary structure–activity relationship study showed that the compounds with benzene sulfonyl groups demonstrated a higher potency than the benzene sulfonyl groups. The 3-substituted electron-withdrawing group at the R_3_ position is necessary (3-Cl > 3-F > 3-Br > 3-CF_3_ > 3-NO_2_) to increase the affinity to the enzyme. All of the compounds exhibited better activities to MDM2 than MDMX. Compound **A13** was identified as having the most potent affinity toward MDM2 and MDMX through the fluorescence polarization assay, with a *K_i_* of 0.031 and 7.24 μM, respectively. The docking studies demonstrated that compound **A13** formed hydrophobic interactions with Leu54, Tyr67, His73, Val93, His96, Ile99, and Tyr100 in the MDM2 active site, and Met53, Ile60, Met61, Tyr66, and Val92 in the MDMX active site. **A13** also exhibited a greater potency than nutlin-3a against the HCT116, MCF7, and 143B cell lines. The Western blot analysis showed that **A13** induced accumulations of MDM2, MDMX, and p53, suggesting the activation of the p53 pathway.

## Figures and Tables

**Table 1:** The MDM2 and MDMX binding affinity of the synthesized compounds.

Compd.	R_1_	R_2_	R_3_	K_i_ (μM) ± SD	
				MDM2	MDMX
**A1**	H	2-Cl	H	4.39 ± 0.82	>50
**A2**	H	2-Cl	2-CH_3_	7.23 ± 1.03	>50
**A3**	H	2-Cl	3-CH_3_	5.58 ± 0.99	>50
**A4**	H	2-Cl	4-CH_3_	10.16 ± 1.45	>50
**A5**	H	2-Cl	2-Cl	3.43 ± 0.57	42.1 ± 6.35
**A6**	H	2-Cl	3-Cl	0.92 ± 0.11	17.43 ± 2.78
**A7**	H	2-Cl	4-Cl	1.53 ± 0.19	20.38 ± 2.62
**A8**	H	3-Cl	H	1.47 ± 0.15	10.33 ± 1.45
**A9**	H	3-Cl	2-CH_3_	6.95 ± 0.97	>50
**A10**	H	3-Cl	3-CH_3_	5.36 ± 1.06	>50
**A11**	H	3-Cl	4-CH_3_	5.19 ± 1.25	>50
**A12**	H	3-Cl	2-Cl	0.19 ± 0.05	20.83 ± 2.01
**A13**	H	3-Cl	3-Cl	0.031 ± 0.007	7.24 ± 0.78
**A14**	H	3-Cl	4-Cl	0.082 ± 0.021	18.19 ± 1.39
**A15**	H	3-Cl	2-Br	0.34 ± 0.12	24.37 ± 2.57
**A16**	H	3-Cl	3-Br	0.079 ± 0.005	16.21 ± 1.94
**A17**	H	3-Cl	4-Br	0.49 ± 0.15	43.18 ± 4.95
**A18**	H	3-Cl	2-F	0.17 ± 0.03	17.69 ± 2.16
**A19**	H	3-Cl	3-F	0.076 ± 0.005	16.89 ± 1.74
**A20**	H	3-Cl	4-F	0.23 ± 0.02	29.16 ± 3.51
**A21**	H	3-Cl	3-CF_3_	0.092 ± 0.007	19.26 ± 2.69
**A22**	H	3-Cl	3-NO_2_	1.39 ± 0.21	13.57 ± 1.47
**A23**	H	3-Cl	4-OCH_3_	3.77 ± 0.45	42.17 ± 4.73
**A24**	H	4-Cl	2-CH_3_	3.17 ± 0.37	34.69 ± 4.02
**A25**	H	4-Cl	3-CH_3_	4.93 ± 0.47	>50
**A26**	H	4-Cl	4-CH_3_	4.68 ± 0.39	>50
**A27**	H	4-Cl	3-Cl	0.31 ± 0.03	17.26 ± 1.91
**A28**	H	4-Cl	H	2.18 ± 0.29	37.43 ± 3.57
**A29**	5-Cl	3-Cl	3-Cl	0.28 ± 0.04	32.24 ± 3.24
**A30**	6-Cl	3-Cl	3-Cl	1.39 ± 0.15	28.96 ± 2.18
**B1**	H	2-Cl	2-Cl	10.17 ± 2.01	>50
**B2**	H	2-Cl	3-Cl	8.43 ± 1.58	>50
**B3**	H	2-Cl	4-Cl	7.86 ± 1.34	>50
**B4**	H	2-Cl	2-CH_3_	16.35 ± 2.77	>50
**B5**	H	2-Cl	3-CH_3_	20.19 ± 3.05	>50
**B6**	H	2-Cl	4-CH_3_	22.37 ± 3.64	>50
**B7**	H	3-Cl	2-Cl	14.87 ± 2.37	>50
**B8**	H	3-Cl	3-Cl	8.63 ± 1.55	>50
**B9**	H	3-Cl	4-Cl	15.92 ± 1.96	>50
**B10**	H	4-Cl	3-Cl	12.49 ± 1.04	>50
**Nutlin-3a**	-	-	-	0.15 ± 0.02	>50

**Table 2:** The anti-tumor activities of several compounds against the HCT116, MCF7, and 143B cell lines in vitro.

Compd.	IC_50_ (μM)		
	HCT116	MCF7	A549
**A6**	10.72 ± 1.47	25.47 ± 3.61	18.72 ± 2.49
**A12**	15.36 ± 4.61	24.72 ± 2.53	17.83 ± 2.19
**A13**	6.17 ± 0.93	11.21 ± 1.95	12.49 ± 2.01
**A14**	15.18 ± 3.14	20.19 ± 2.86	20.33 ± 2.49
**A15**	23.79 ± 2.69	31.08 ± 3.55	29.12 ± 3.31
**A16**	21.73 ± 2.59	26.49 ± 3.40	25.44 ± 3.62
**A17**	25.47 ± 3.26	37.70 ± 3.65	34.17 ± 3.39
**A18**	17.88 ± 2.11	24.74 ± 3.63	22.58 ± 2.93
**A19**	10.15 ± 1.37	21.03 ± 2.58	16.27 ± 1.82
**A20**	16.32 ± 2.73	27.18 ± 2.83	22.19 ± 2.13
**A21**	8.91 ± 1.35	15.69 ± 2.04	13.48 ± 2.73
**A27**	17.49 ± 1.53	27.62 ± 3.81	20.36 ± 1.98
**A29**	16.96 ± 2.37	27.43 ± 3.37	26.35 ± 4.05
**Nutlin-3a**	18.13 ± 2.17	32.18 ± 1.49	21.72 ± 2.04

## Data Availability

The data presented in this study are available on request from the corresponding author.

## Supplementary Materials

The following supporting information can be downloaded at: https://www.mdpi.com/article/10.3390/molecules27123721/s1, the ^1^H NMR spectrum, ^13^C NMR spectrum, and mass spectrum of compounds **1–5**, **A1–A30**, and **B1–B10**.

## Sample Availability

Samples of the compounds are available from the authors.
