# Supplementary material for: Synthesis and Biological Evaluation of Novel Synthetic Indolone Derivatives as Anti-Tumor Agents Targeting p53-MDM2 and p53-MDMX
Source: Molecules. 2022 Jun 9;27(12):3721. doi: 10.3390/molecules27123721 (PMC9230548; doi:10.3390/molecules27123721)
Supplement: Supplementary file 1 [file molecules-27-03721-s001.zip › molecules-1750253-supplementary.pdf]

molecules

MDPI

Supplementary Matterials

Synthesis and Biological Evaluation of Novel Synthetic Indolone Derivatives as Anti-Tumor Agents Targeting p53-MDM2 and p53-MDMX

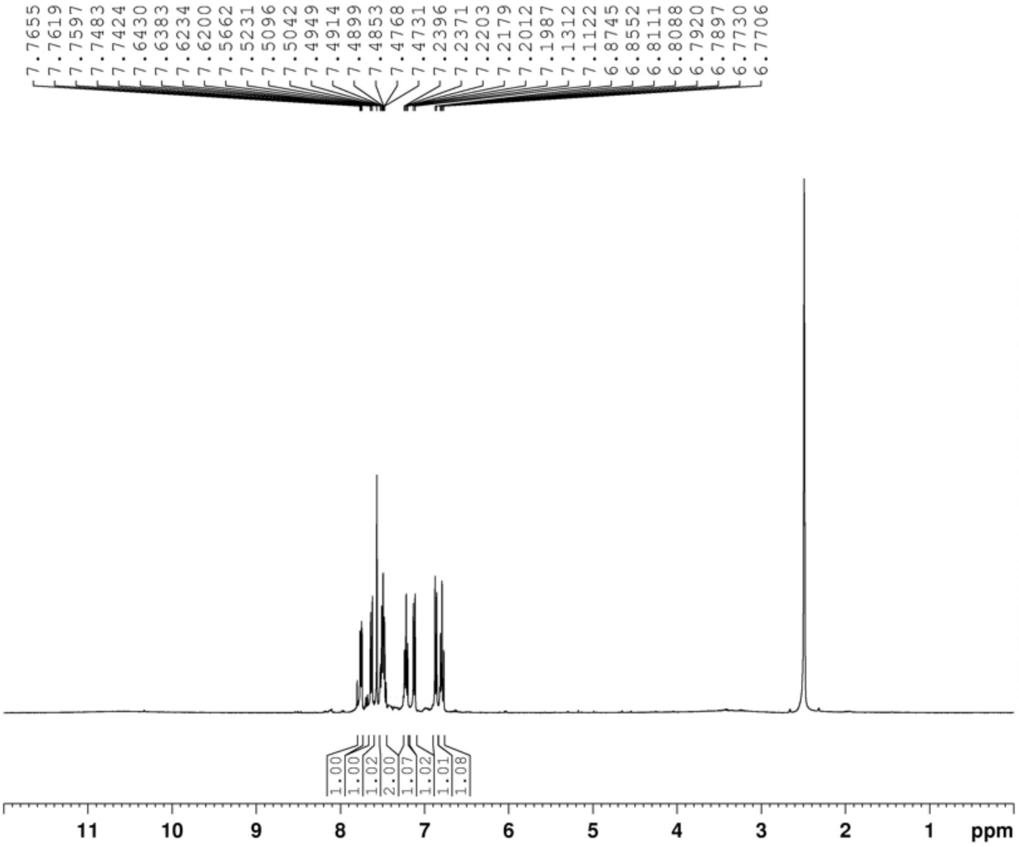

Figure S1. ^{1}H NMR spectrum of compound 1 (400 MHz, DMSO).

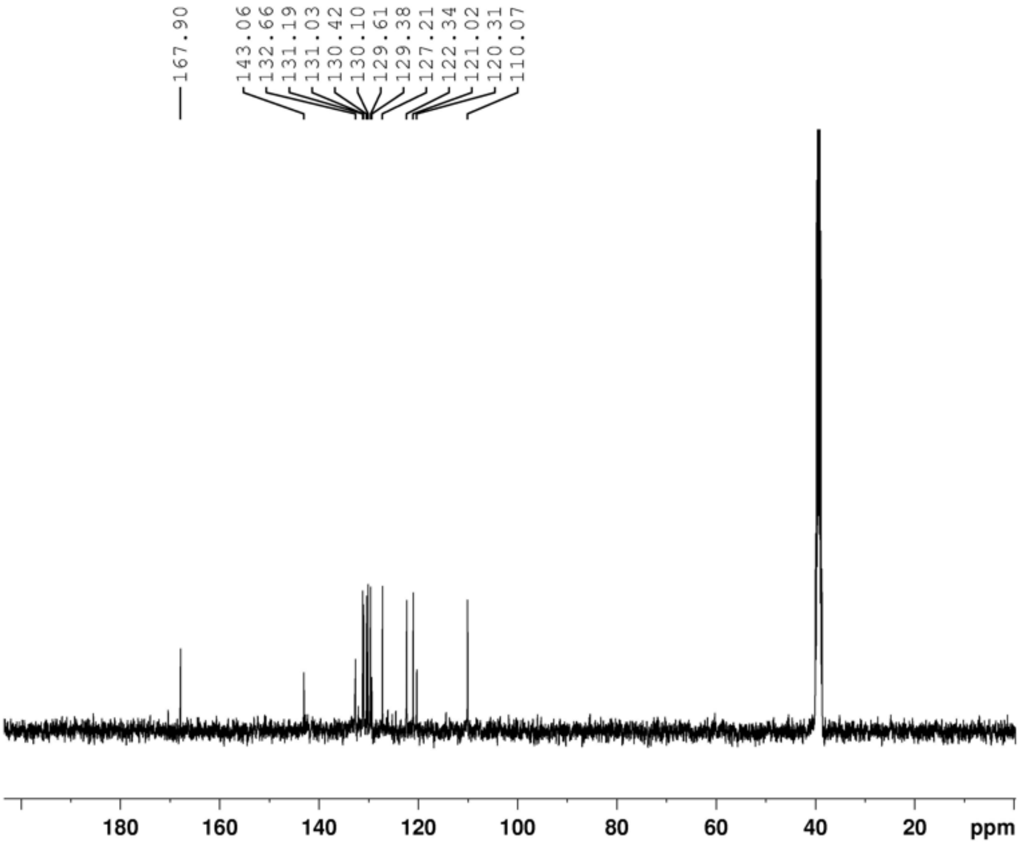

Figure S2. ^{13}C NMR spectrum of compound 1 (101 MHz, DMSO).

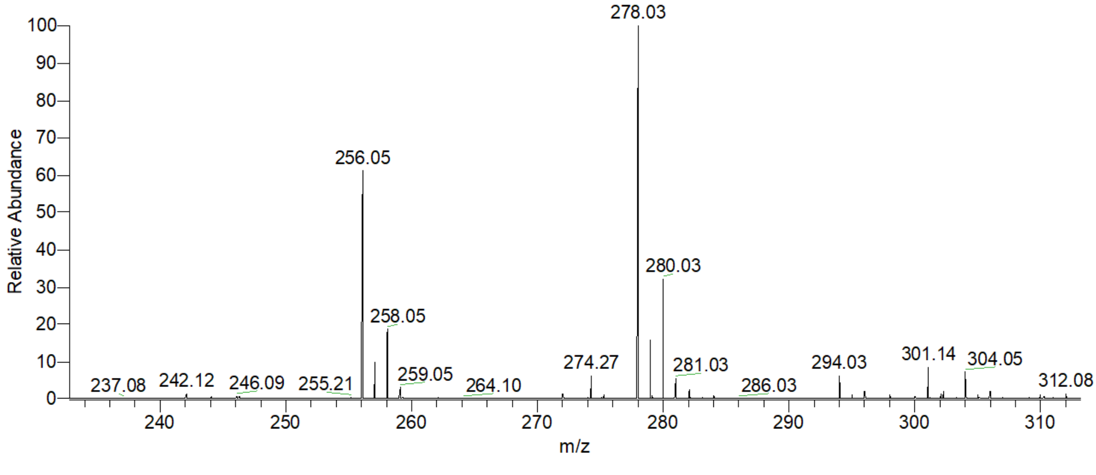

Figure S3. High resolution mass spectrum of compound 1.

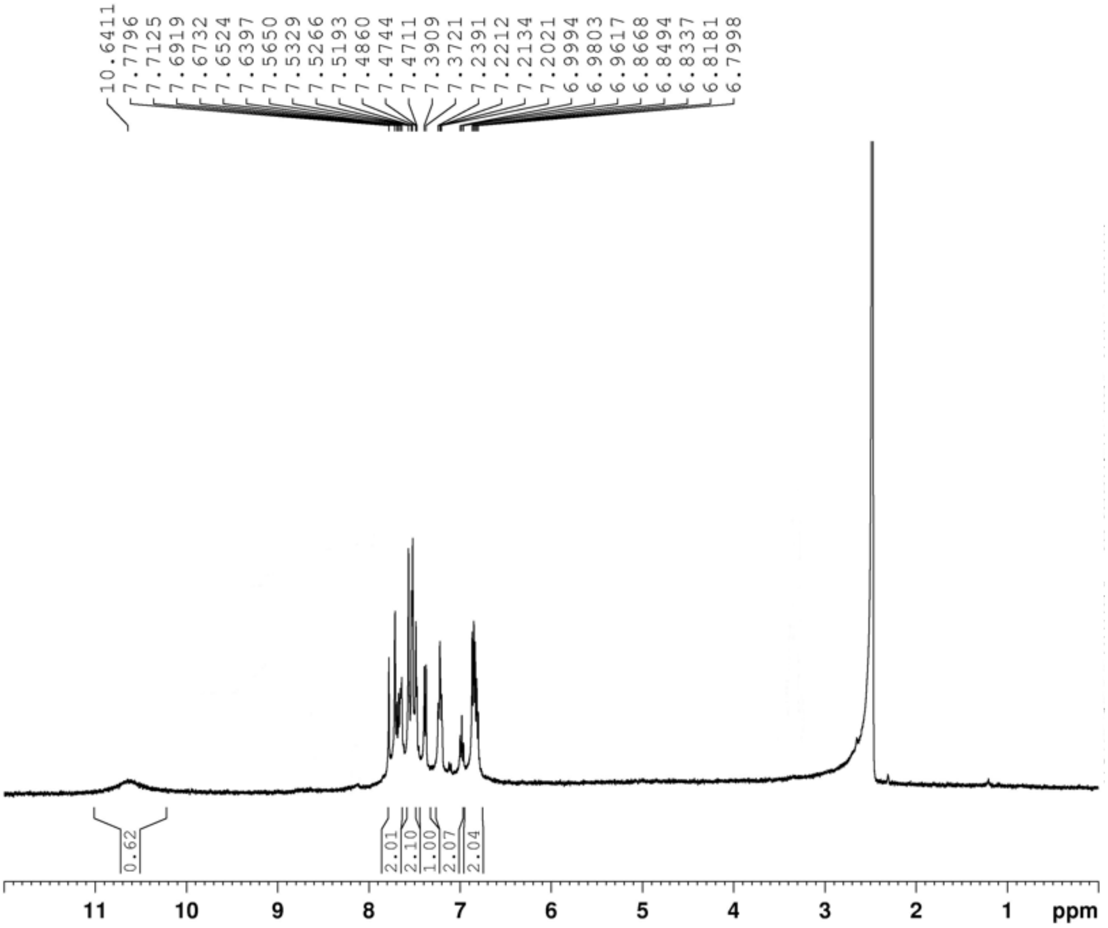

Figure S4: ^{1}H NMR spectrum of compound 2 (400 MHz, DMSO).

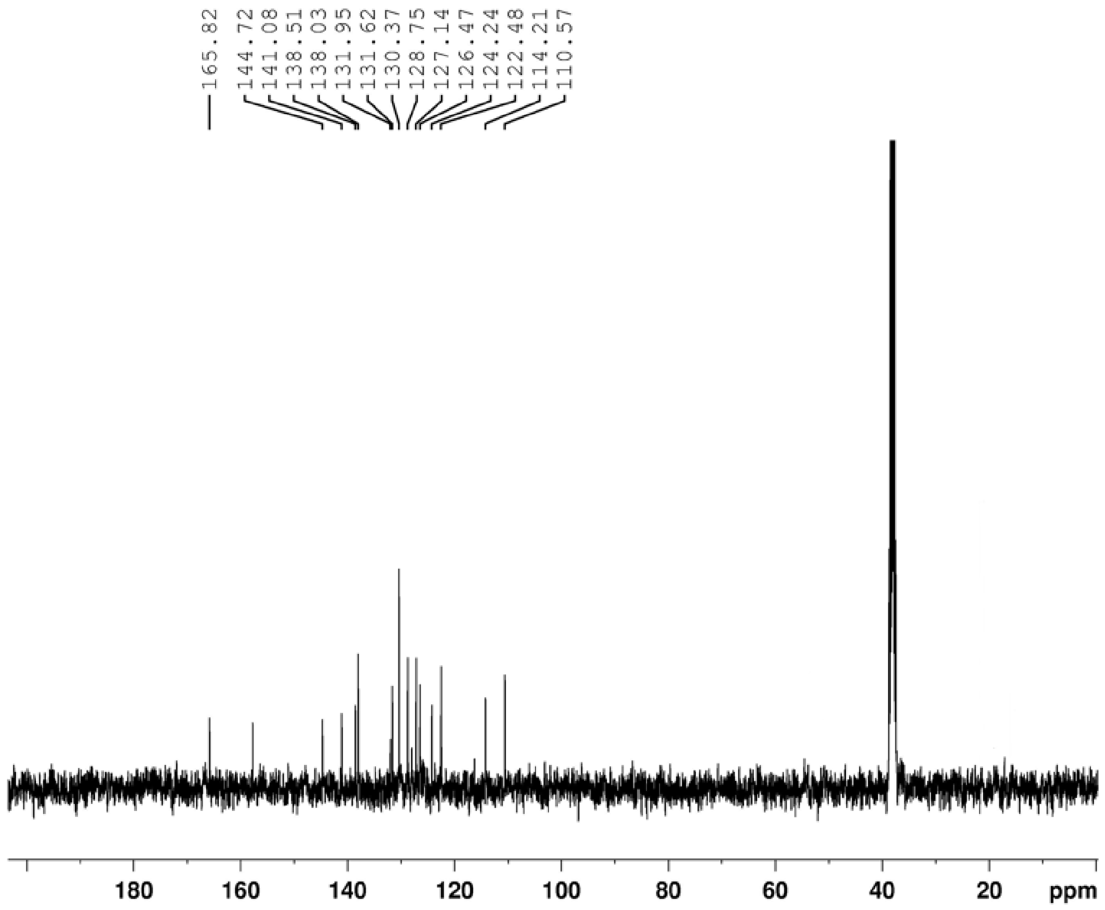

Figure S5. ^{13}C NMR spectrum of compound 2 (101 MHz, DMSO).

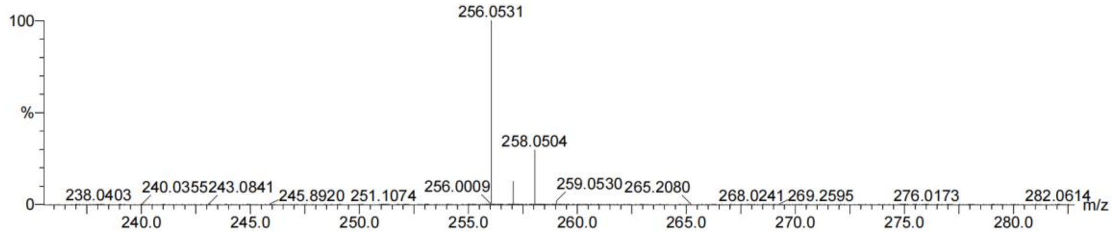

Figure S6. High resolution mass spectrum of compound 2.

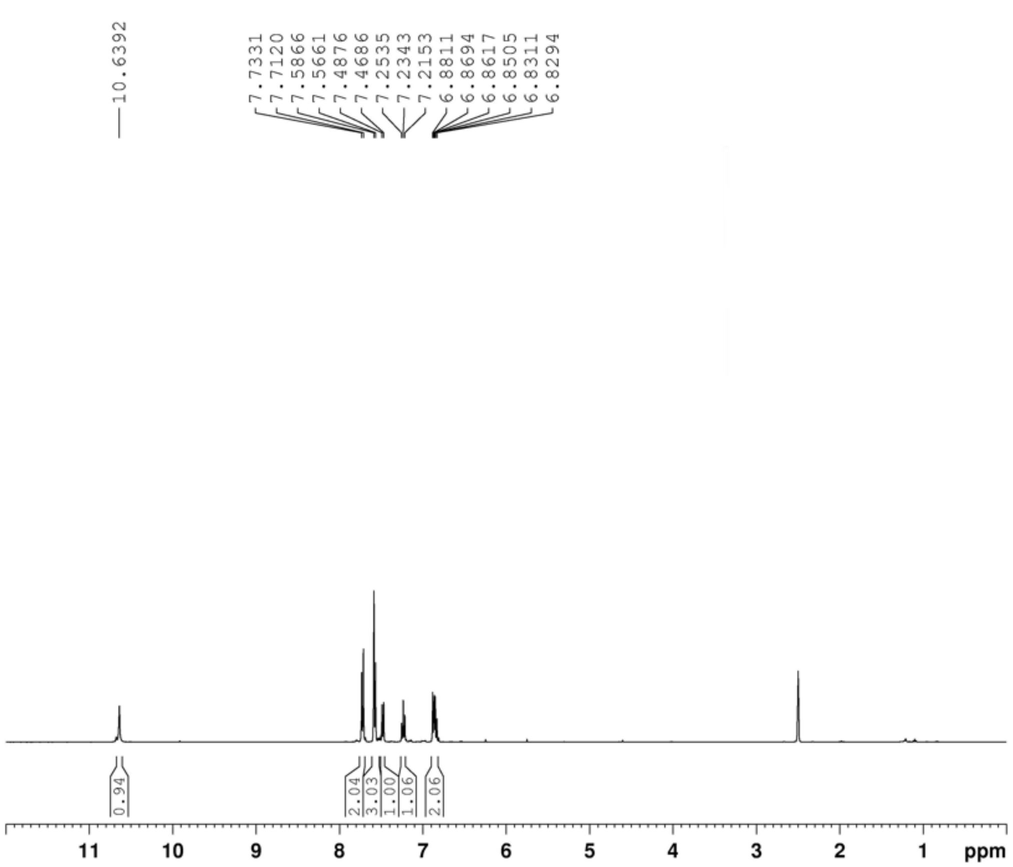

Figure S7. ^{1}H NMR spectrum of compound 3 (400 MHz, DMSO).

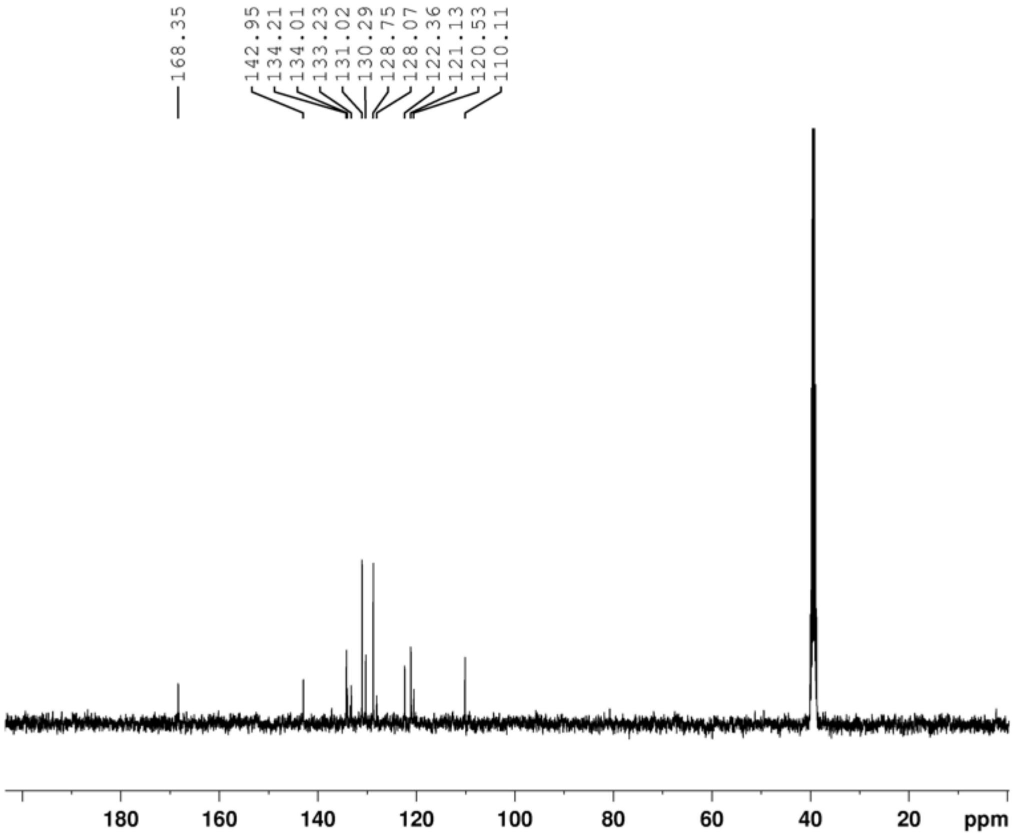

Figure S8. ^{13}C NMR spectrum of compound 3 (101 MHz, DMSO).

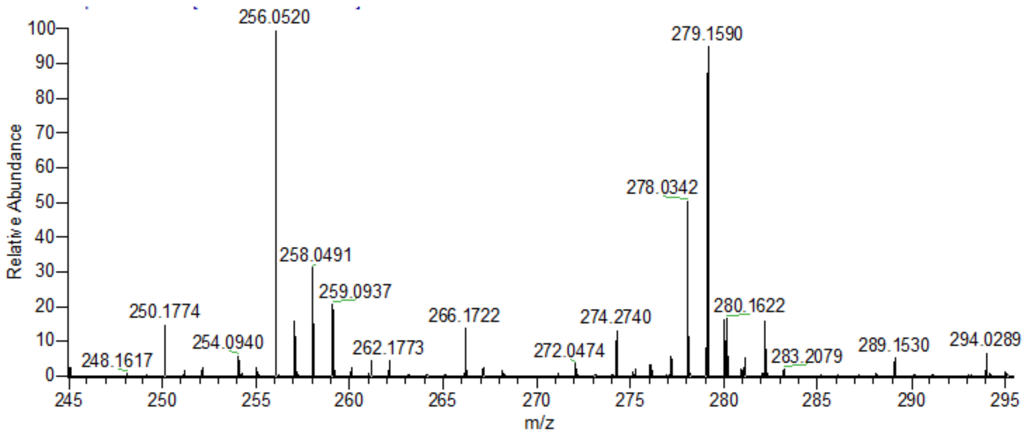

Figure S9. High resolution mass spectrum of compound 3.

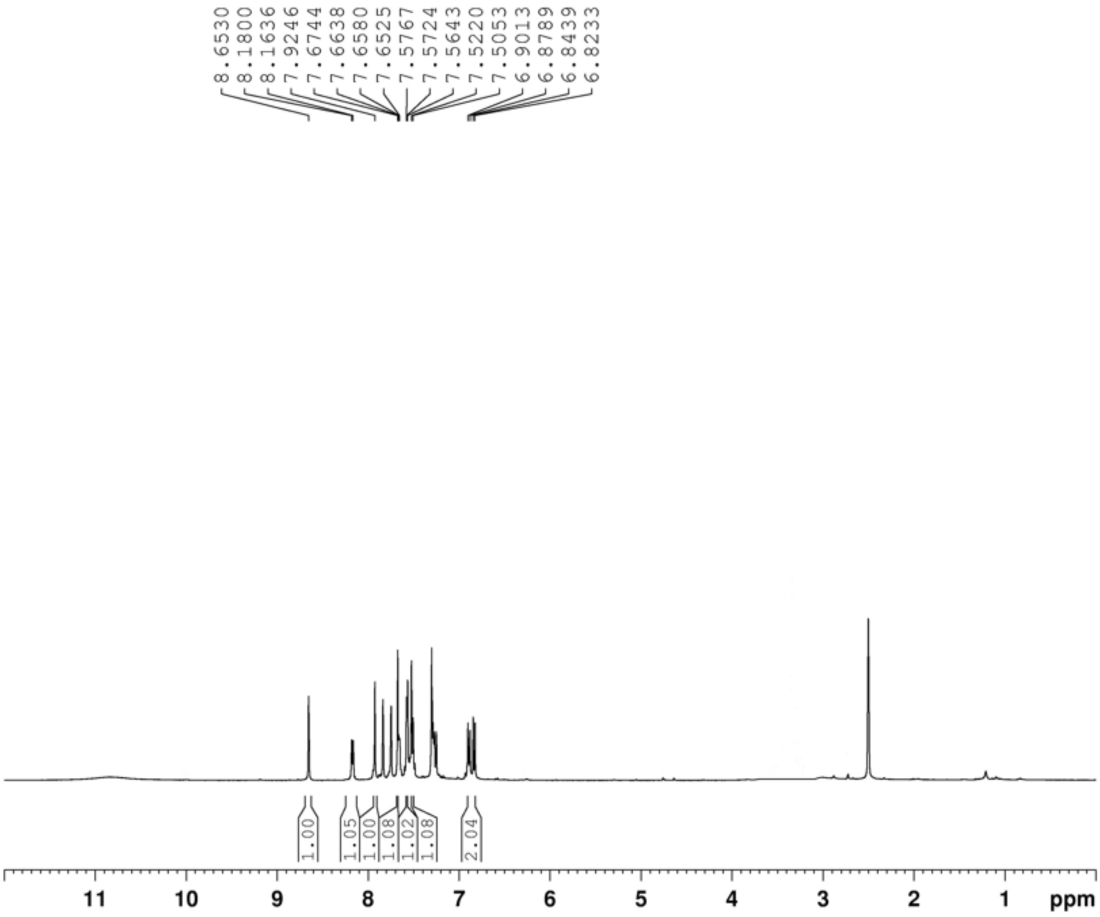

Figure S10. ^{1}H NMR spectrum of compound 4 (400 MHz, DMSO).

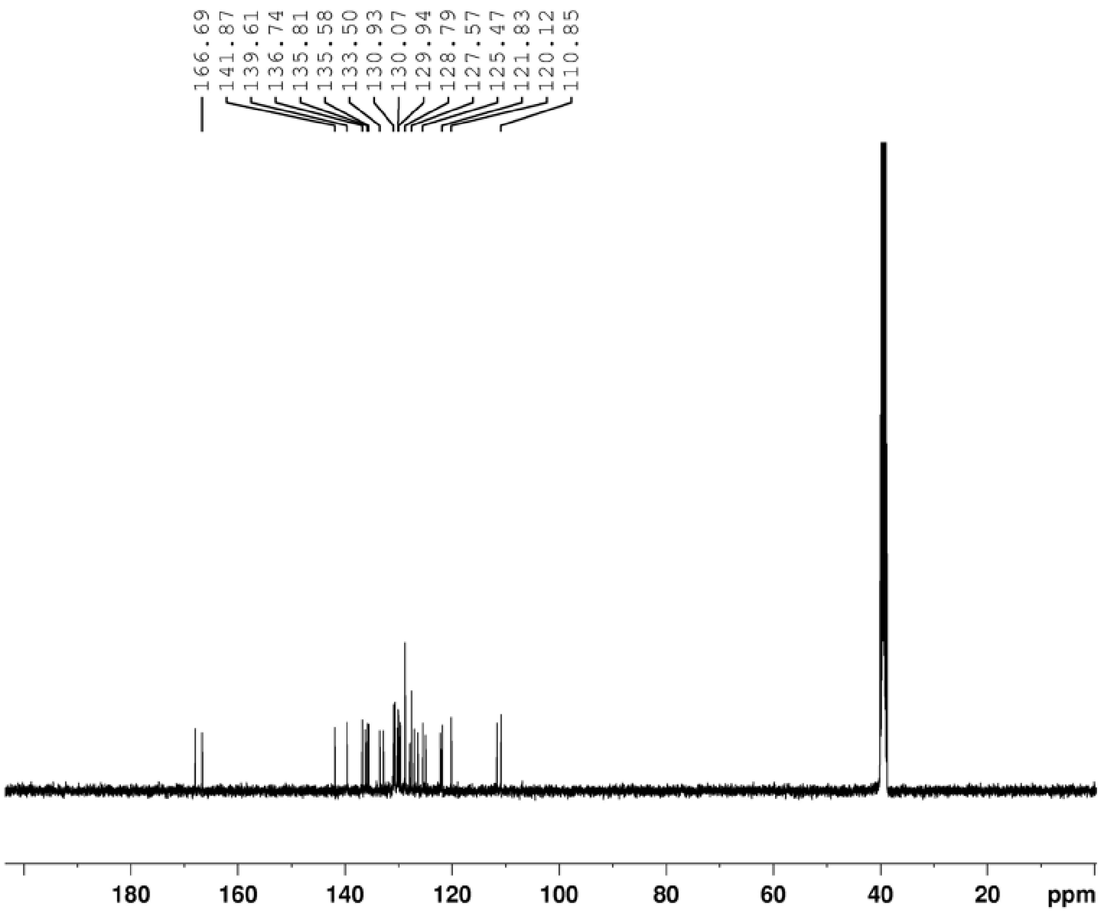

Figure S11. ^{13}C NMR spectrum of compound 4 (101 MHz, DMSO).

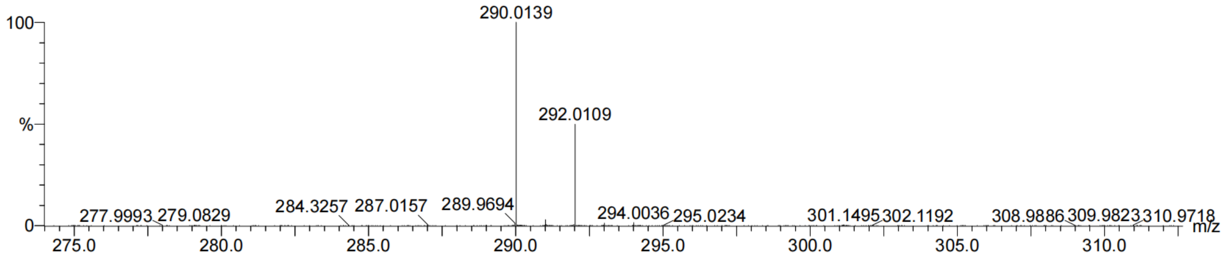

Figure S12. High resolution mass spectrum of compound 4.

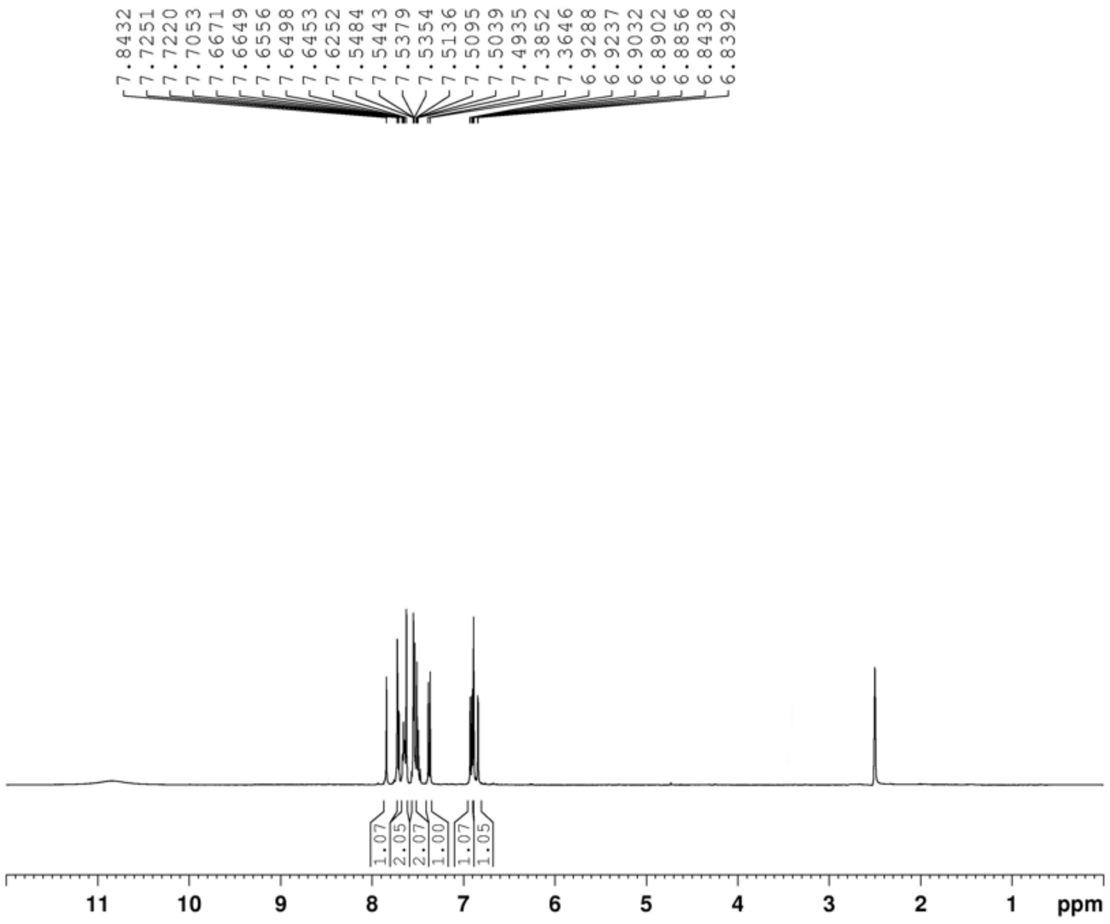

Figure S13. H NMR spectrum of compound 5 (400 MHz, DMSO).

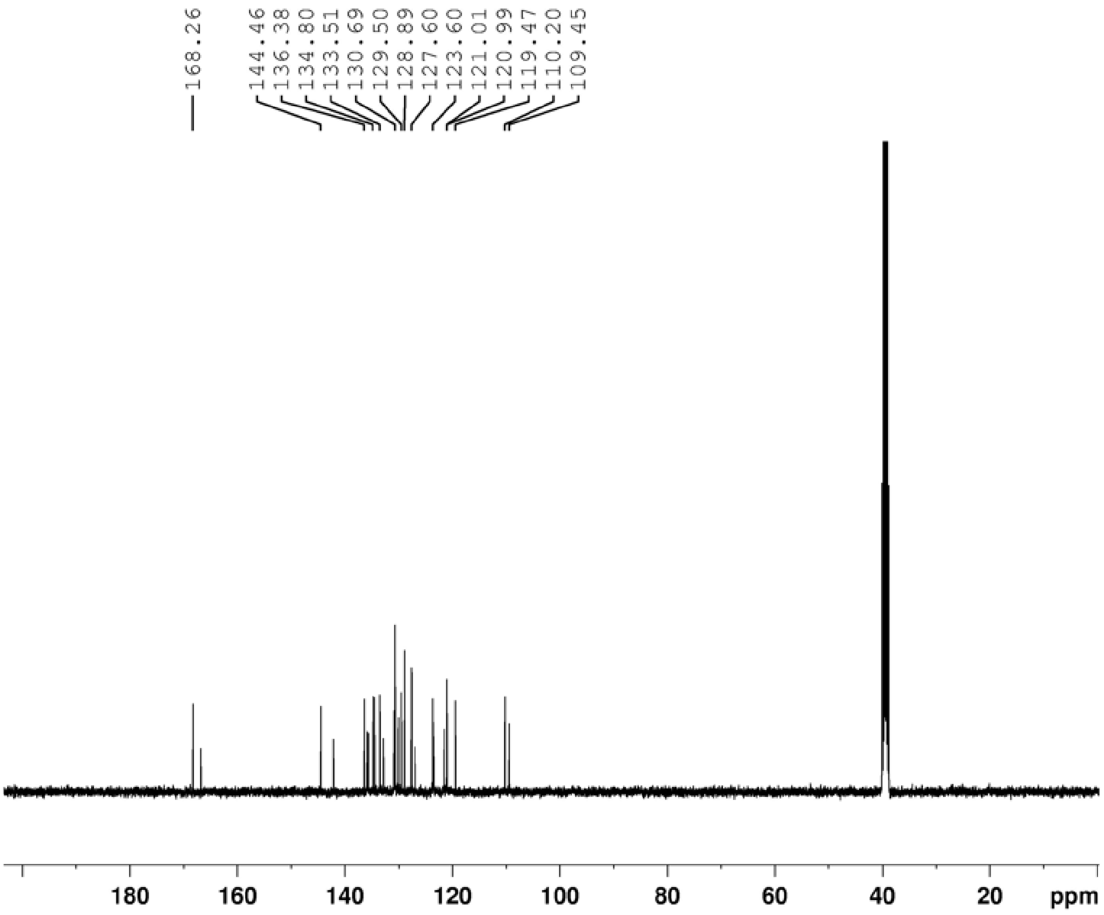

Figure S14. ^{13}C NMR spectrum of compound 5 (101 MHz, DMSO).

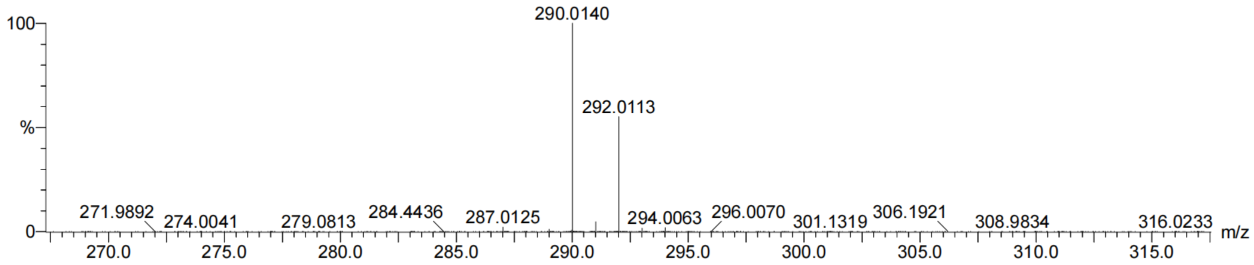

Figure S15. High resolution mass spectrum of compound 5.

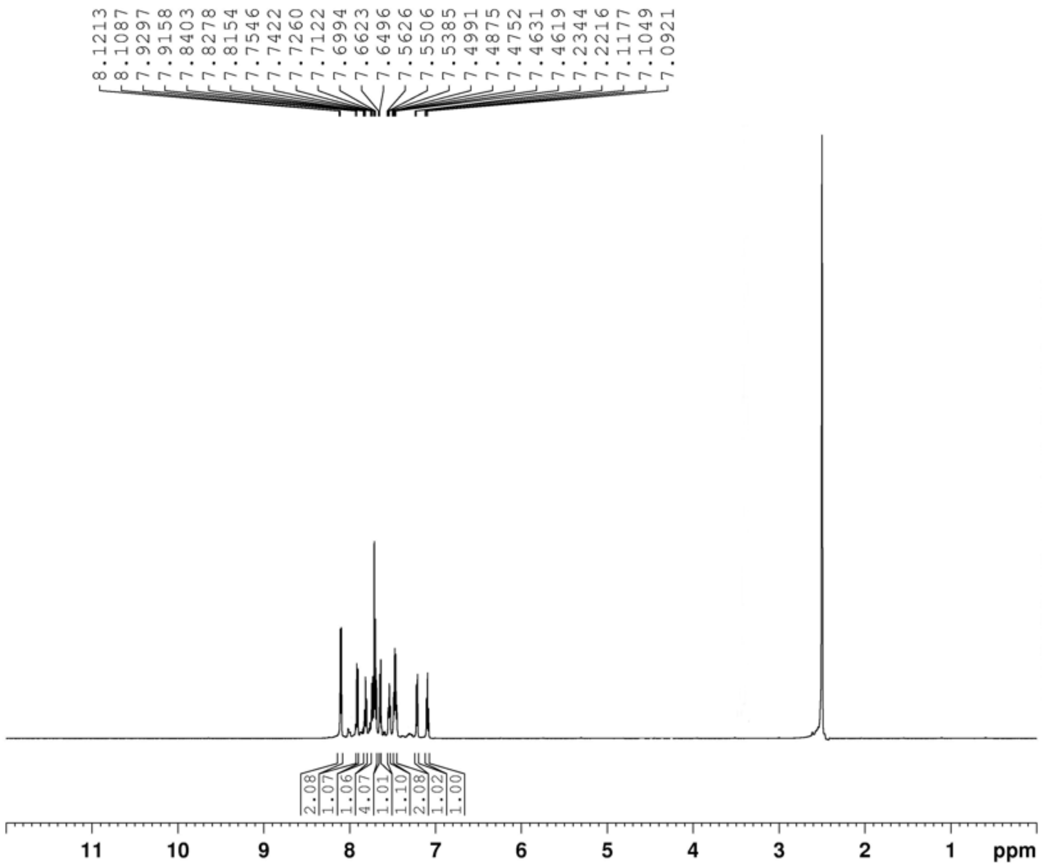

Figure S16. ^{1}H NMR spectrum of compound A1 (600 MHz, DMSO).

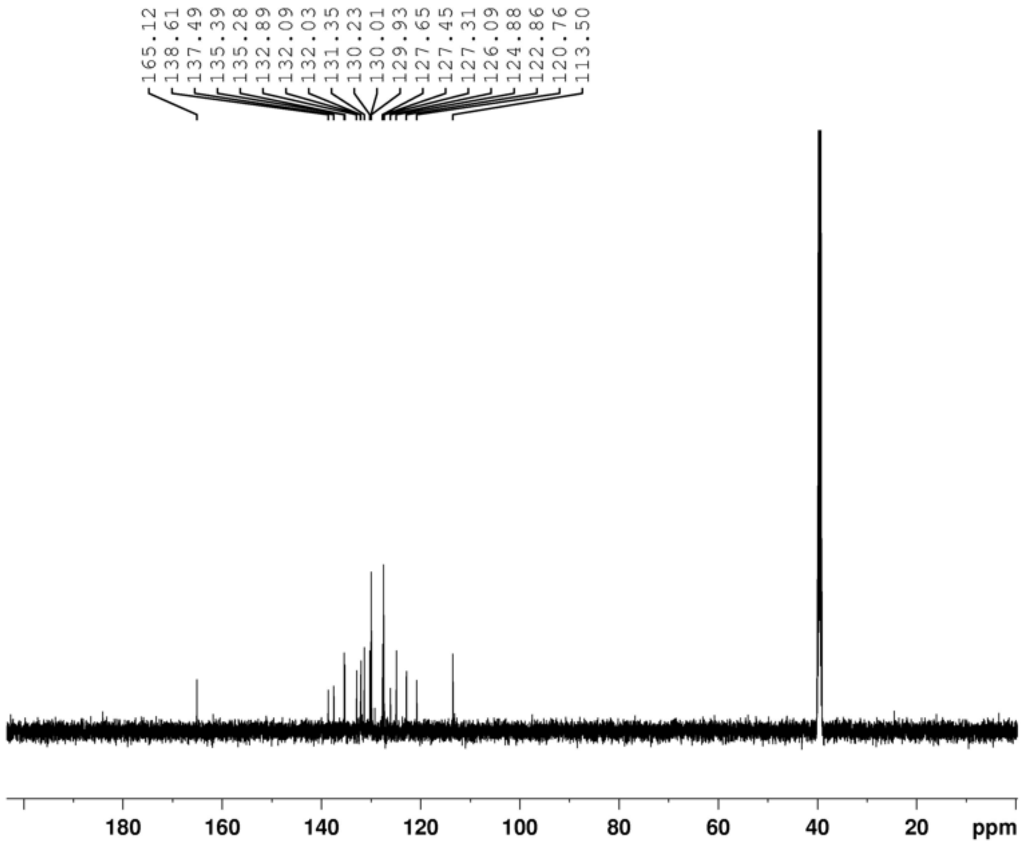

Figure S17. ^{13}C NMR spectrum of compound A1 (151 MHz, DMSO).

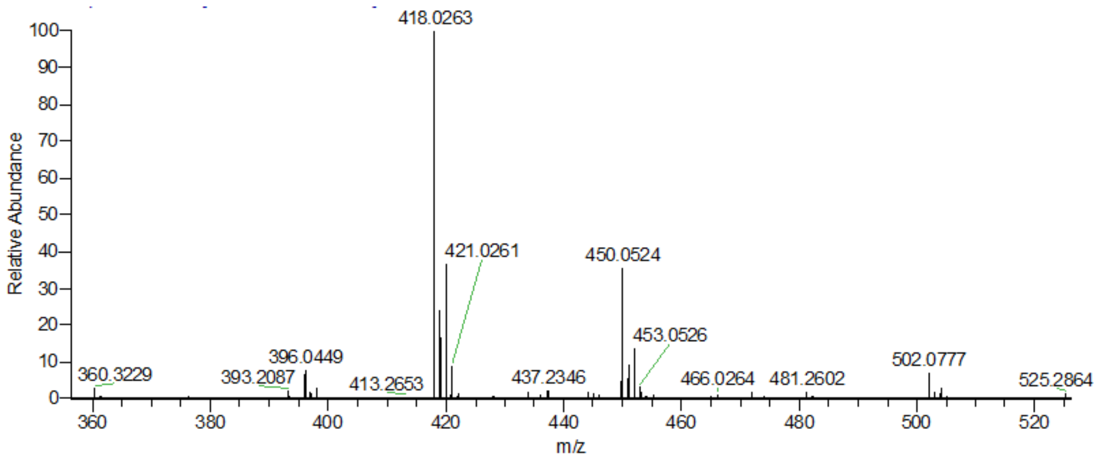

Figure S18. High resolution mass spectrum of compound A1.

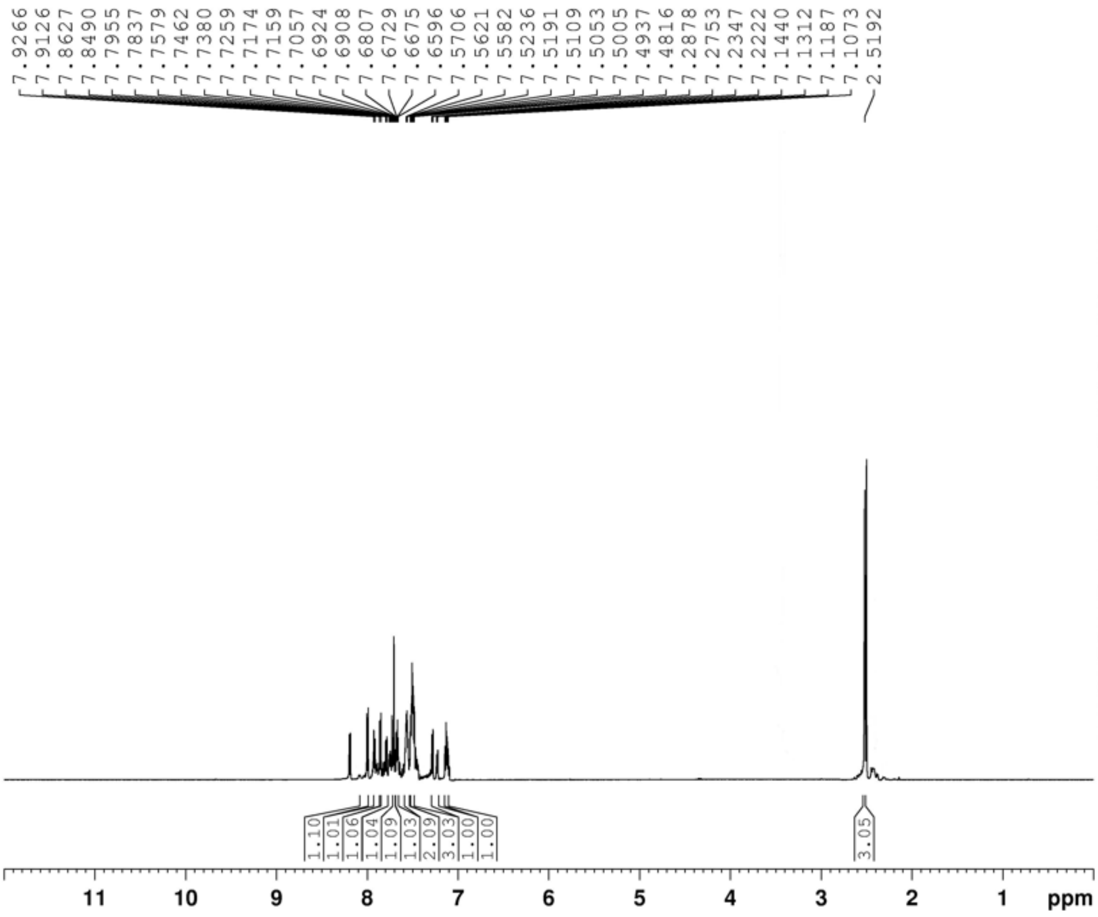

Figure S19. ^{1}H NMR spectrum of compound A2 (600 MHz, DMSO).

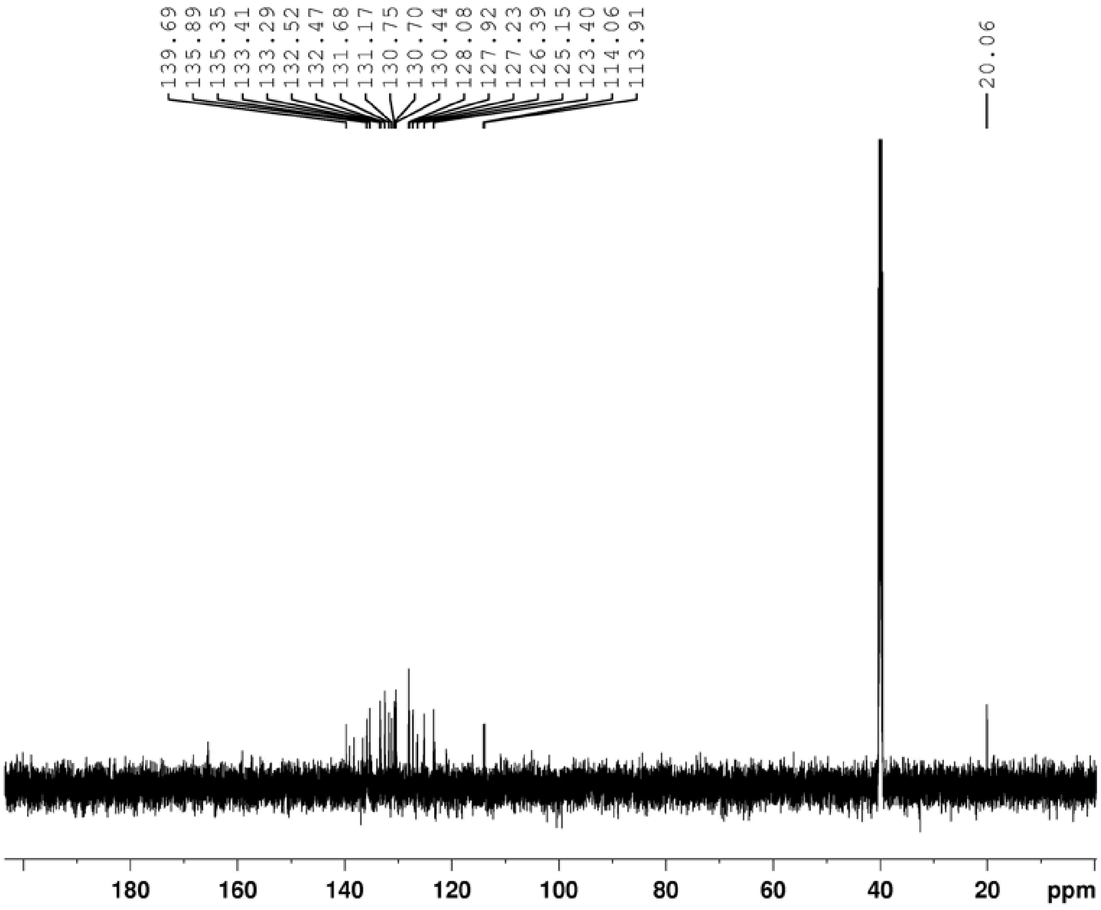

Figure S20. ^{13}C NMR spectrum of compound A2 (151 MHz, DMSO).

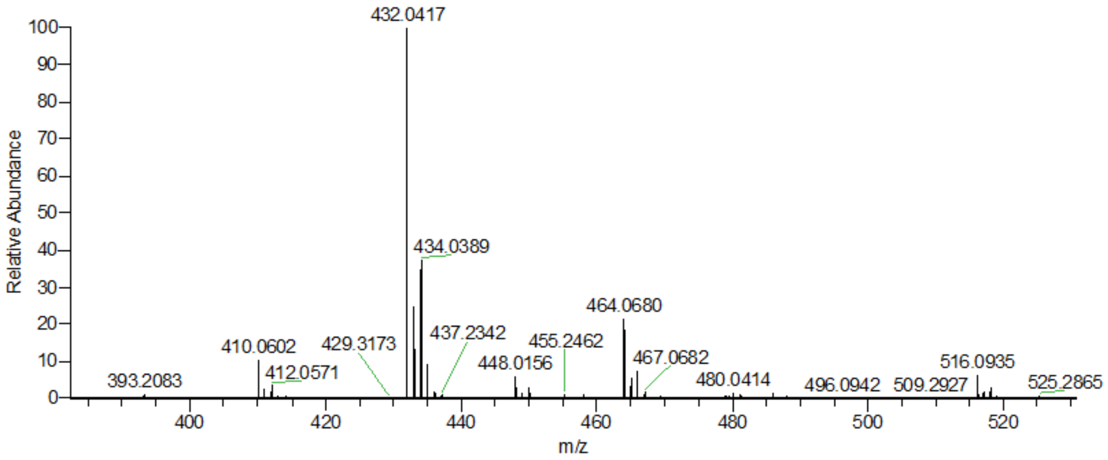

Figure S21. High resolution mass spectrum of compoundA2.

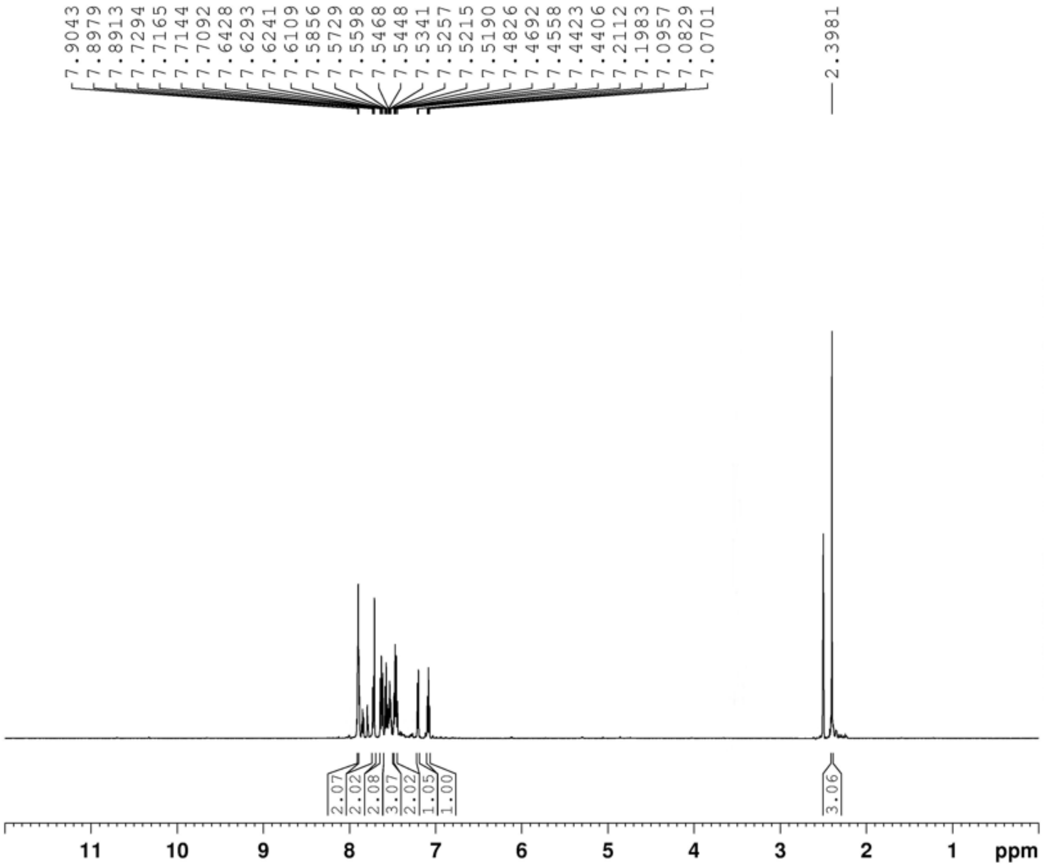

Figure S22. ^{1}H NMR spectrum of compound A3 (600 MHz, DMSO).

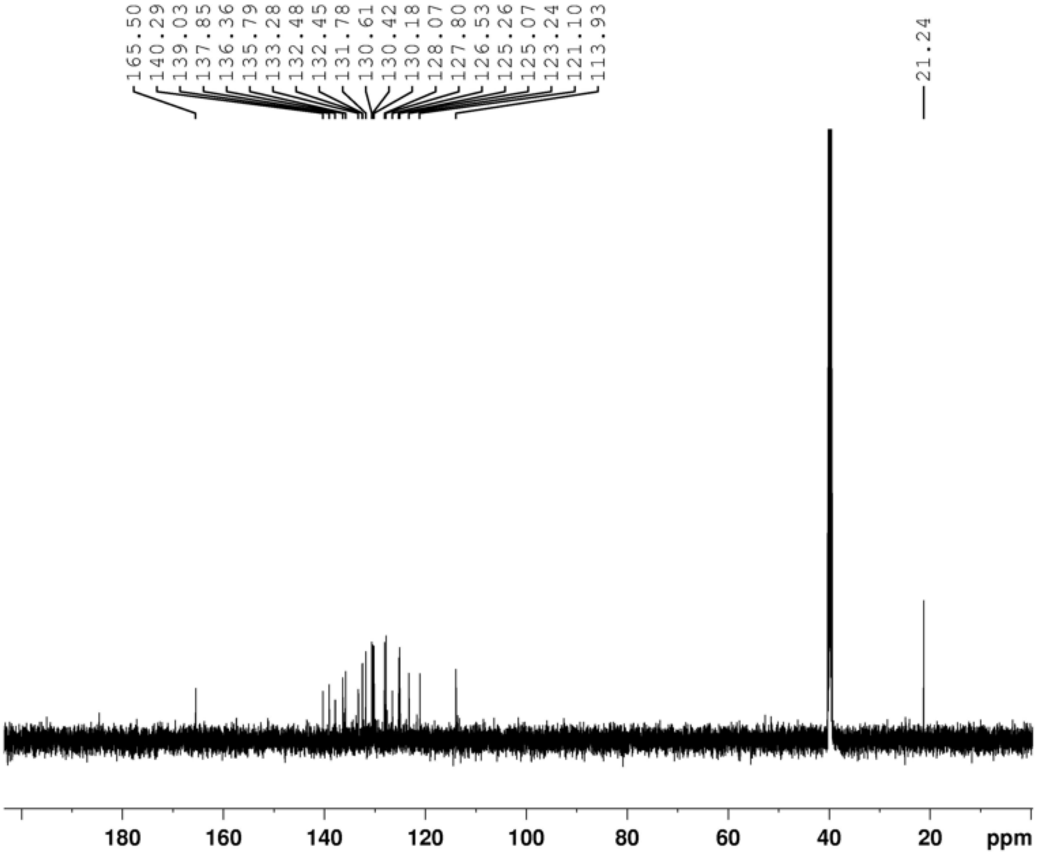

Figure S23. ^{13}C NMR spectrum of compound A3 (151 MHz, DMSO).

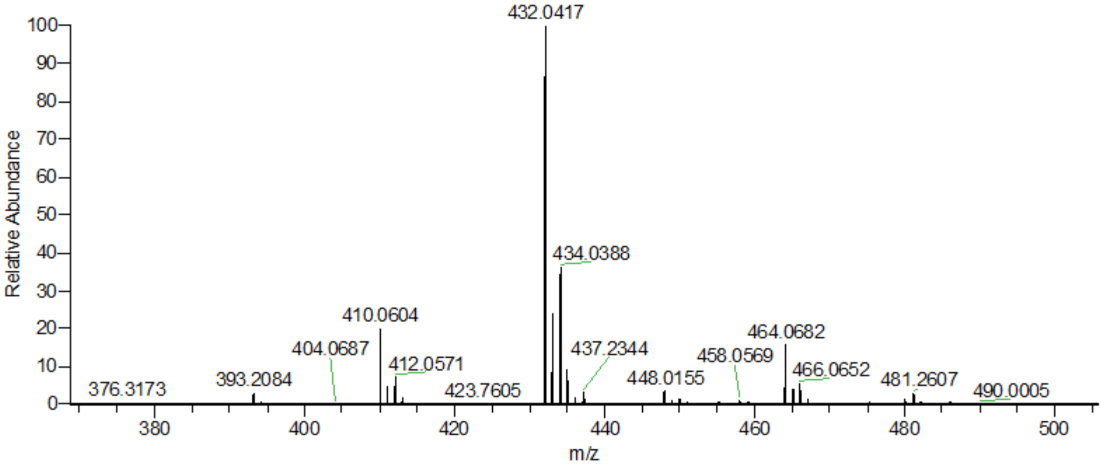

Figure S24. High resolution mass spectrum of compound A3.

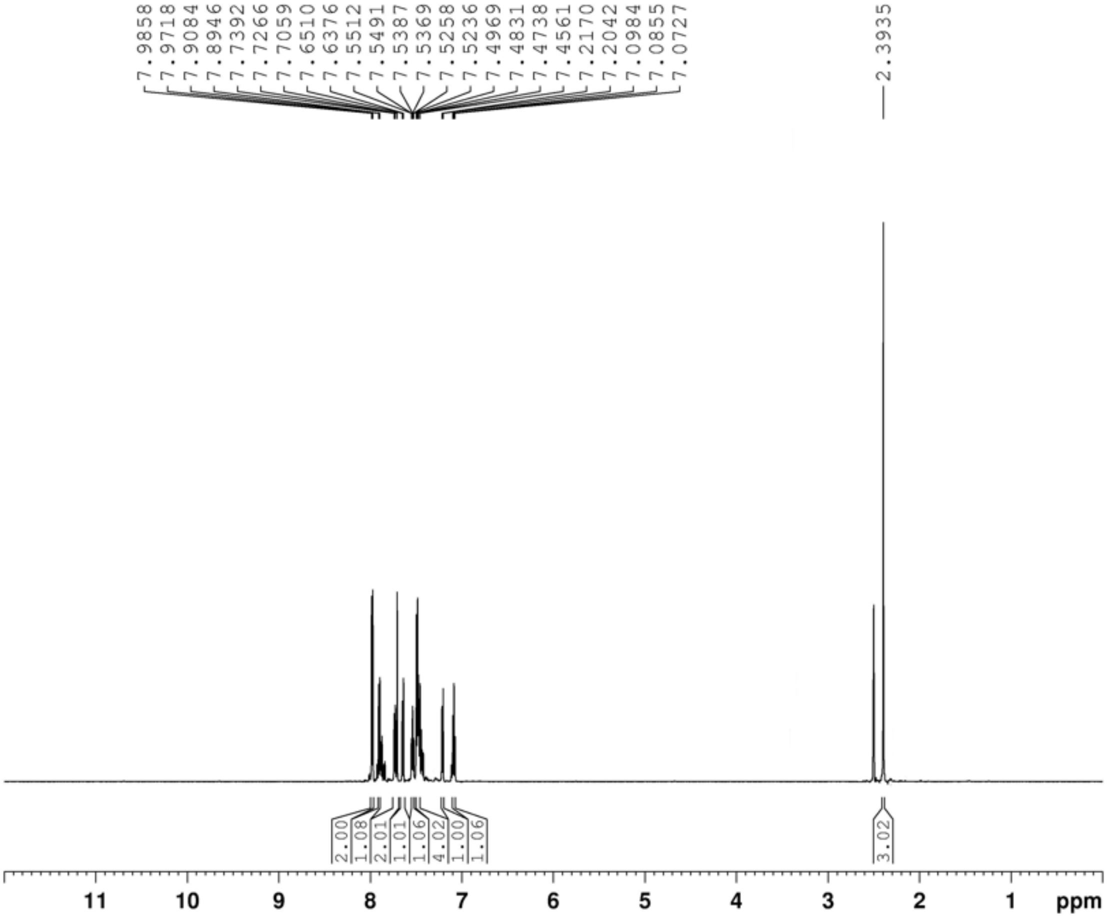

Figure S25. ^{1}H NMR spectrum of compound A4 (600 MHz, DMSO).

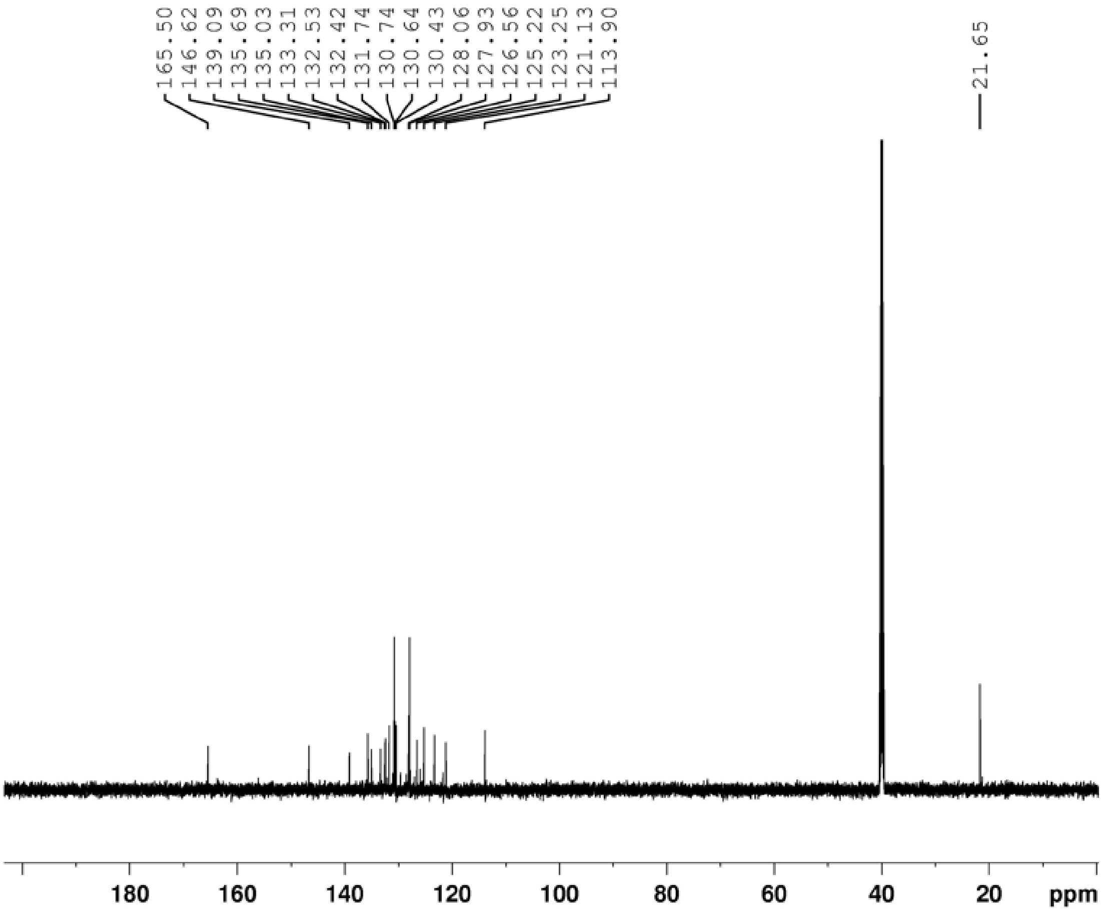

Figure S26. ^{13}C NMR spectrum of compound A4 (151 MHz, DMSO).

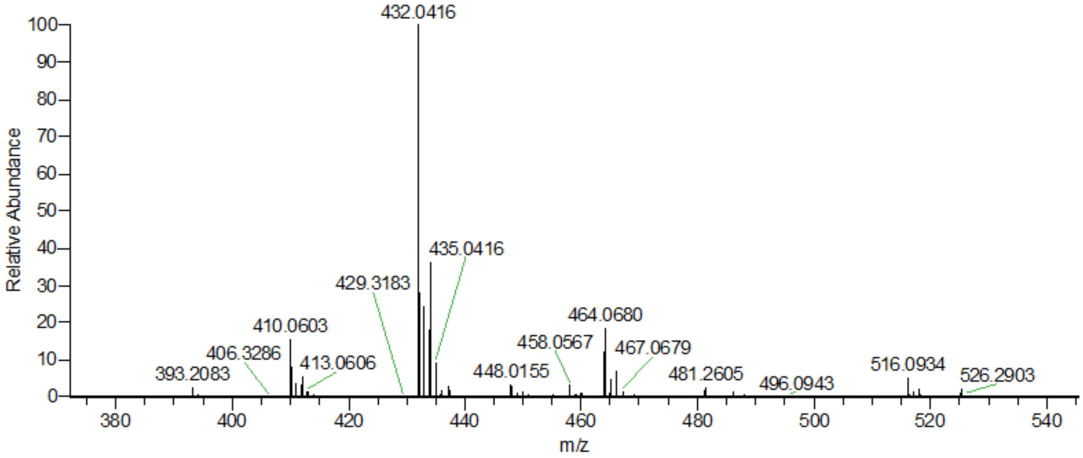

Figure S27. High resolution mass spectrum of compound A4.

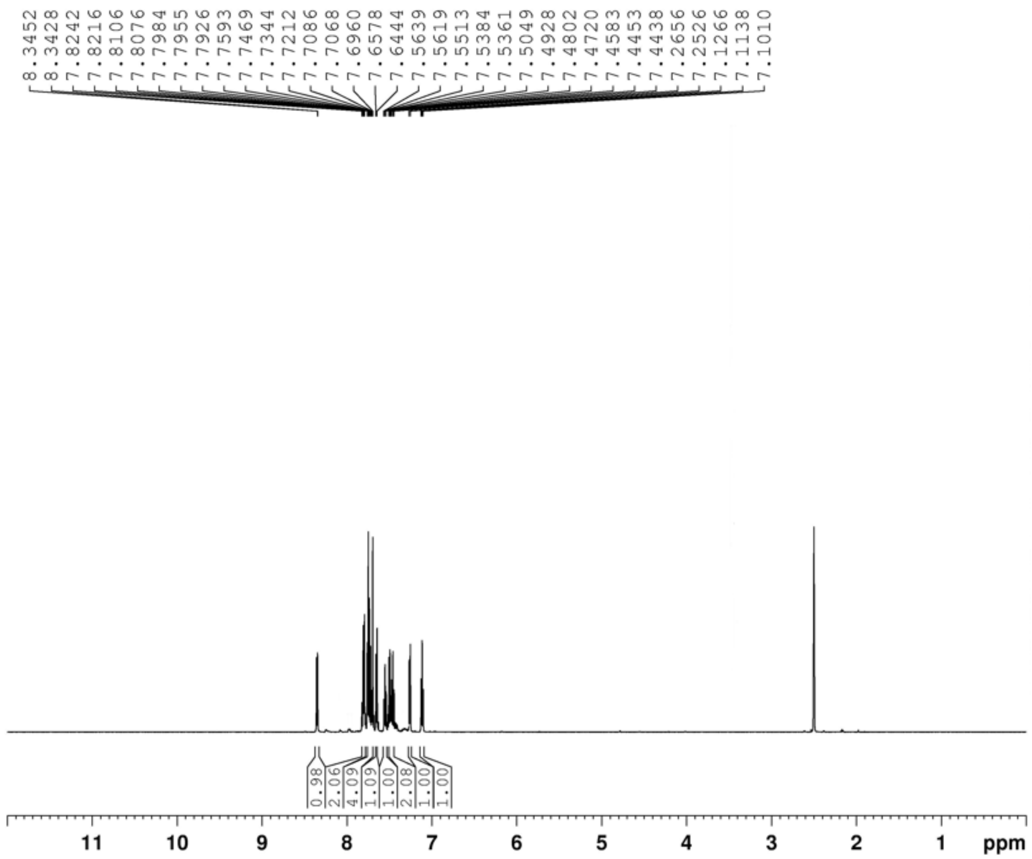

Figure S28. ^{1}H NMR spectrum of compound A5 (600 MHz, DMSO).

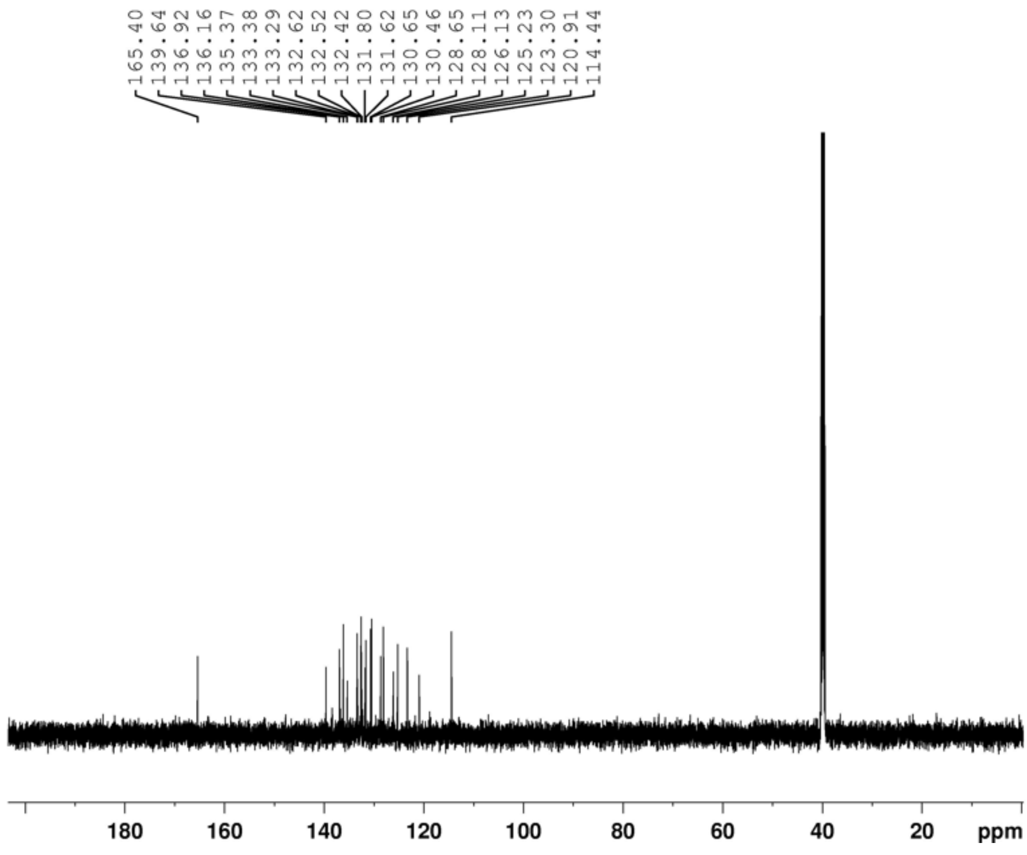

Figure S29. ^{13}C NMR spectrum of compound A5 (151 MHz, DMSO).

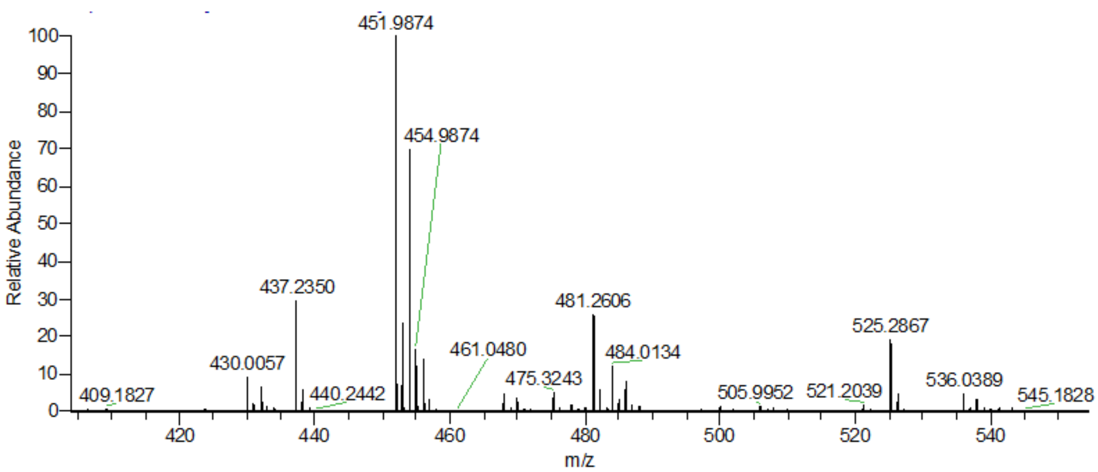

Figure S30. High resolution mass spectrum of compound A5.

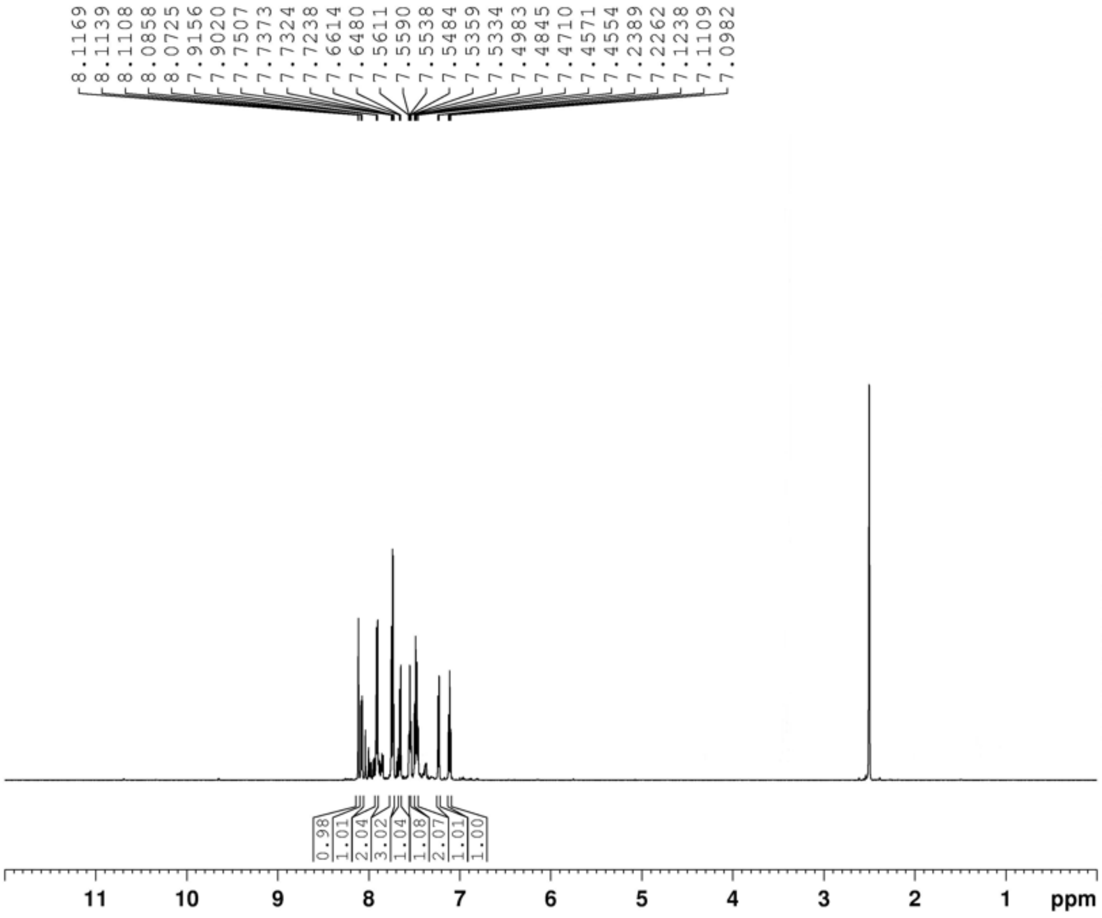

Figure S31. H NMR spectrum of compound A6 (600 MHz, DMSO).

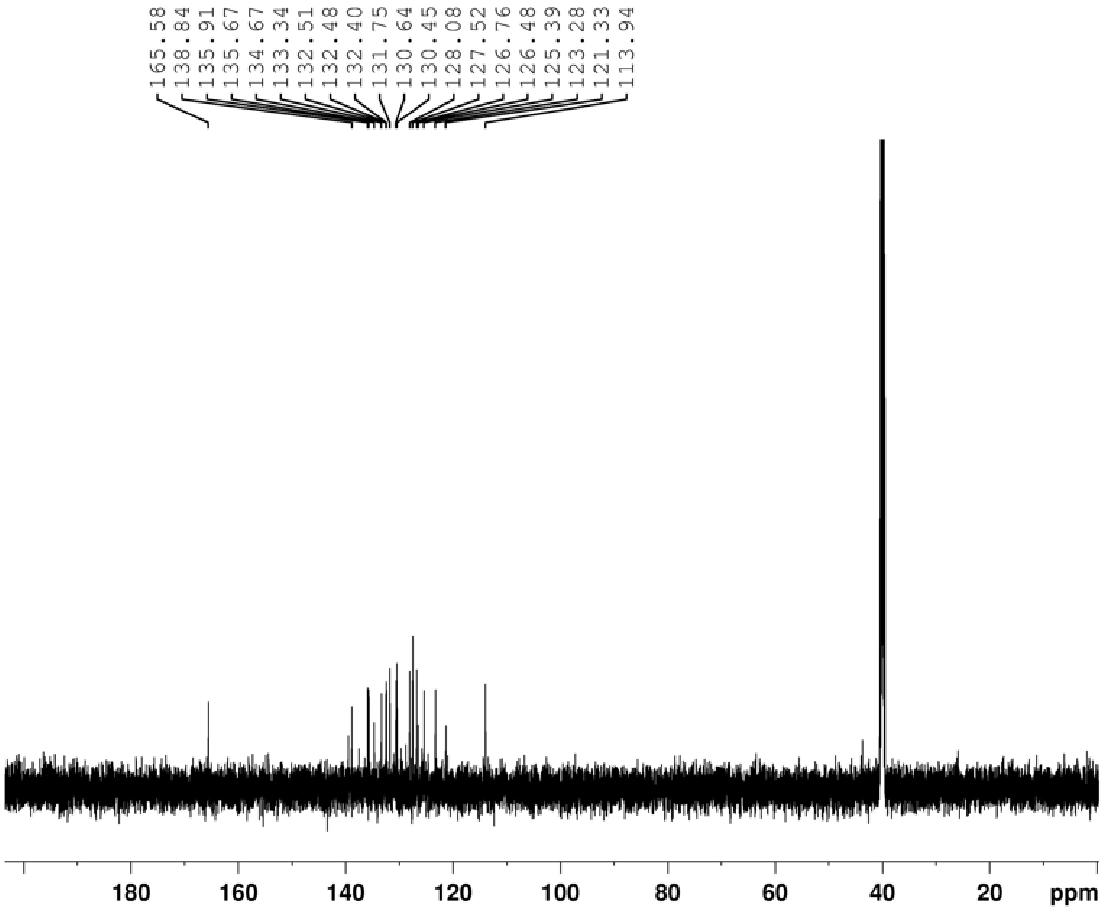

Figure S32. ^{13}C NMR spectrum of compound A6 (151 MHz, DMSO).

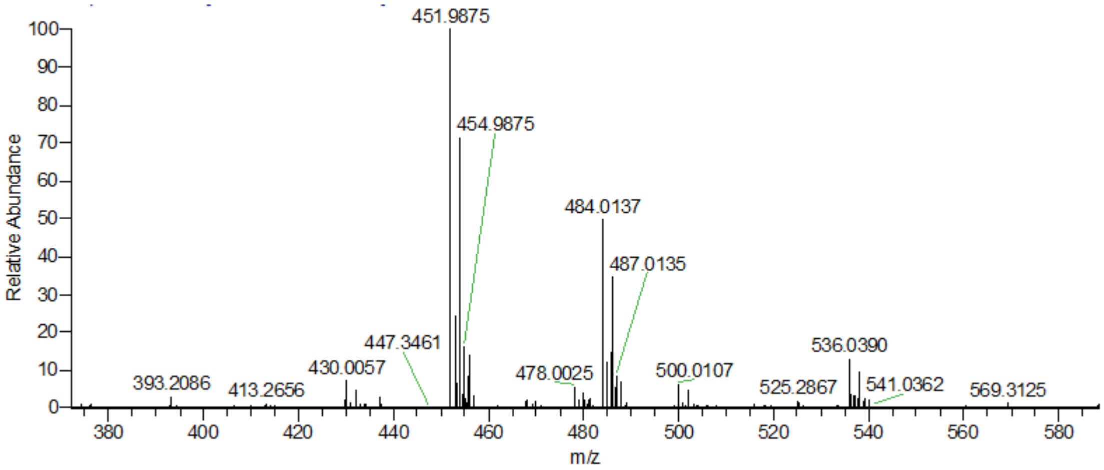

Figure S33. High resolution mass spectrum of compound A6.

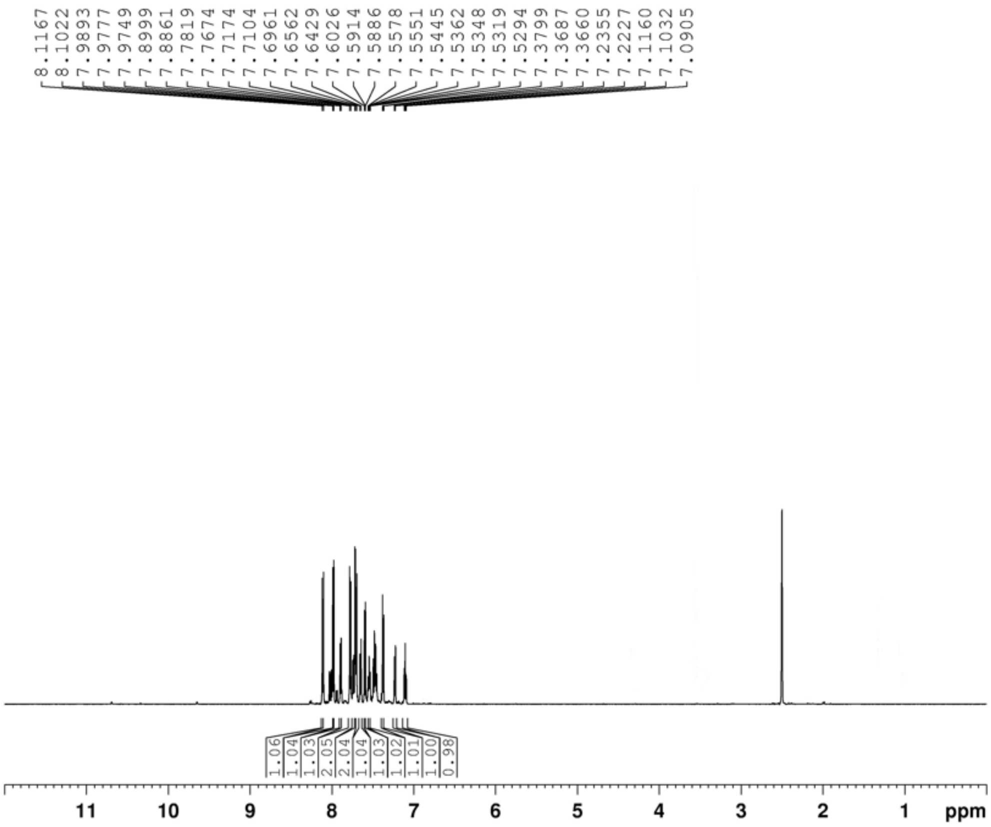

Figure S34. ^{1}H NMR spectrum of compound A7 (600 MHz, DMSO).

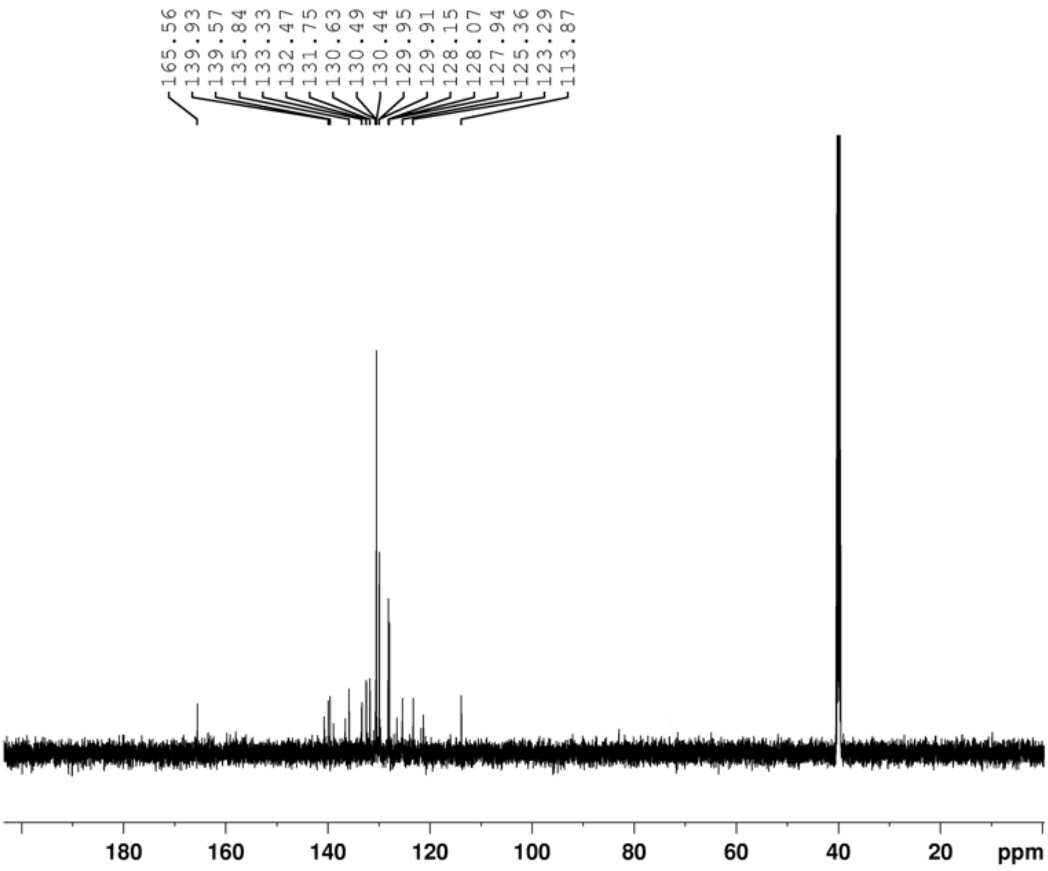

Figure S35. ^{13}C NMR spectrum of compound A7 (151 MHz, DMSO).

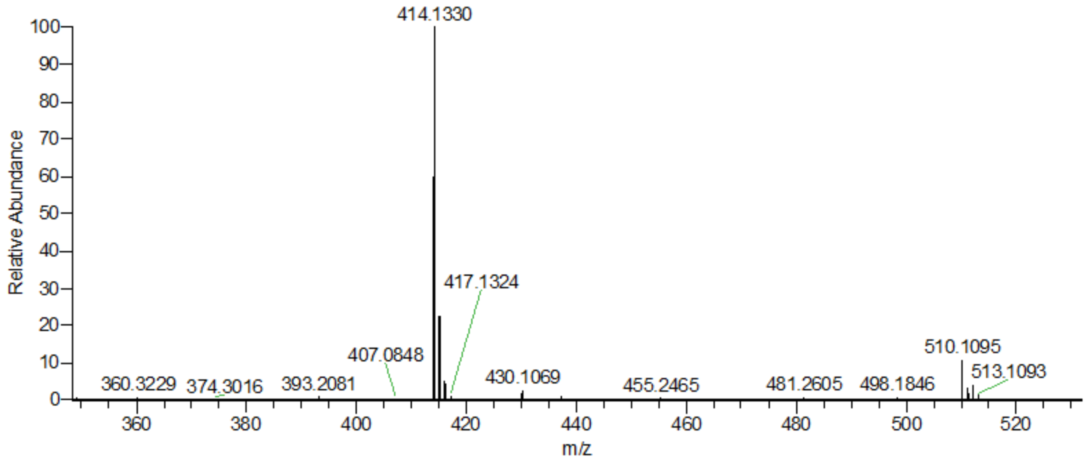

Figure S36. High resolution mass spectrum of compound A7.

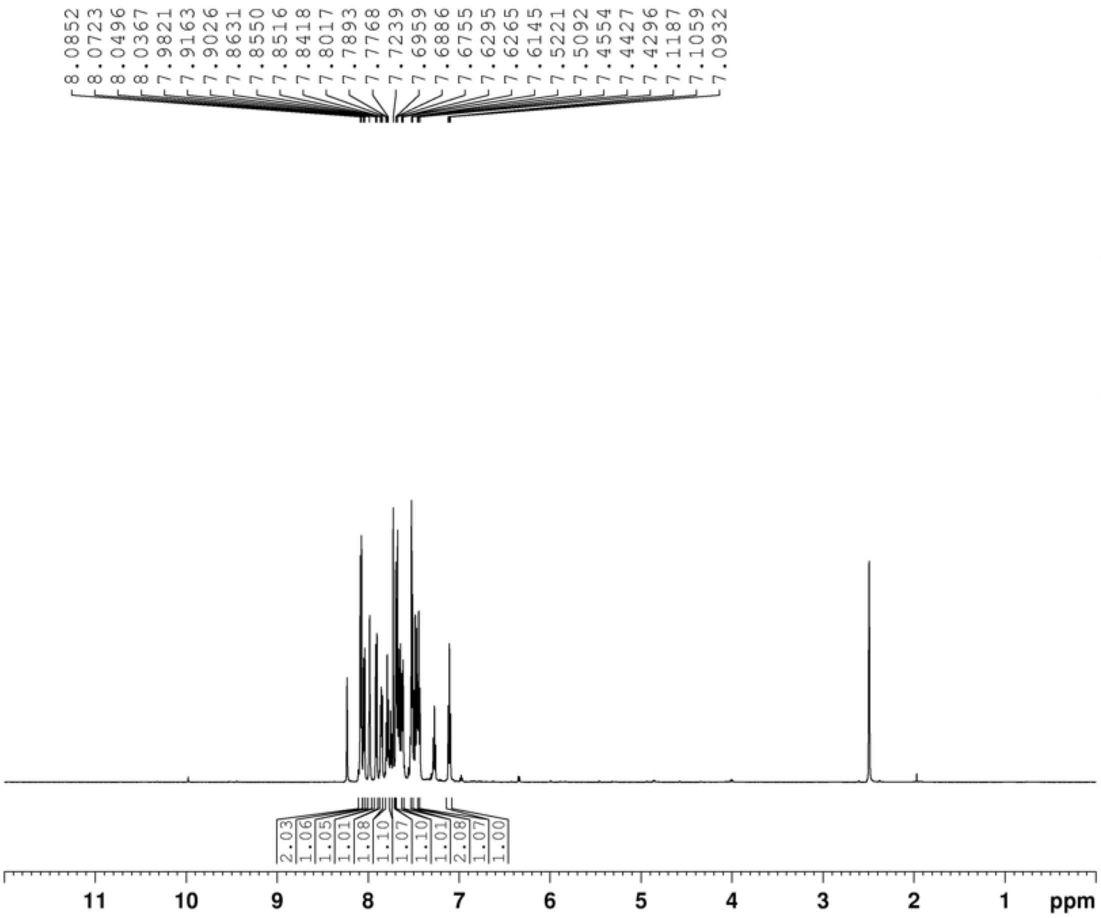

Figure S37. ^{1}H NMR spectrum of compound A8 (600 MHz, DMSO).

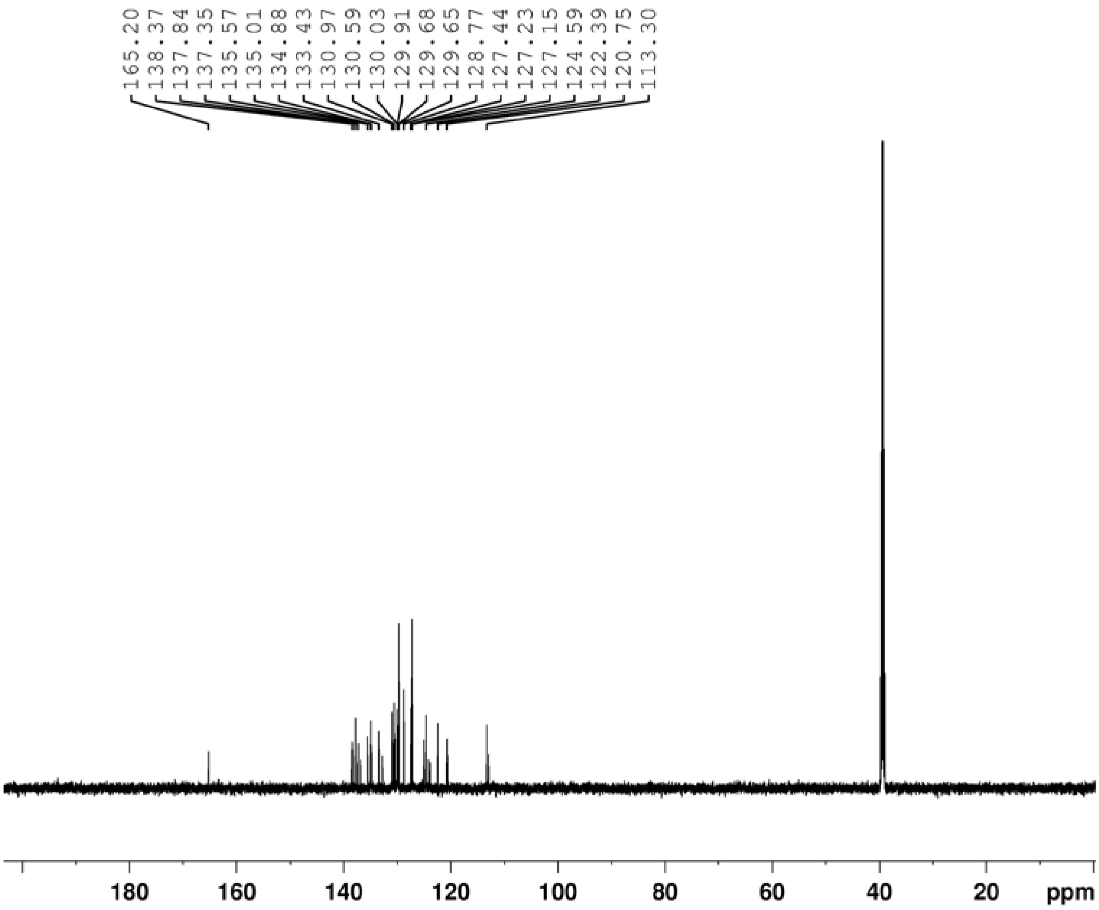

Figure S38. ^{13}C NMR spectrum of compound A8 (151 MHz, DMSO).

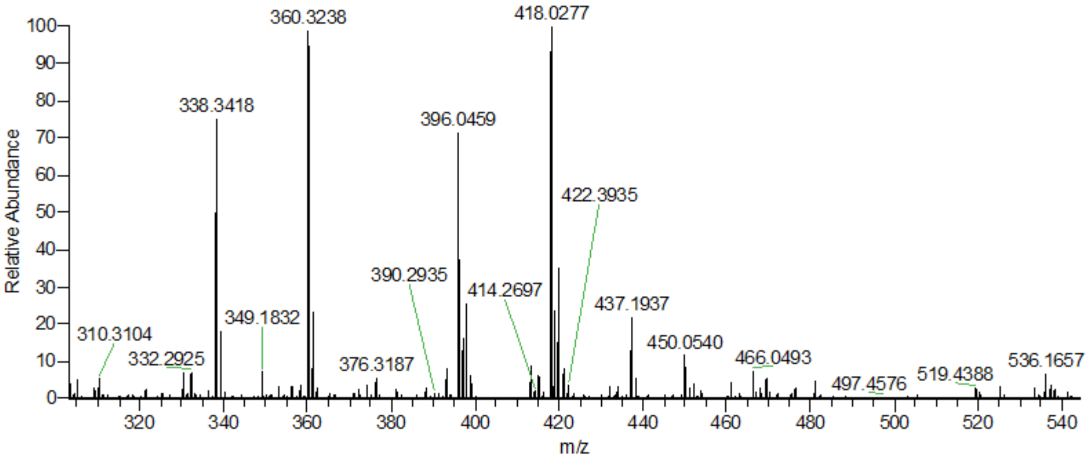

Figure S39. High resolution mass spectrum of compound A8.

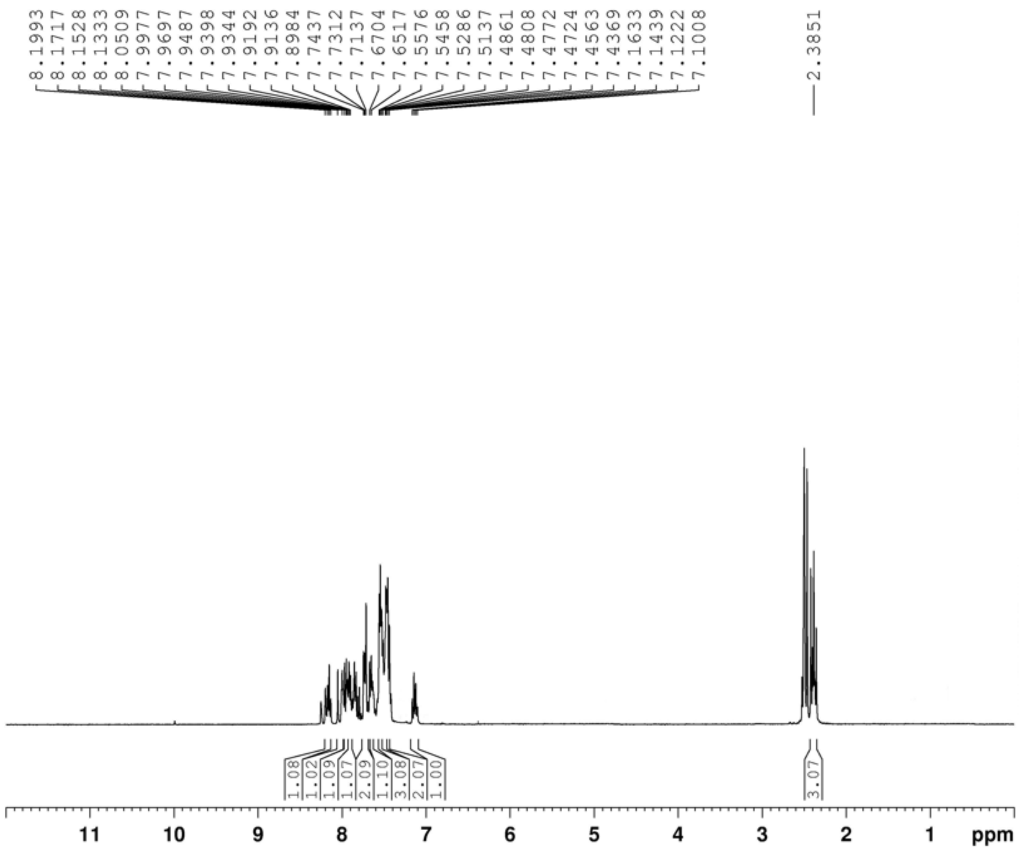

Figure S40. ^{1}H NMR spectrum of compound A9 (400 MHz, DMSO).

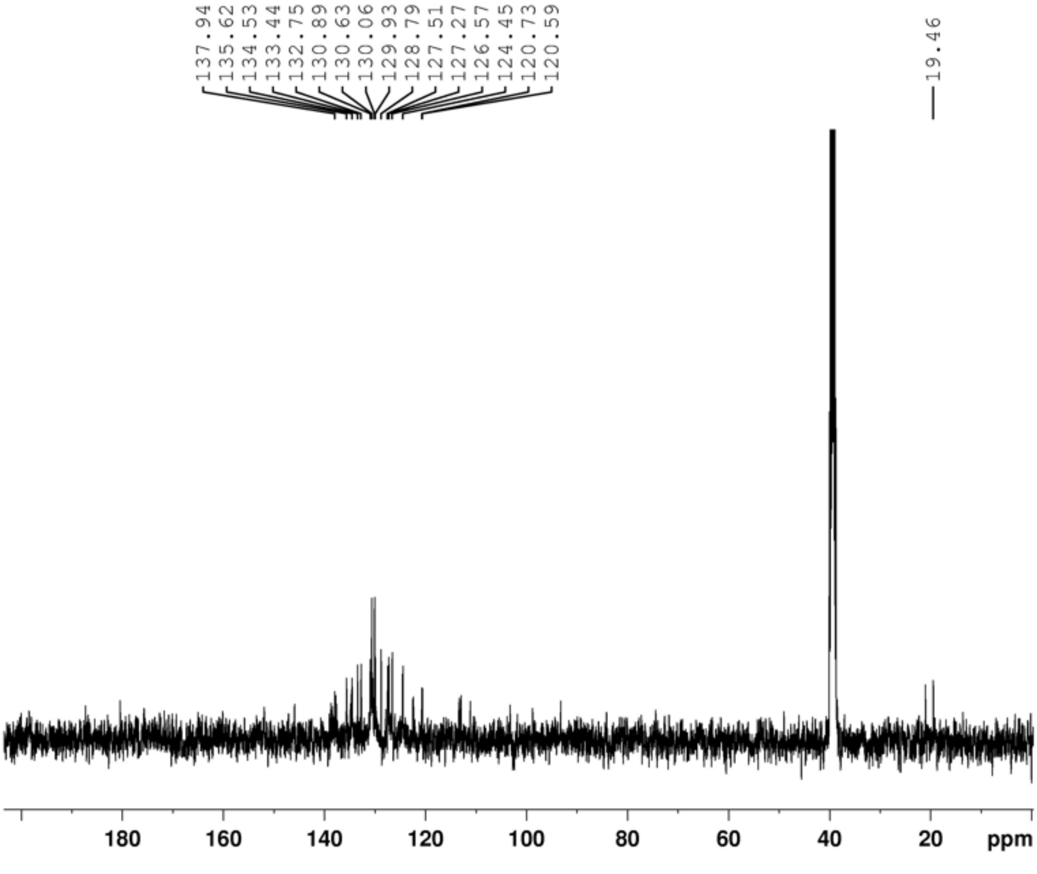

Figure S41. ^{13}C NMR spectrum of compound A9 (101 MHz, DMSO).

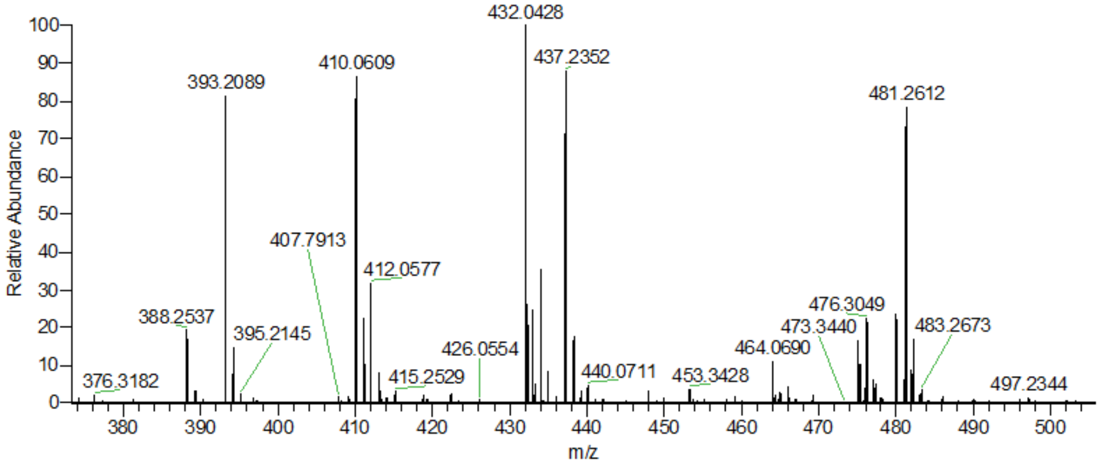

Figure S42. High resolution mass spectrum of compound A9.

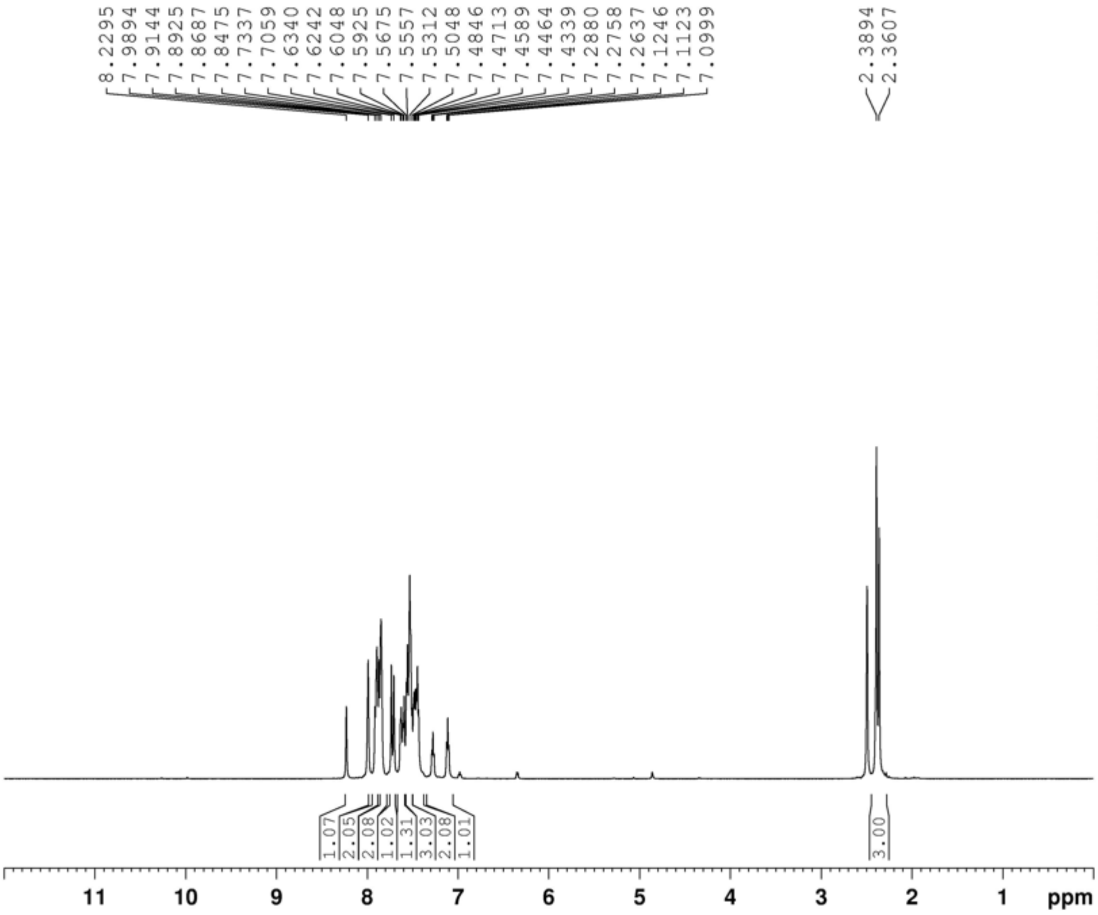

Figure S43. ^{1}H NMR spectrum of compound A10 (600 MHz, DMSO).

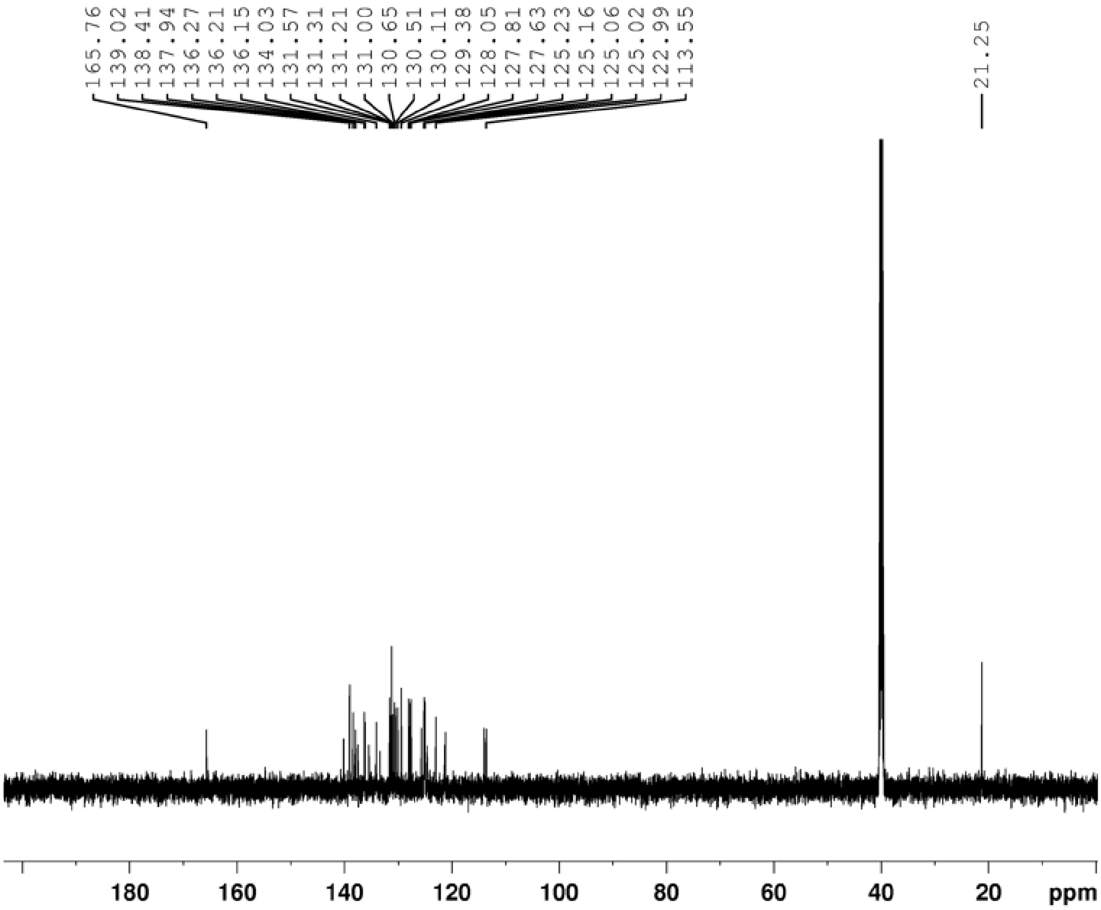

Figure S44. ^{13}C NMR spectrum of compound A10 (151 MHz, DMSO).

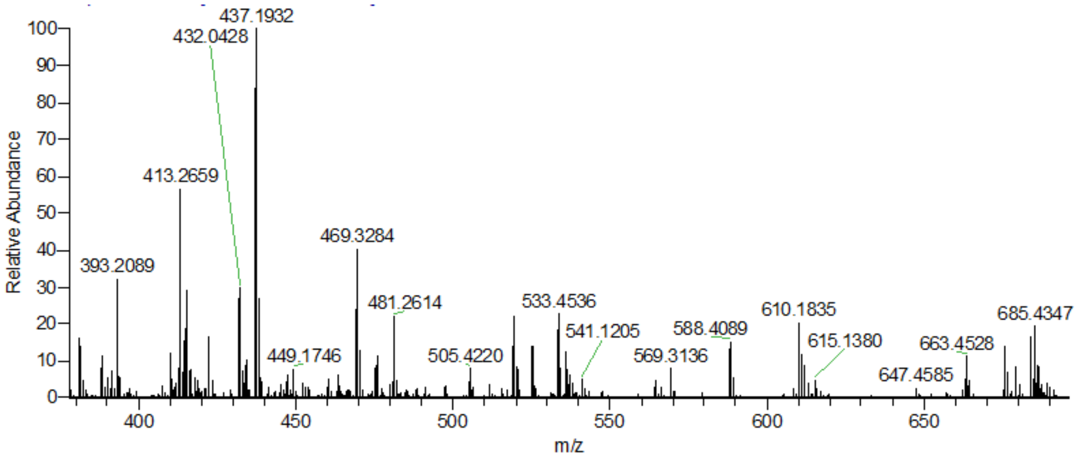

Figure S45. High resolution mass spectrum of compound A10.

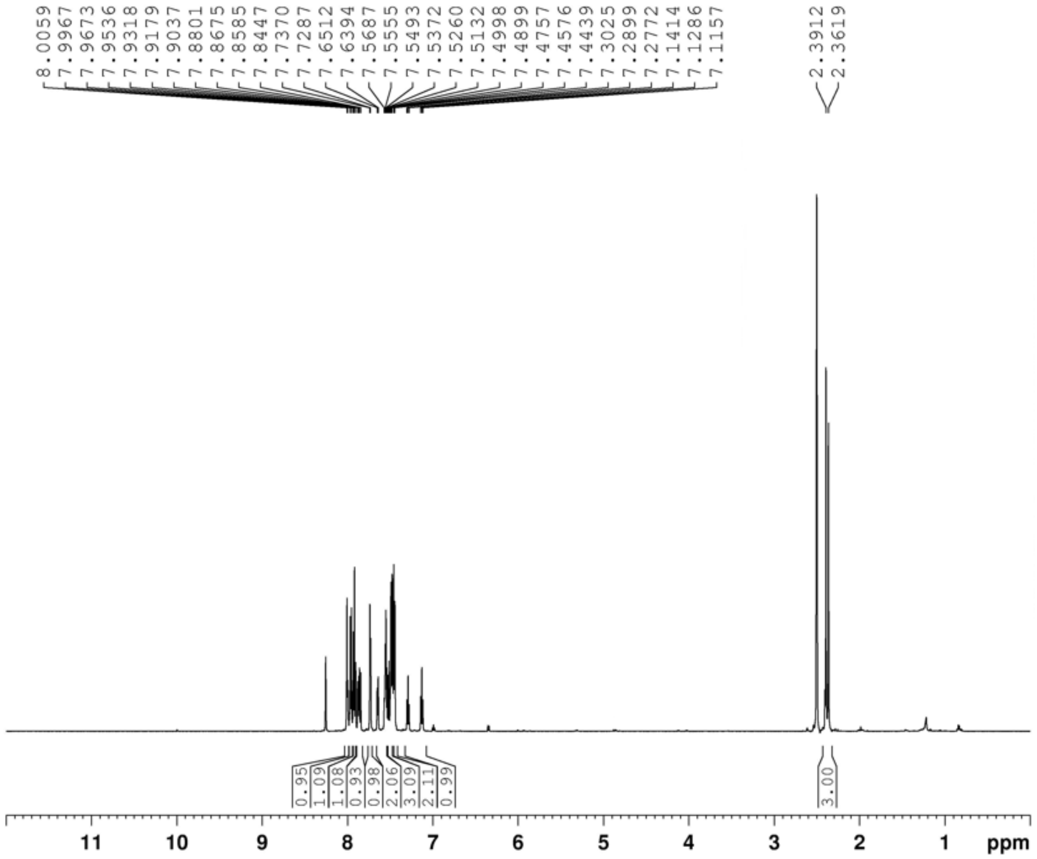

Figure S46. ^1H NMR spectrum of compound A11 (600 MHz, DMSO).

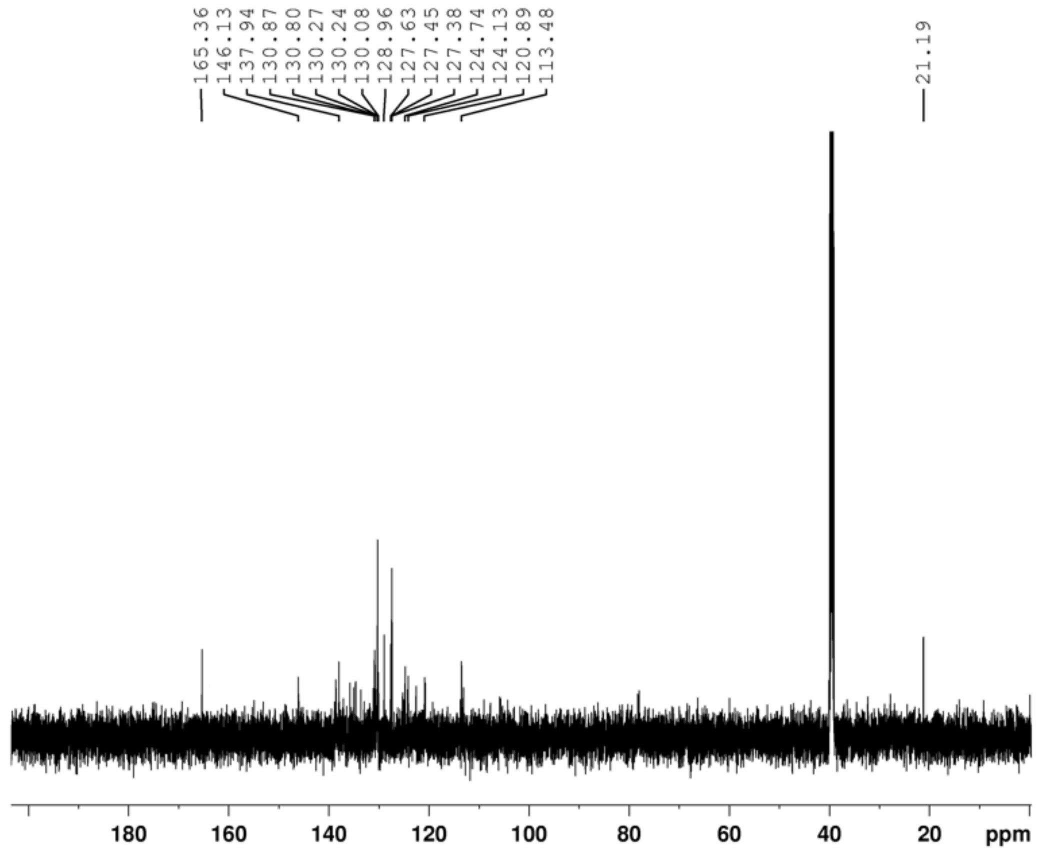

Figure S47. ^{13}C NMR spectrum of compound A11 (151 MHz, DMSO).

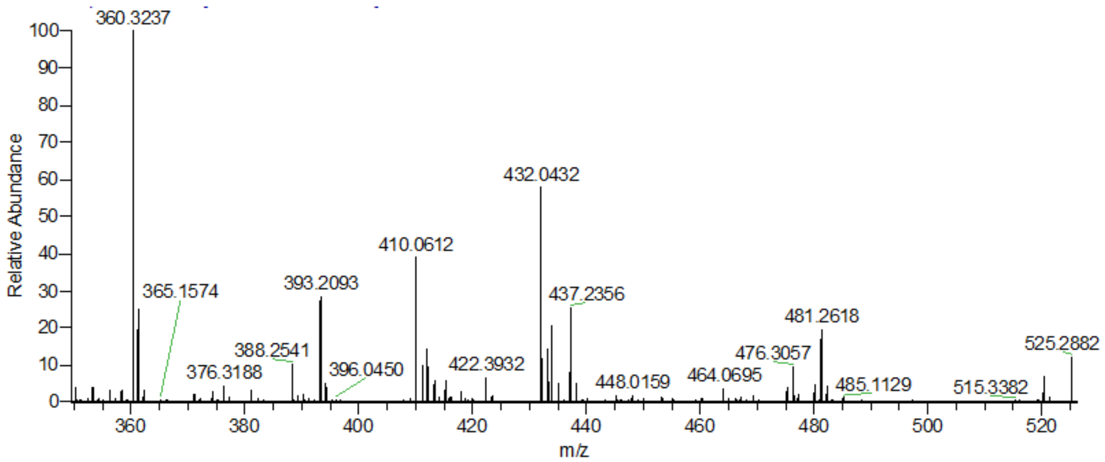

Figure S48. High resolution mass spectrum of compound A11.

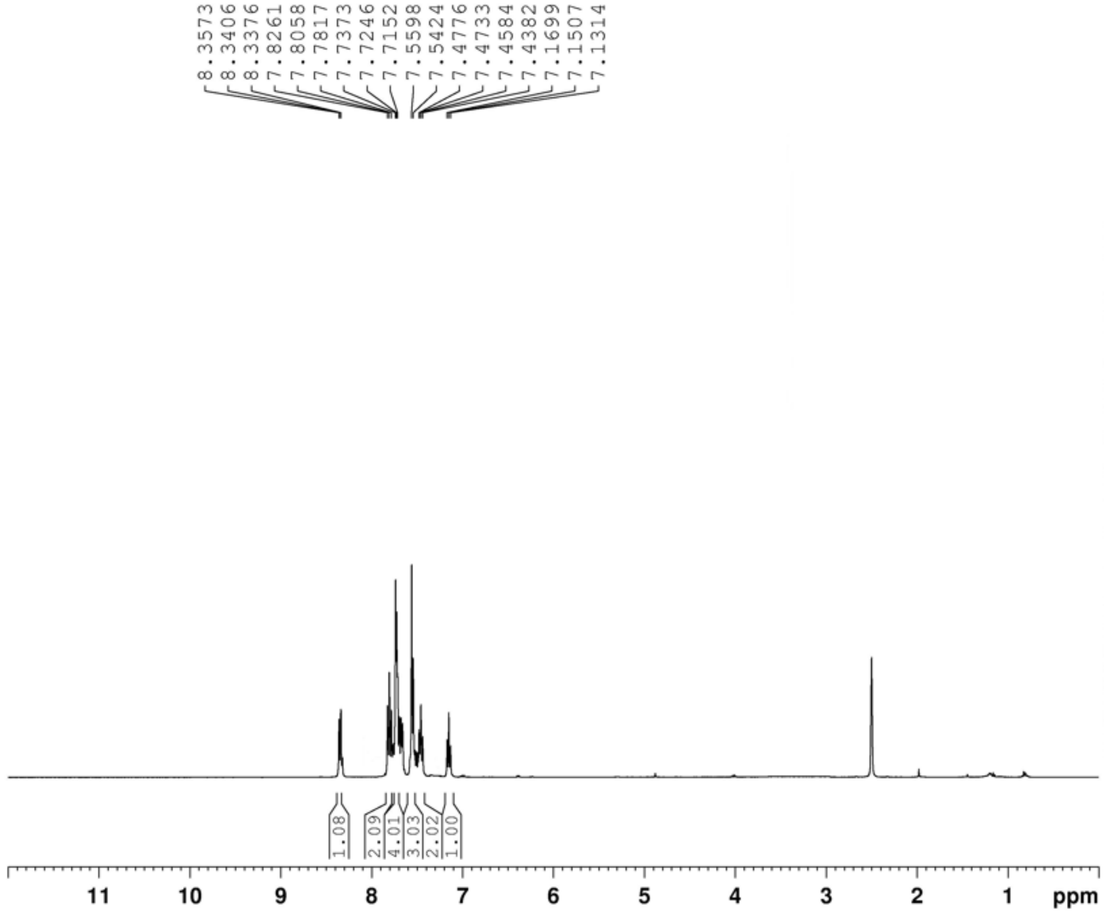

Figure S49. ^{1}H NMR spectrum of compound A12 (400 MHz, DMSO).

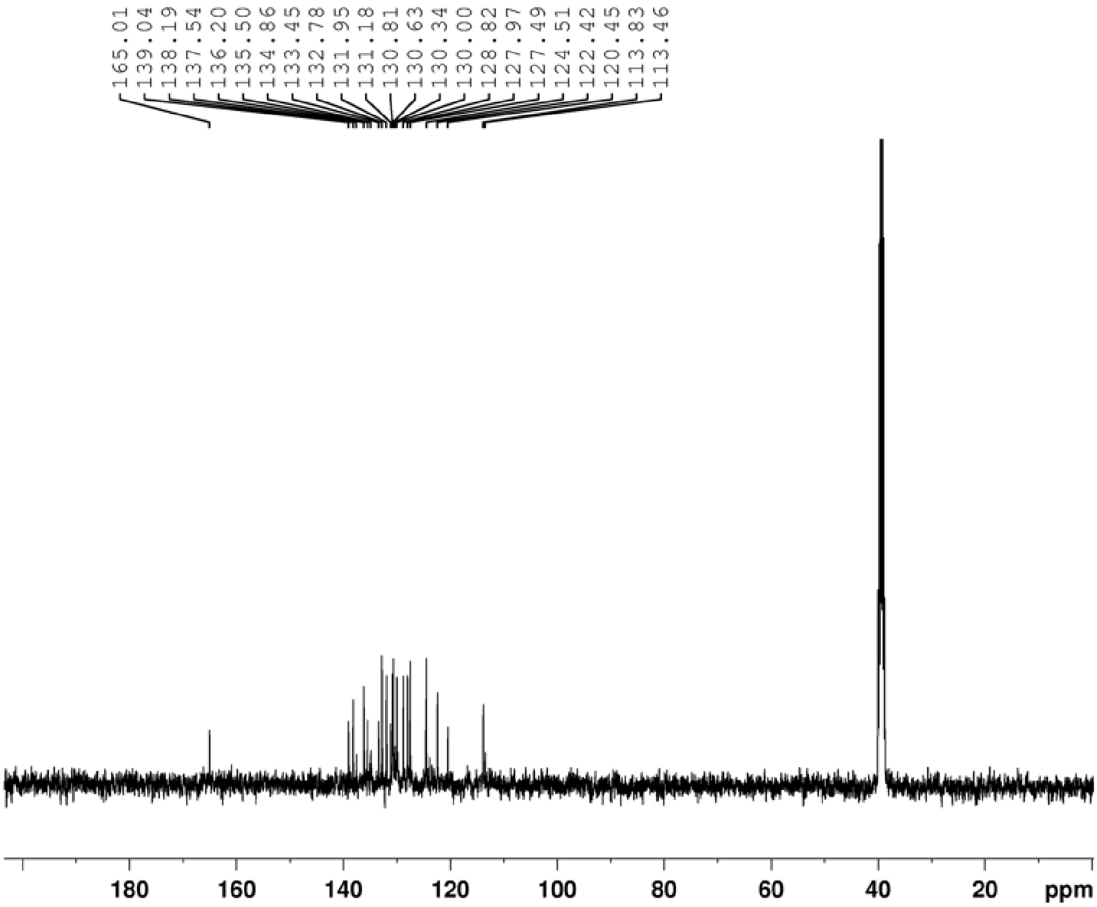

Figure S50. ^{13}C NMR spectrum of compound A12 (101 MHz, DMSO).

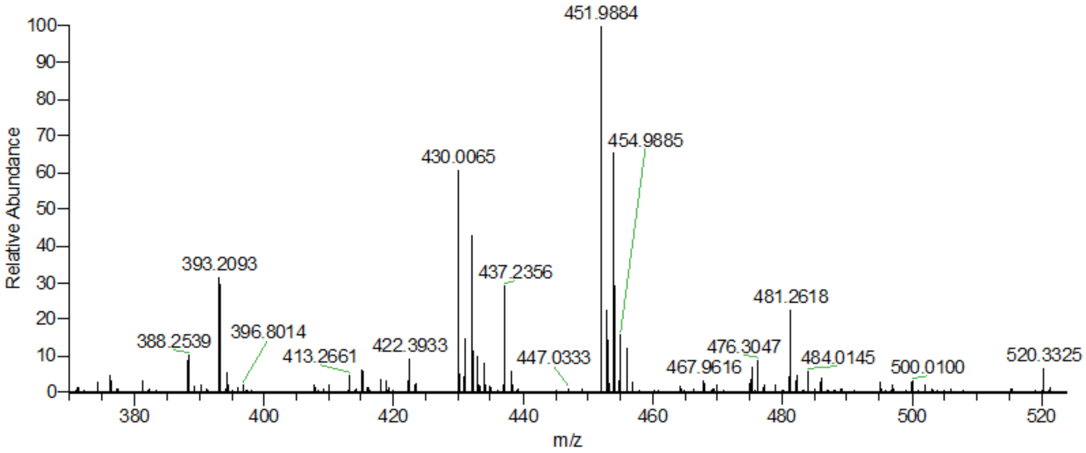

Figure S51. High resolution mass spectrum of compound A12.

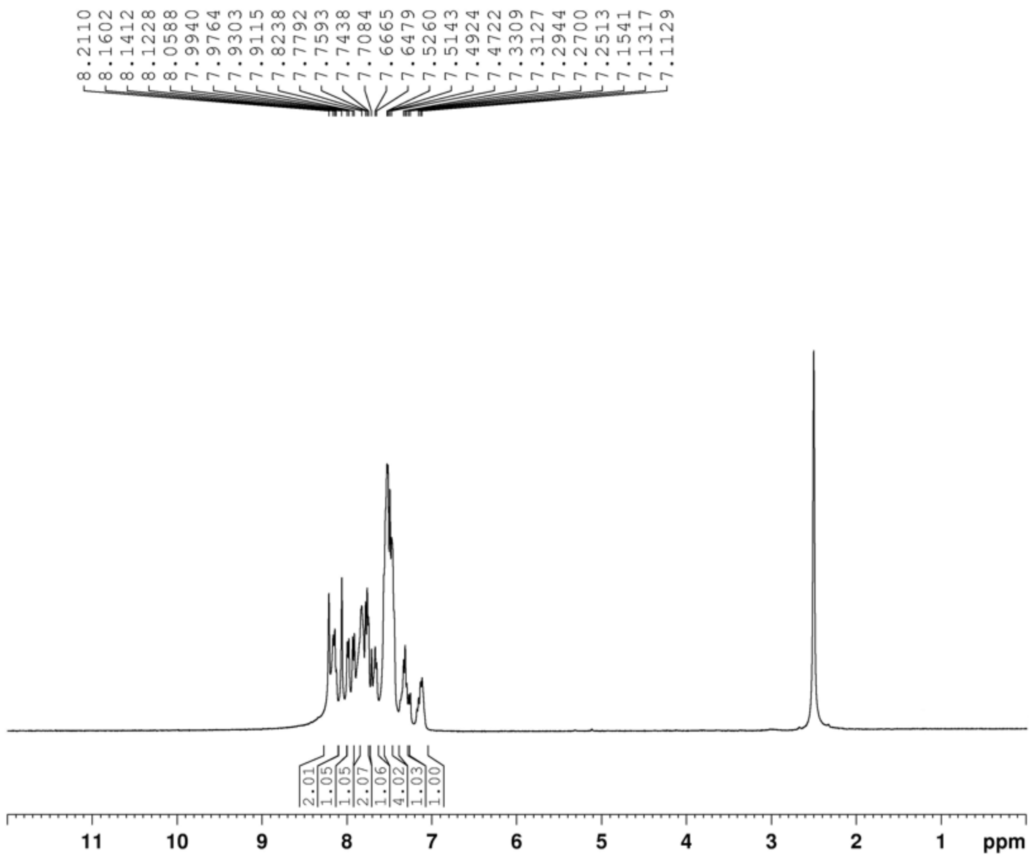

Figure S52. H NMR spectrum of compound A13 (400 MHz, DMSO).

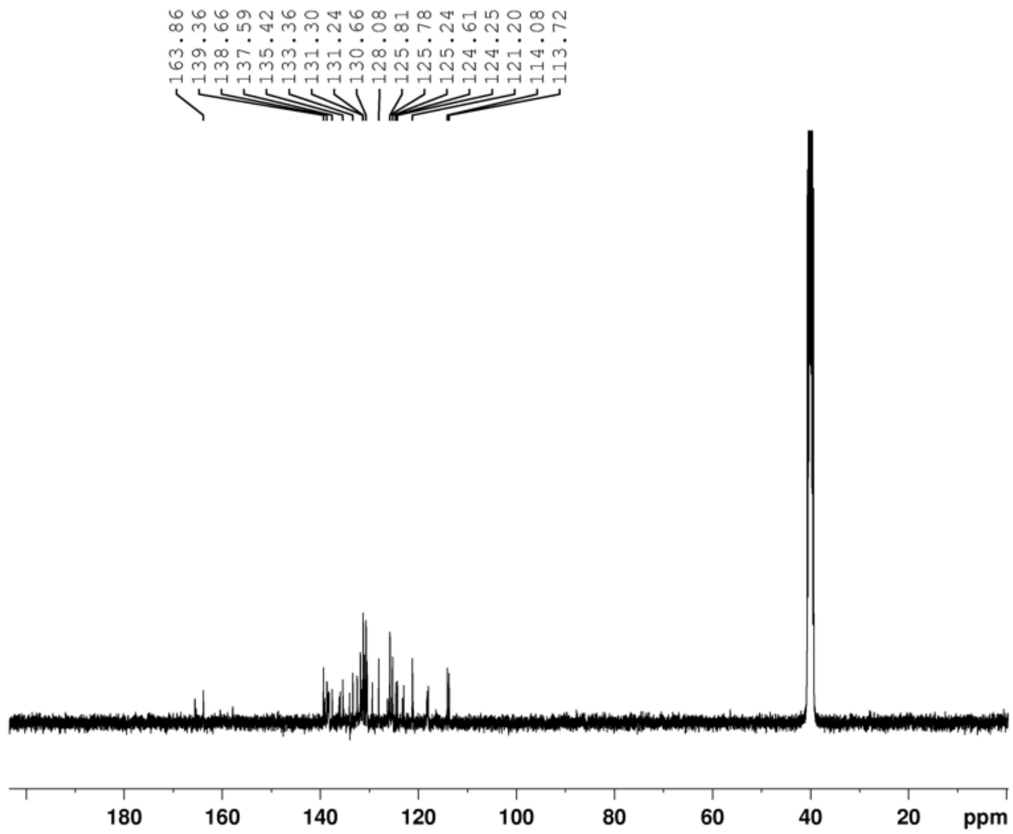

Figure S53. C NMR spectrum of compound A13 (101 MHz, DMSO).

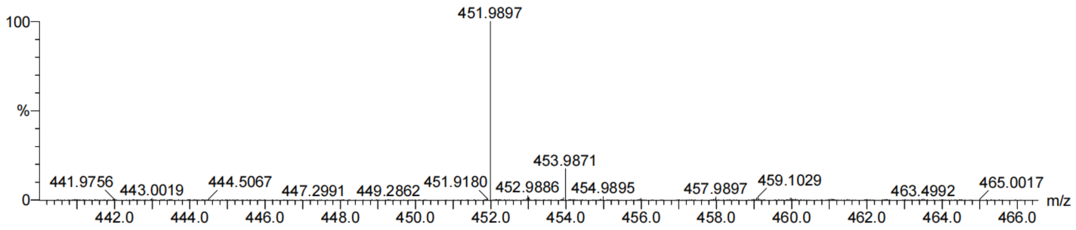

Figure S54. High resolution mass spectrum of compound A13.

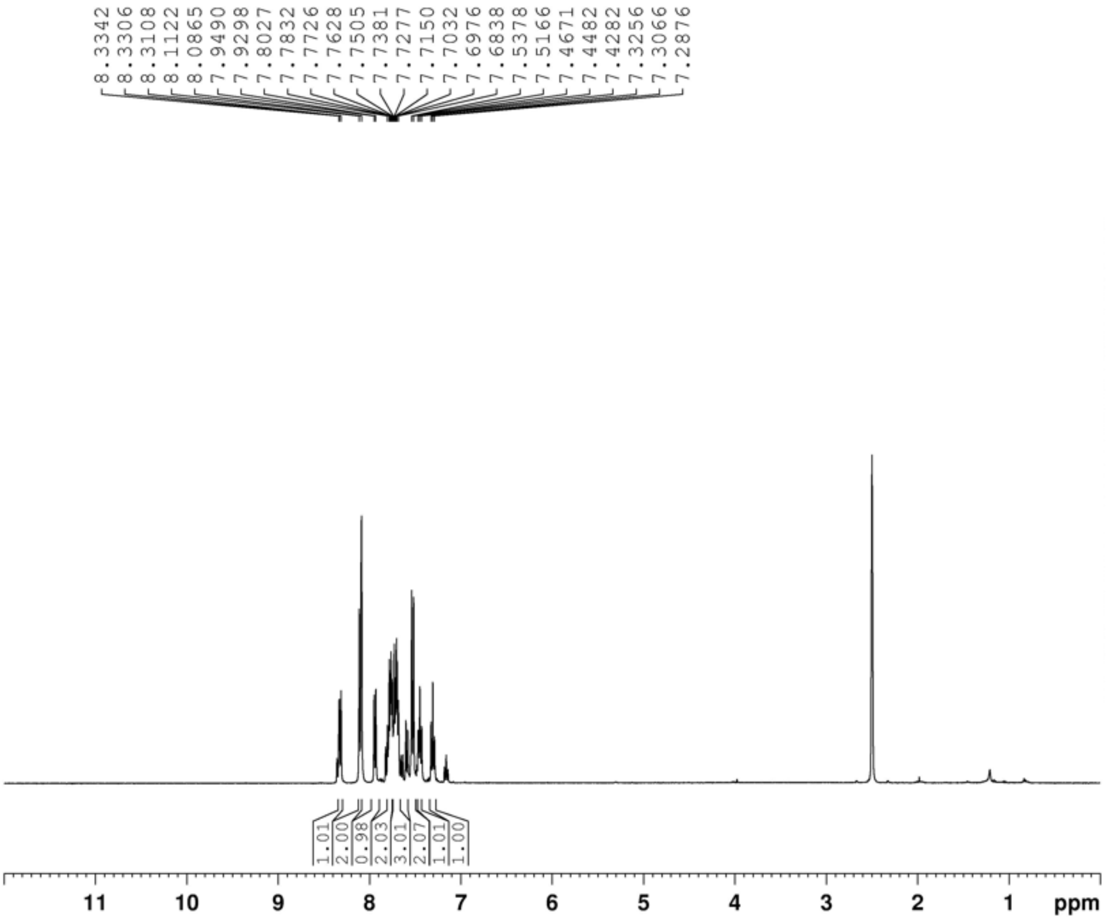

Figure S55. ^{1}H NMR spectrum of compound A14 (400 MHz, DMSO).

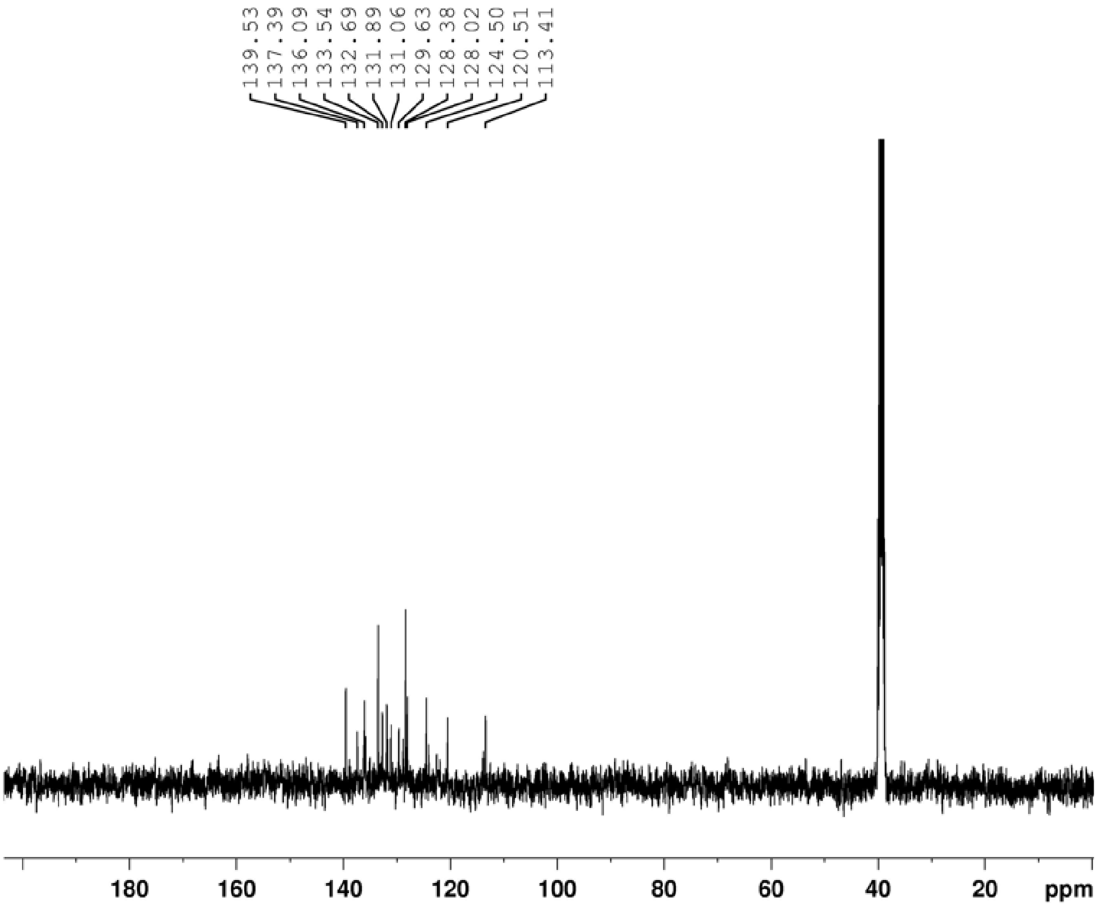

Figure S56. ^{13}C NMR spectrum of compound A14 (101 MHz, DMSO).

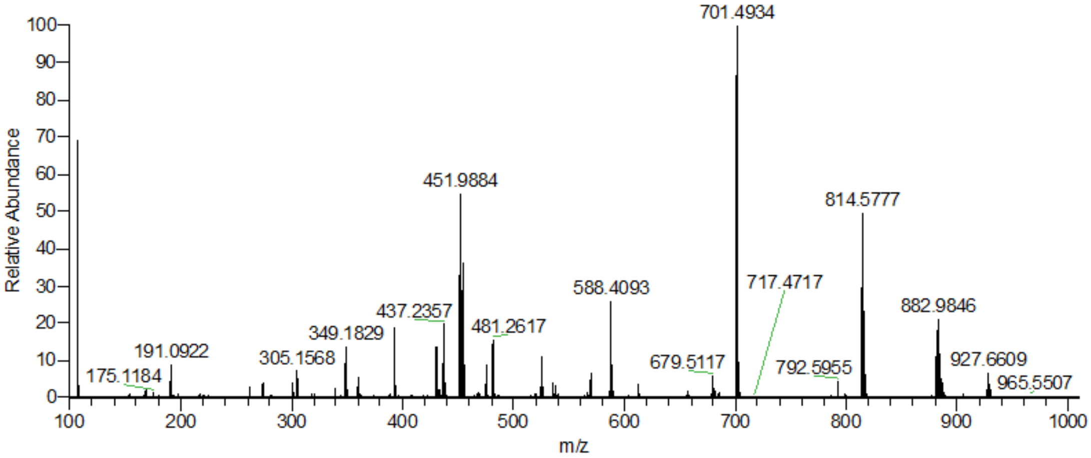

Figure S57. High resolution mass spectrum of compound A14.

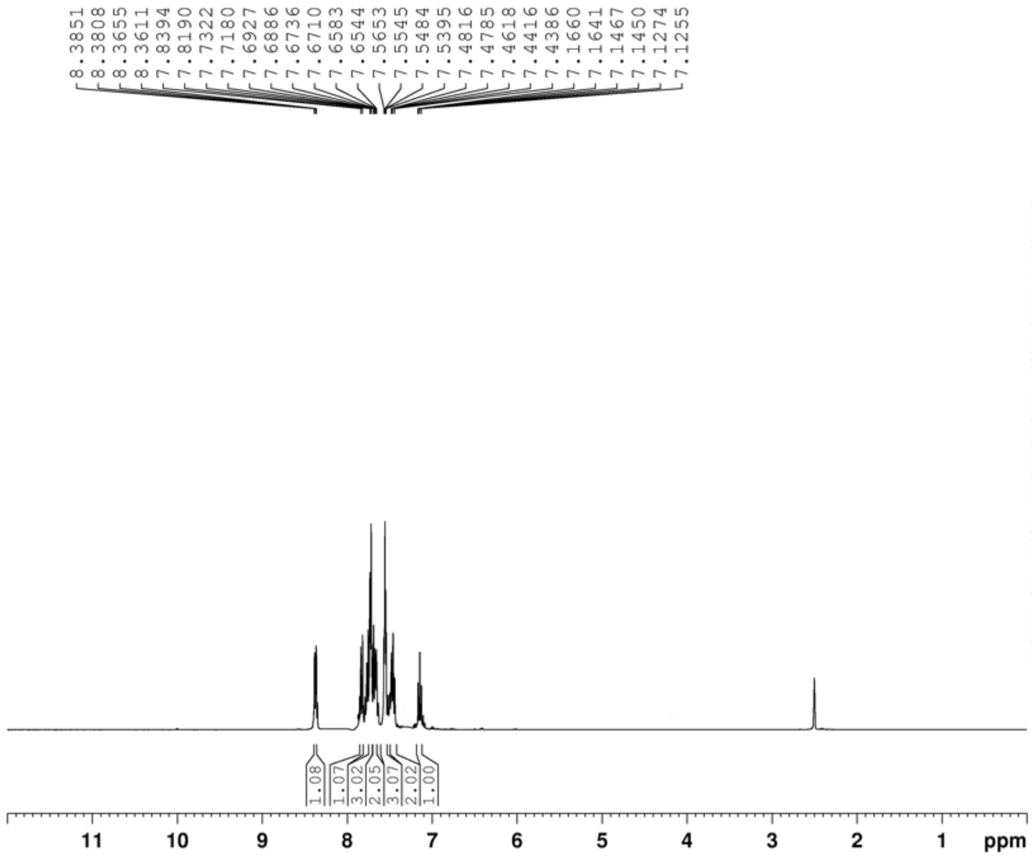

Figure S58. ^{1}H NMR spectrum of compound A15 (400 MHz, DMSO).

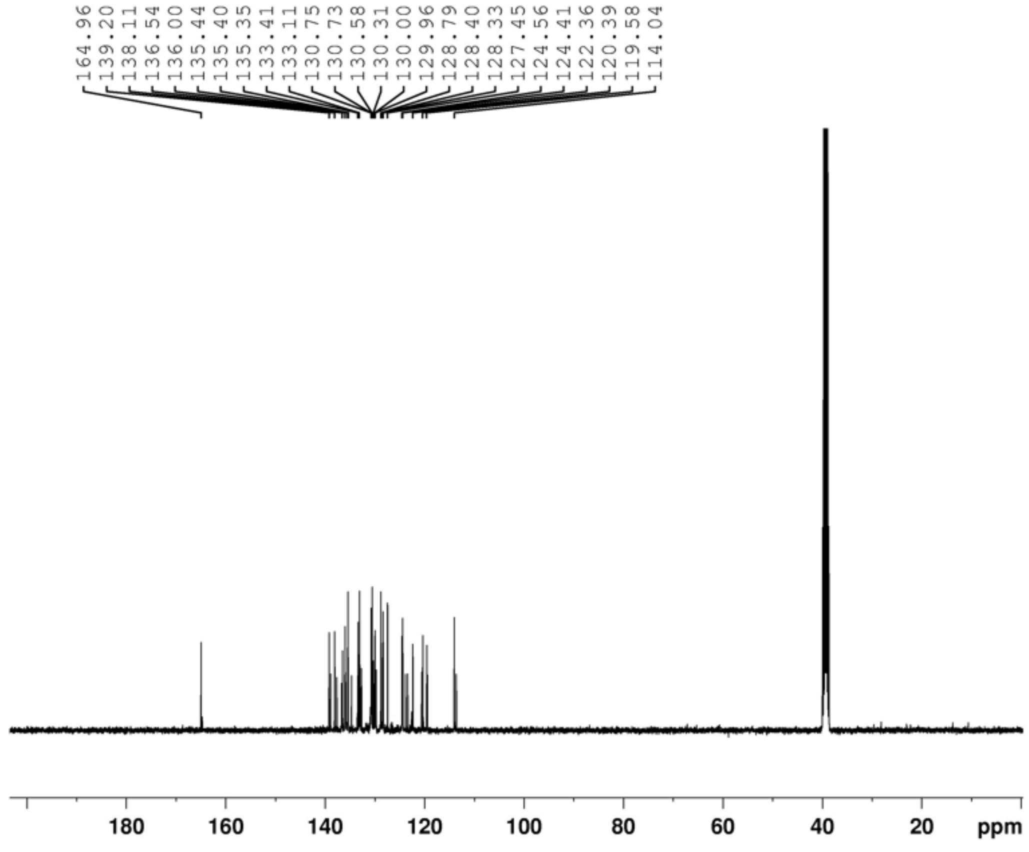

Figure S59. ^{13}C NMR spectrum of compound A15 (101 MHz, DMSO).

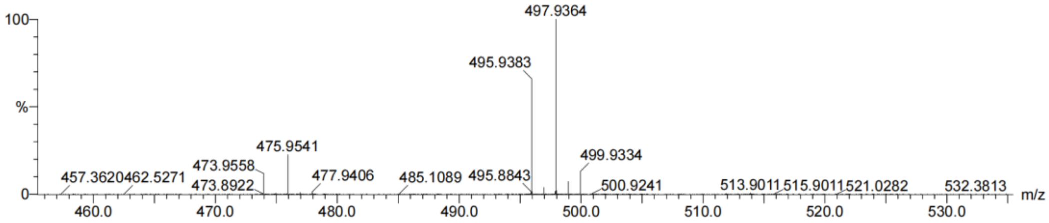

Figure S60. High resolution mass spectrum of compound A15.

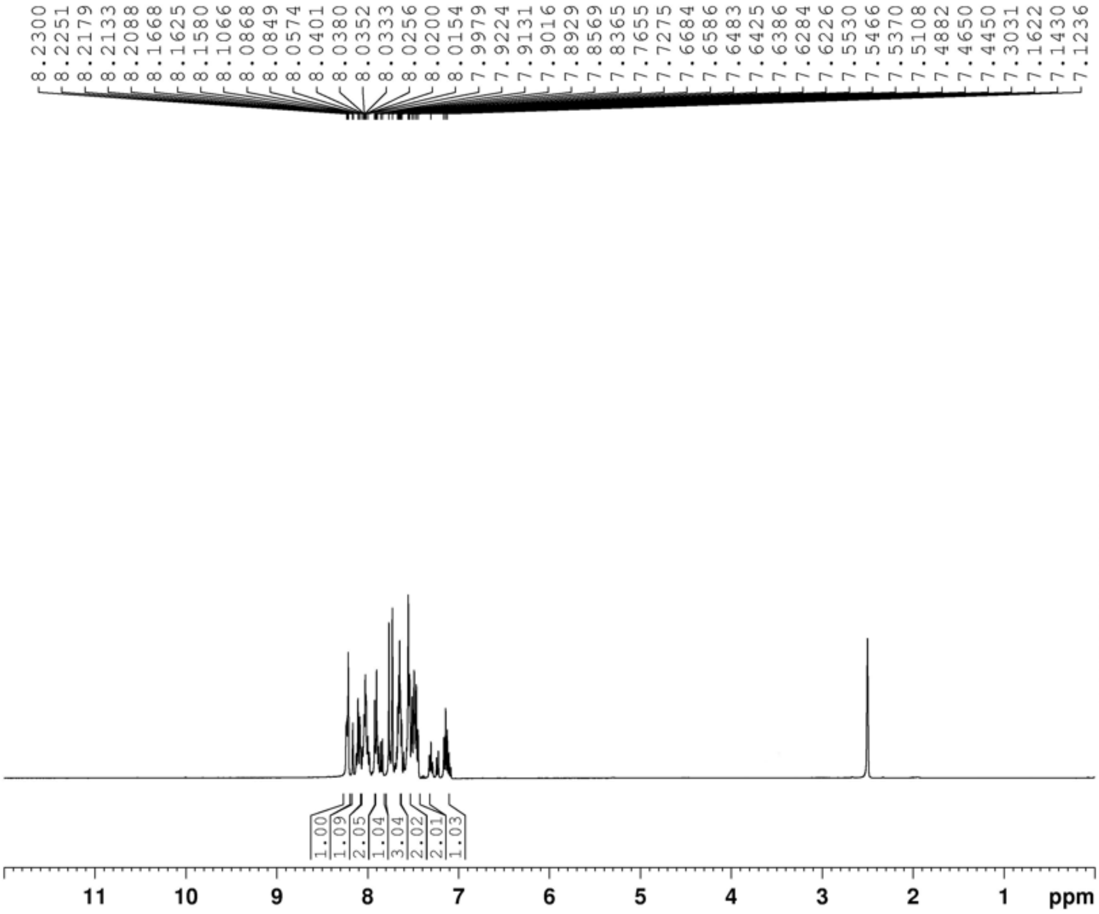

Figure S61. ^{1}H NMR spectrum of compound A16 (400 MHz, DMSO).

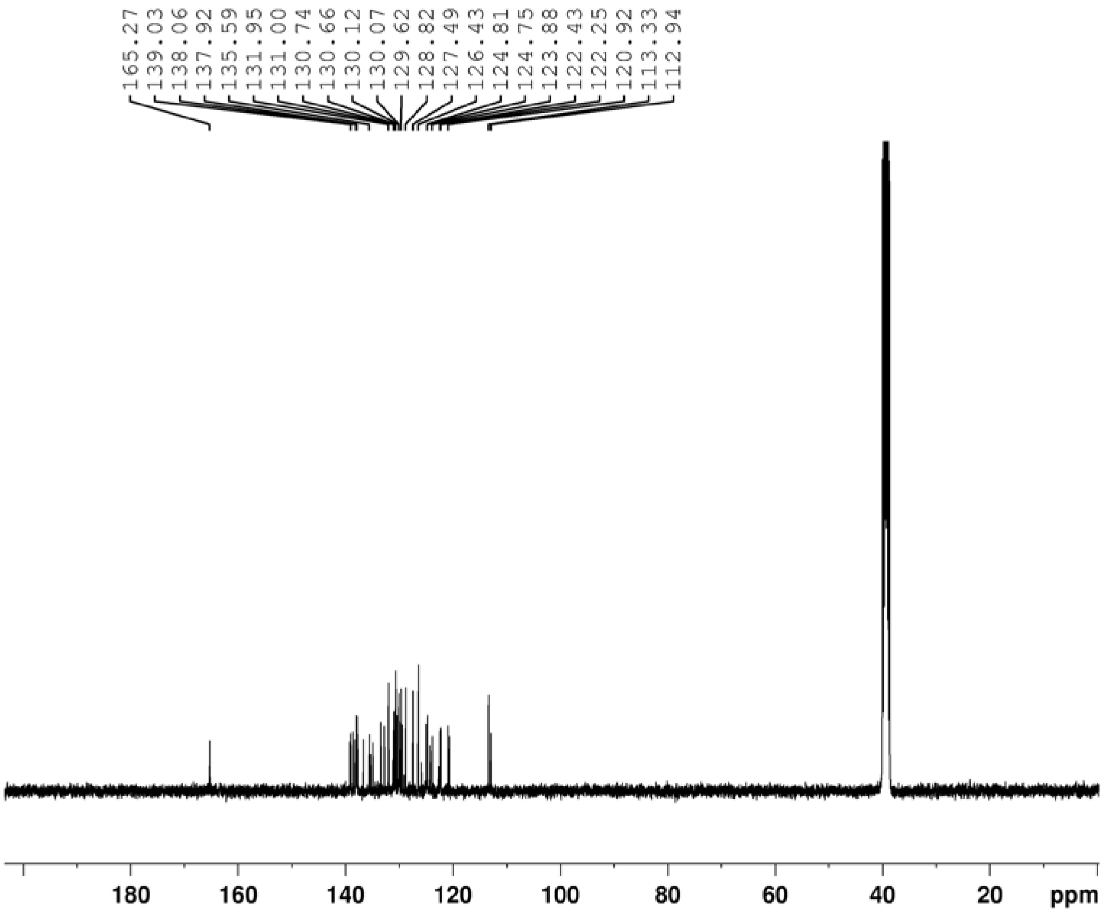

Figure S62. ^{13}C NMR spectrum of compound A16 (101 MHz, DMSO).

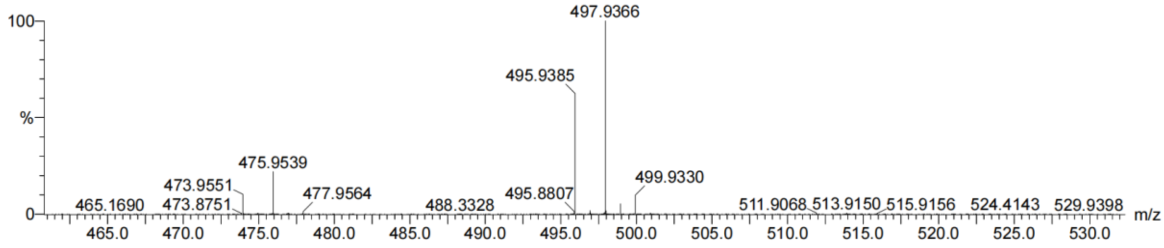

Figure S63. High resolution mass spectrum of compound A16.

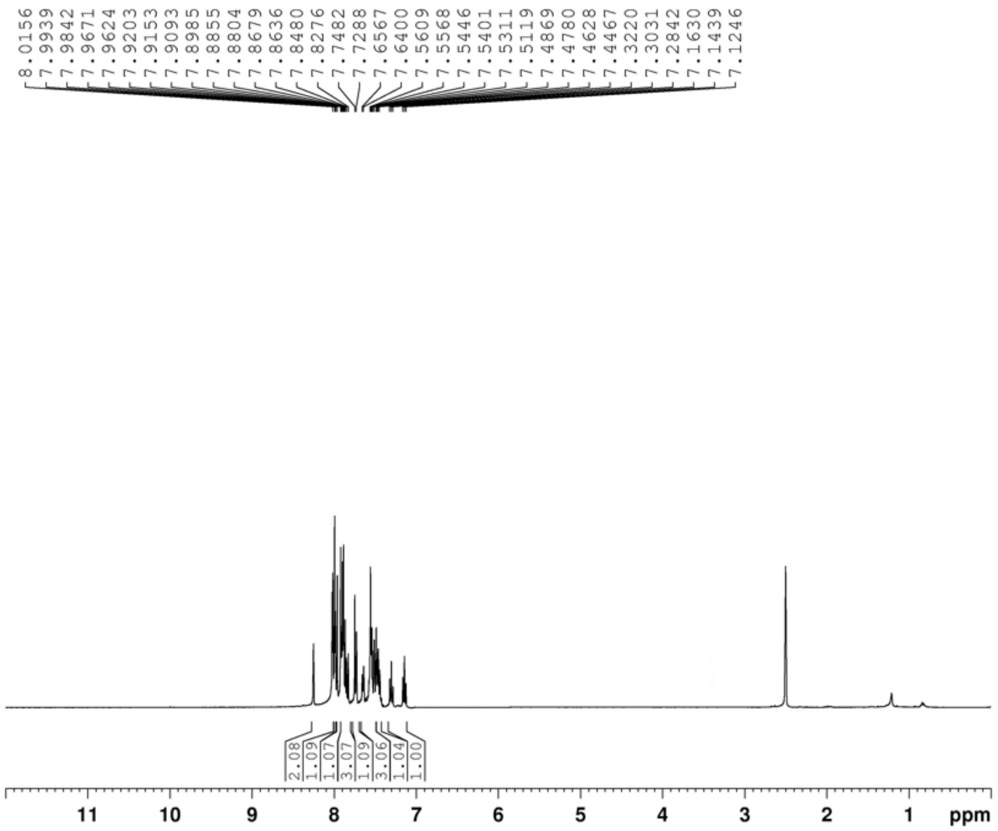

Figure S64. ^{1}H NMR spectrum of compound A17 (400 MHz, DMSO).

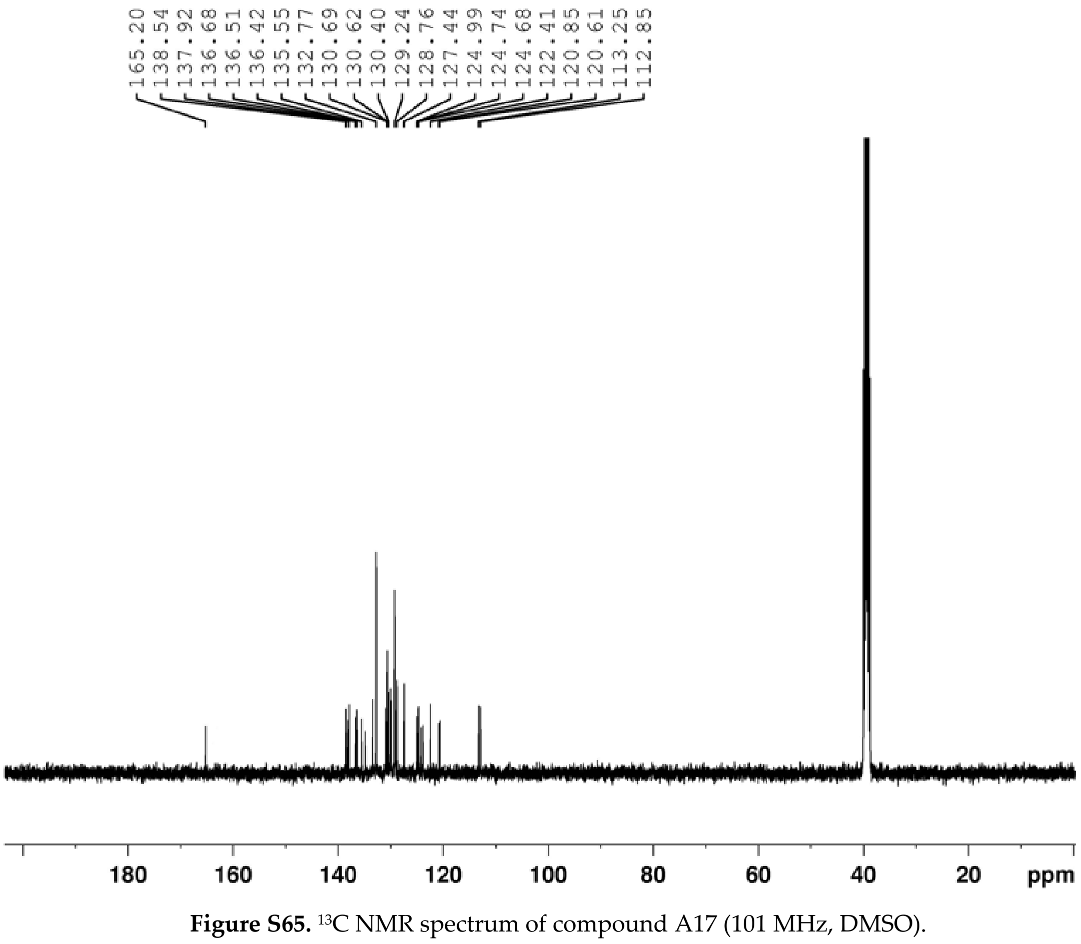

Figure S65. ^{13}C NMR spectrum of compound A17 (101 MHz, DMSO).

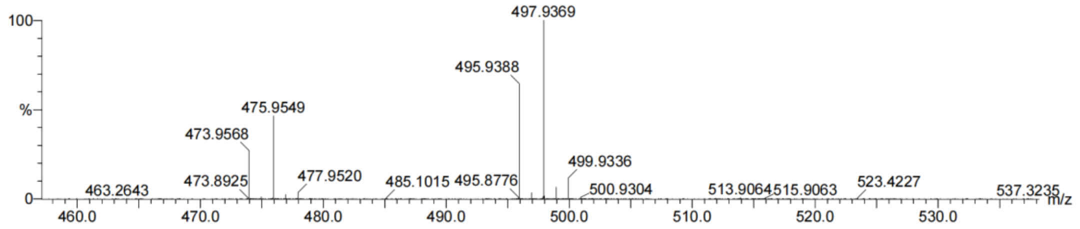

Figure S66. High resolution mass spectrum of compound A17.

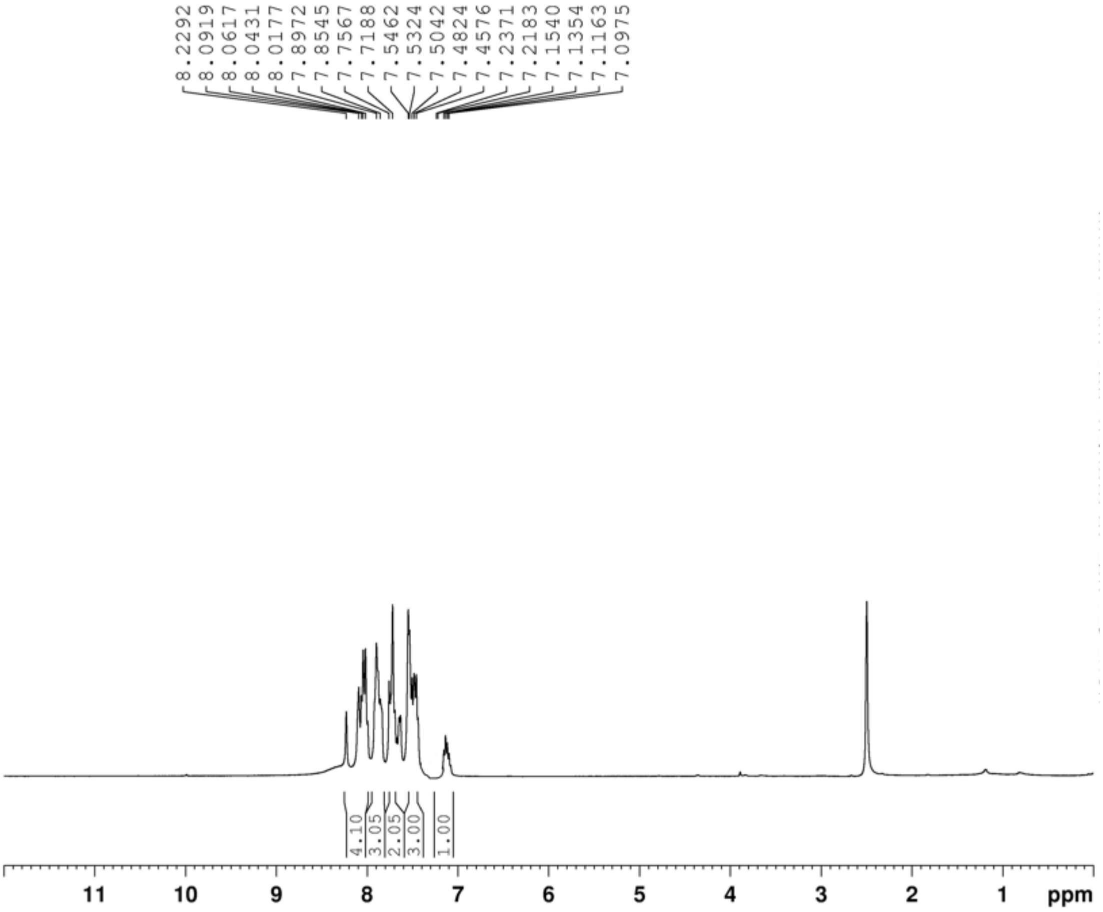

Figure S67. ^{1}H NMR spectrum of compound A18 (400 MHz, DMSO).

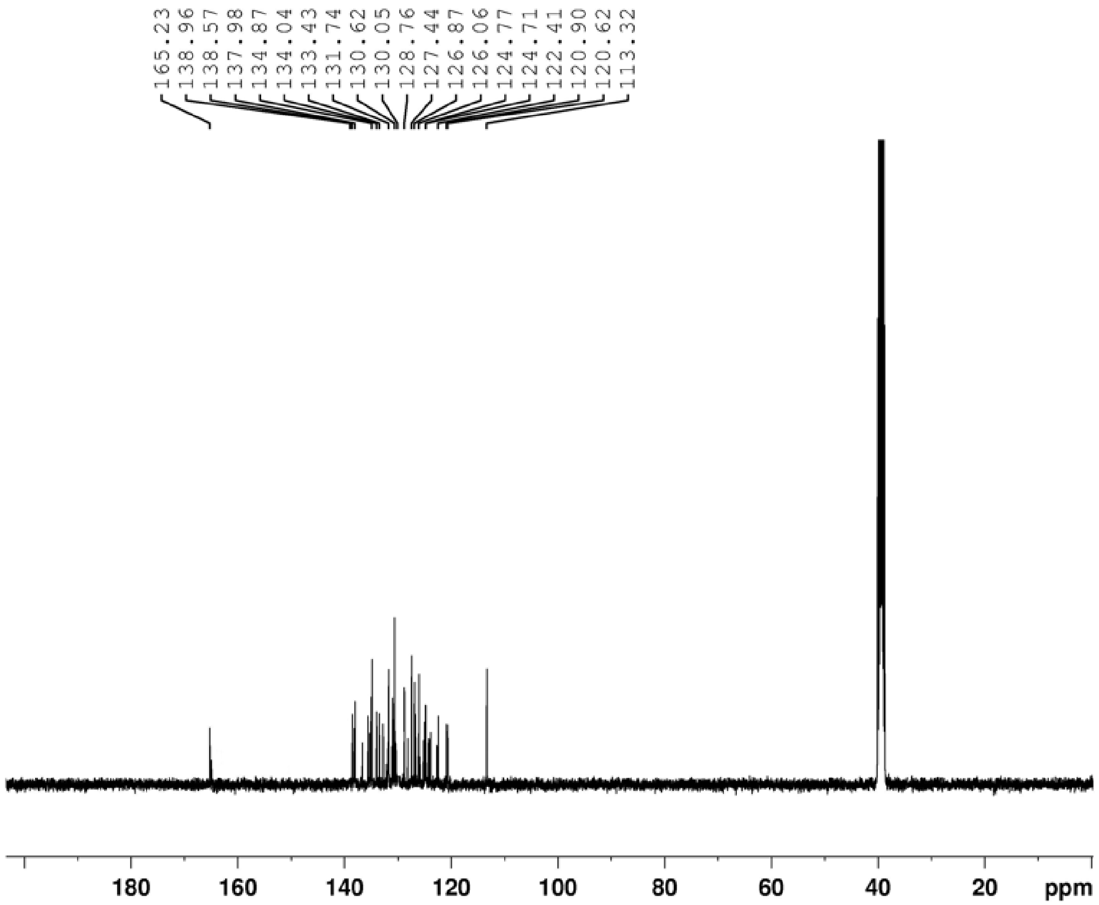

Figure S68. ^{13}C NMR spectrum of compound A18 (101 MHz, DMSO).

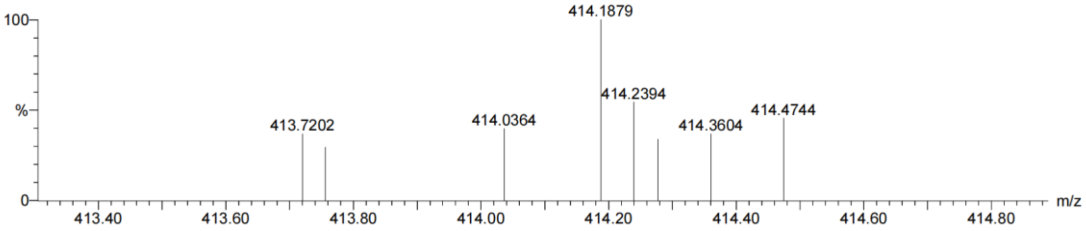

Figure S69. High resolution mass spectrum of compound A18.

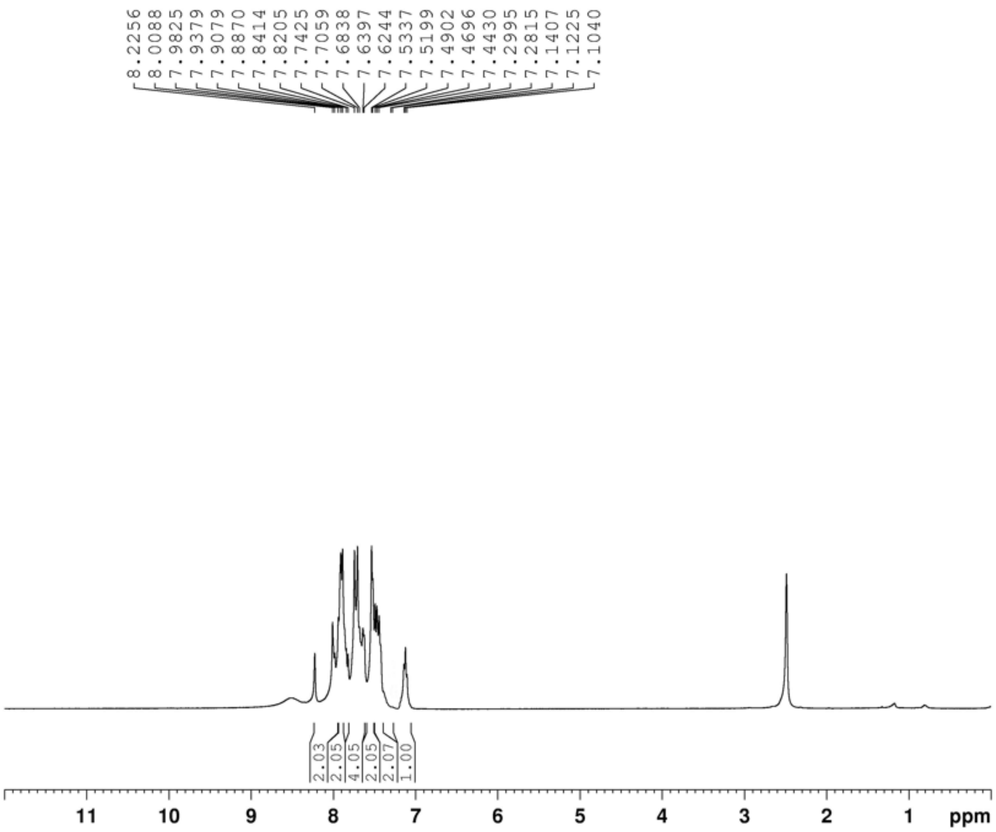

Figure S70. ^{1}H NMR spectrum of compound A19 (400 MHz, DMSO).

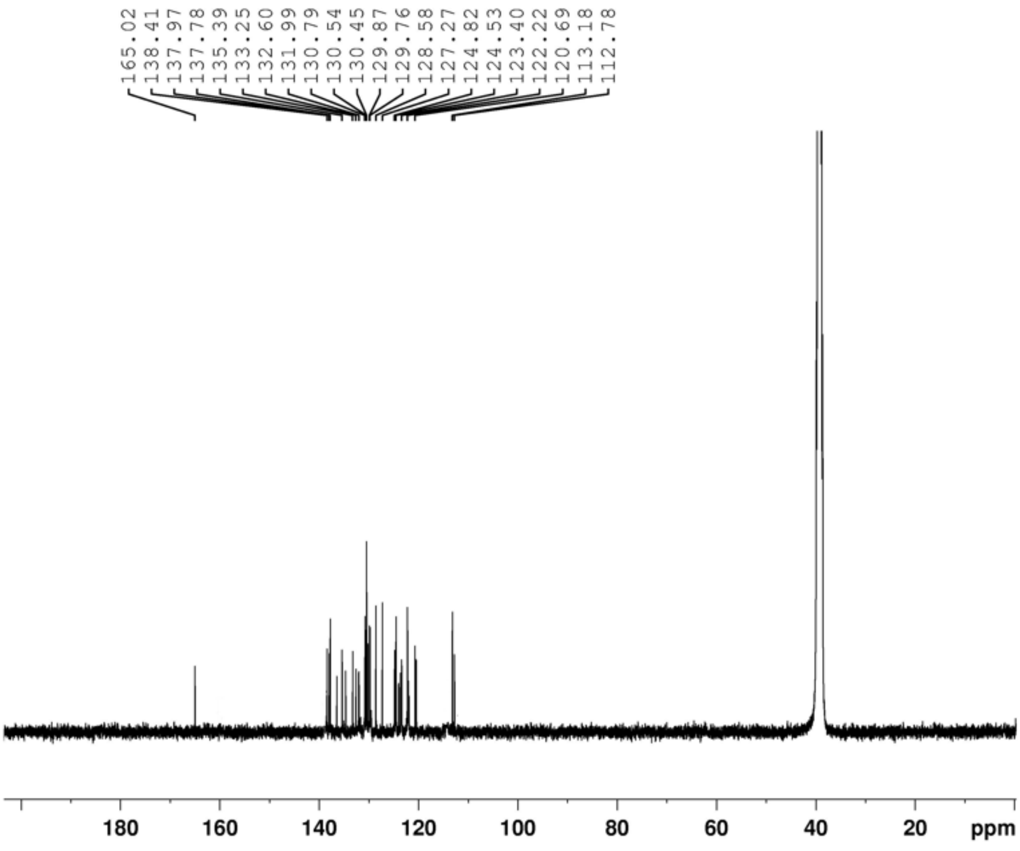

Figure S71. ^{13}C NMR spectrum of compound A19 (101 MHz, DMSO).

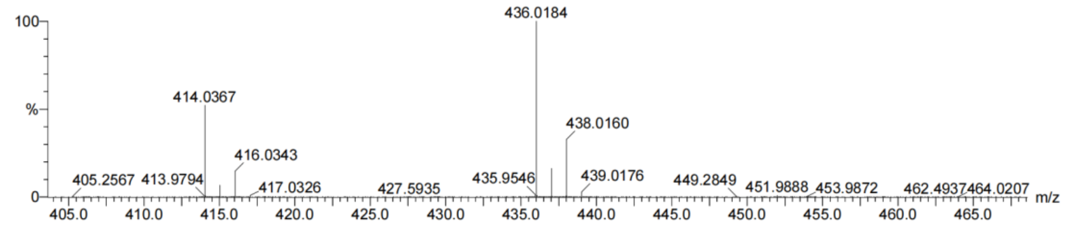

Figure S72. High resolution mass spectrum of compound A19.

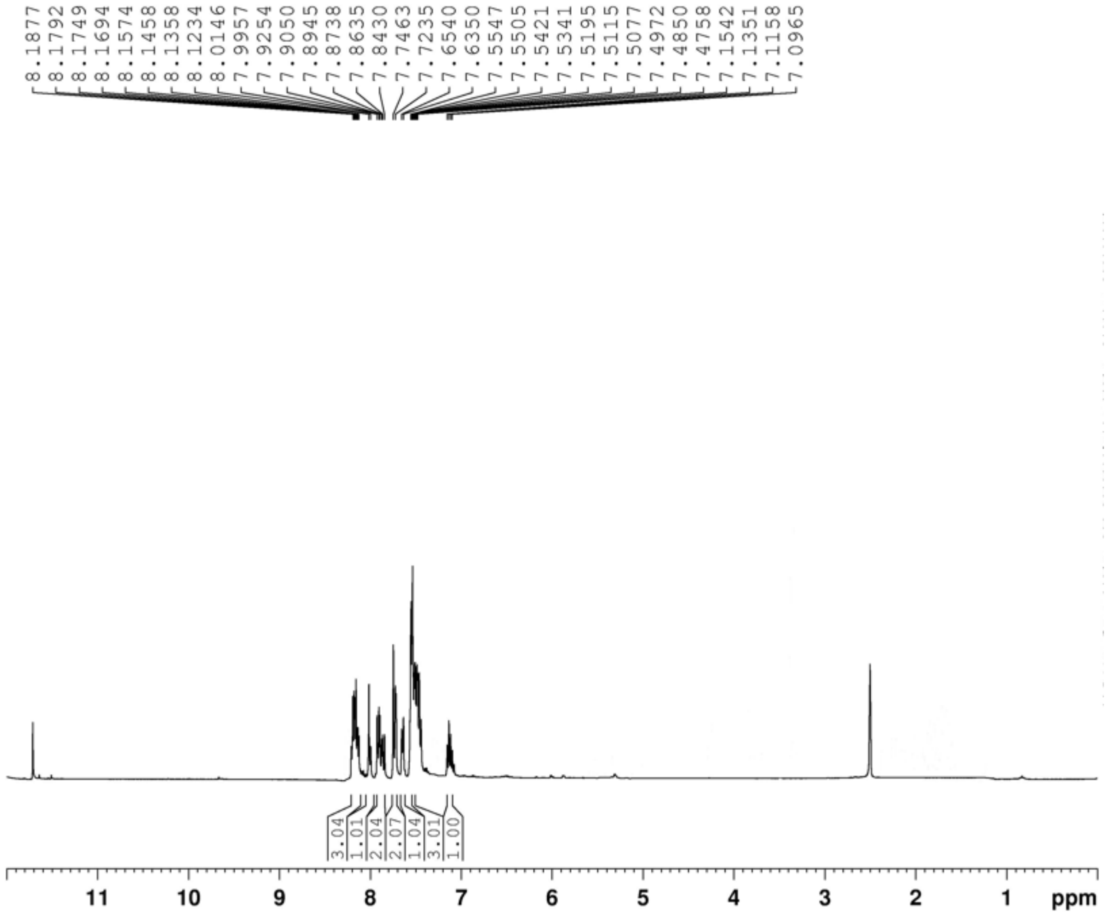

Figure S73. ^{1}H NMR spectrum of compound A20 (400 MHz, DMSO).

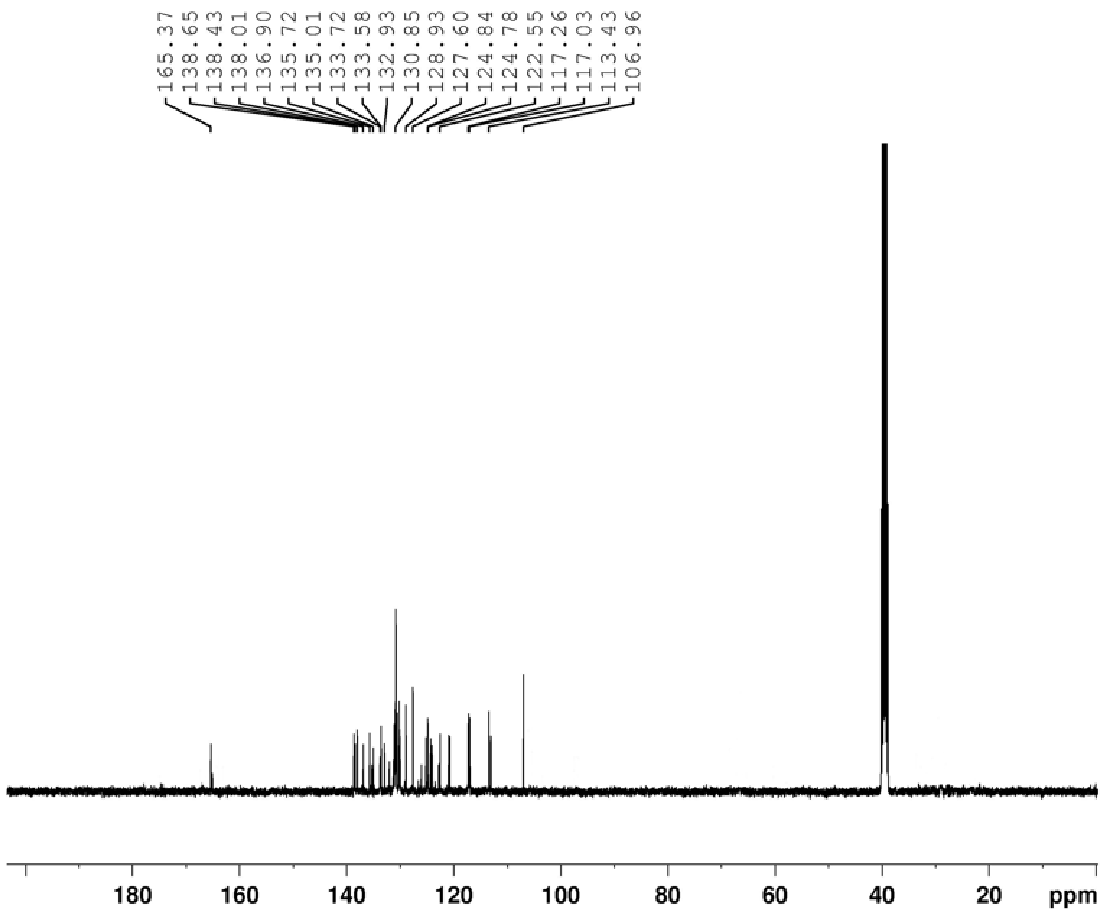

Figure S74. ^{13}C NMR spectrum of compound A20 (101 MHz, DMSO).

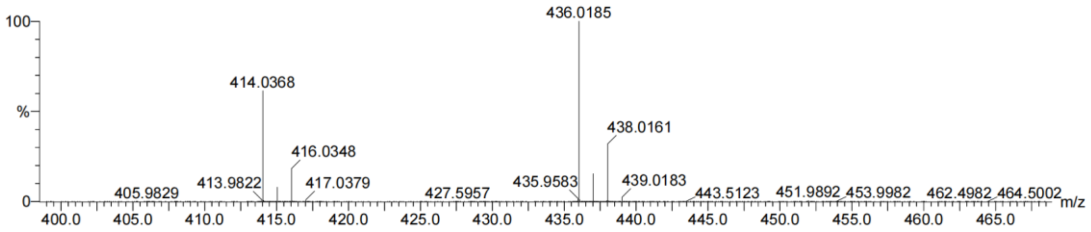

Figure S75. High resolution mass spectrum of compound A20.

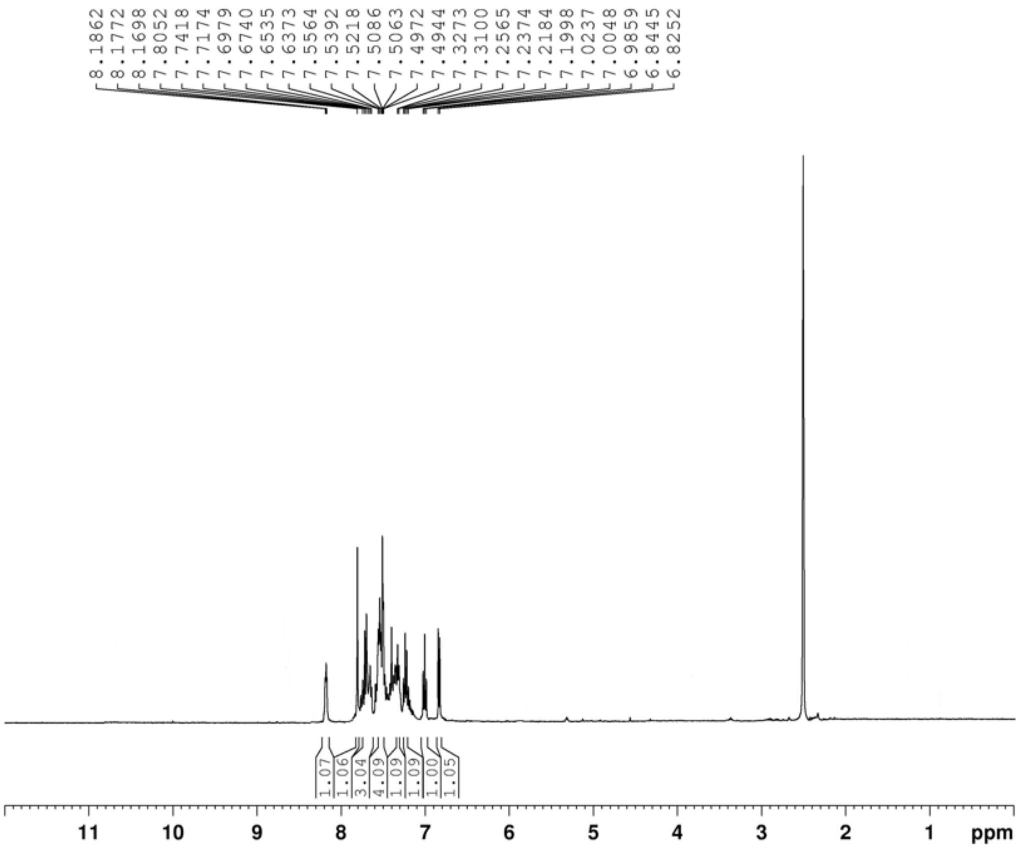

Figure S76. ^{1}H NMR spectrum of compound A21 (400 MHz, DMSO).

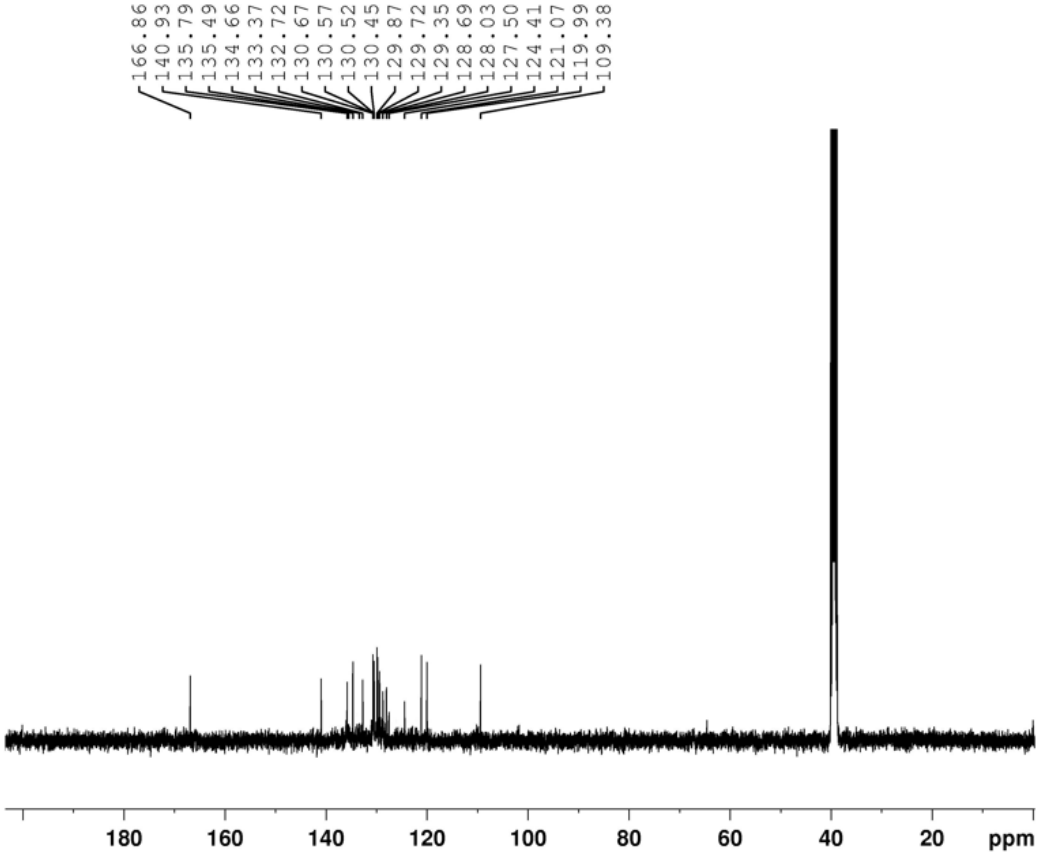

Figure S77. ^{13}C NMR spectrum of compound A21 (101 MHz, DMSO).

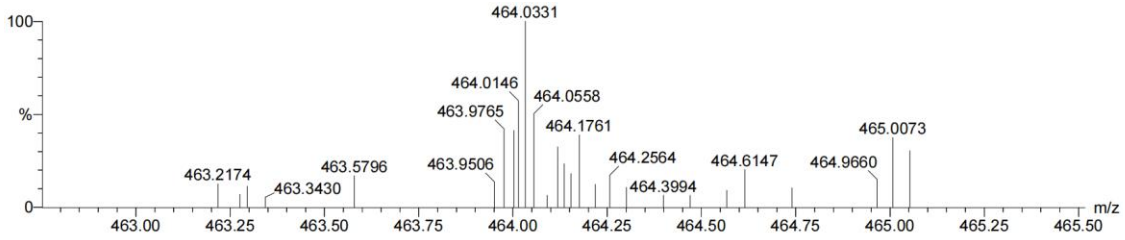

Figure S78. High resolution mass spectrum of compound A21.

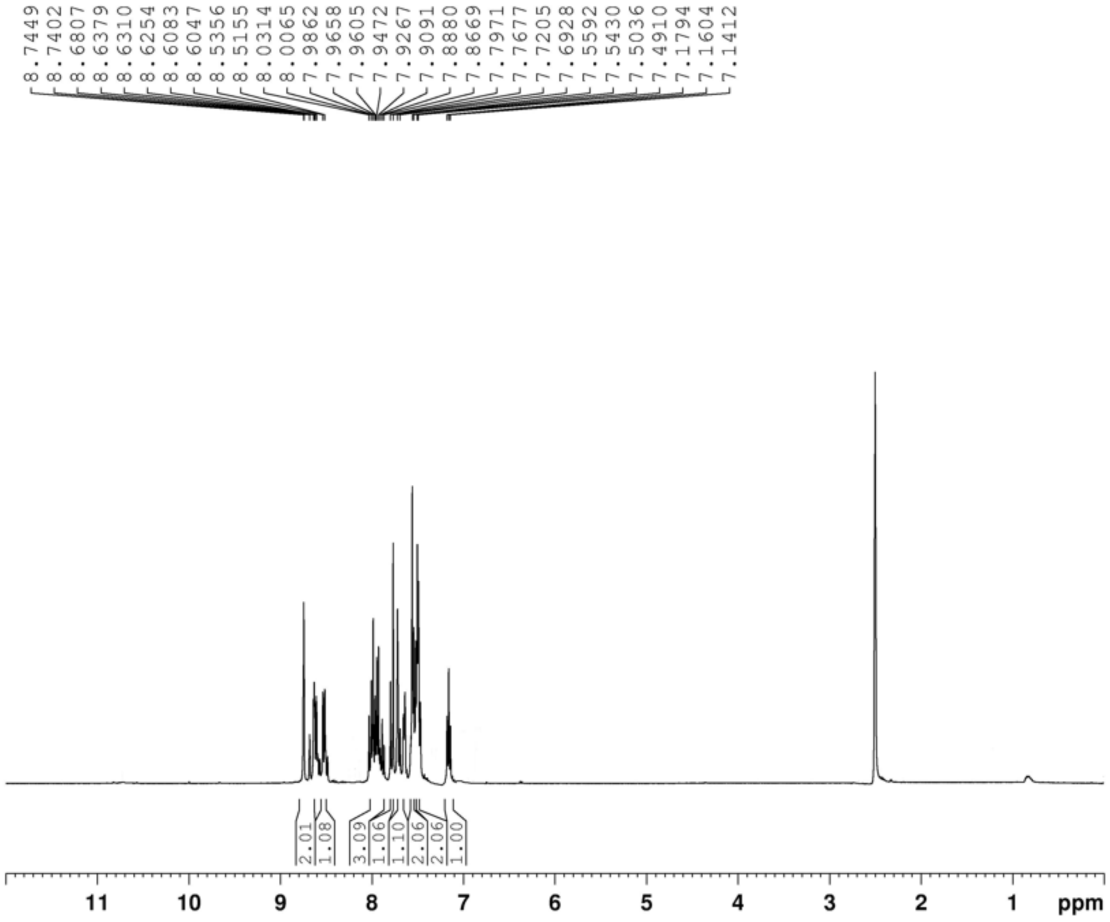

Figure S79. ^{1}H NMR spectrum of compound A22 (400 MHz, DMSO).

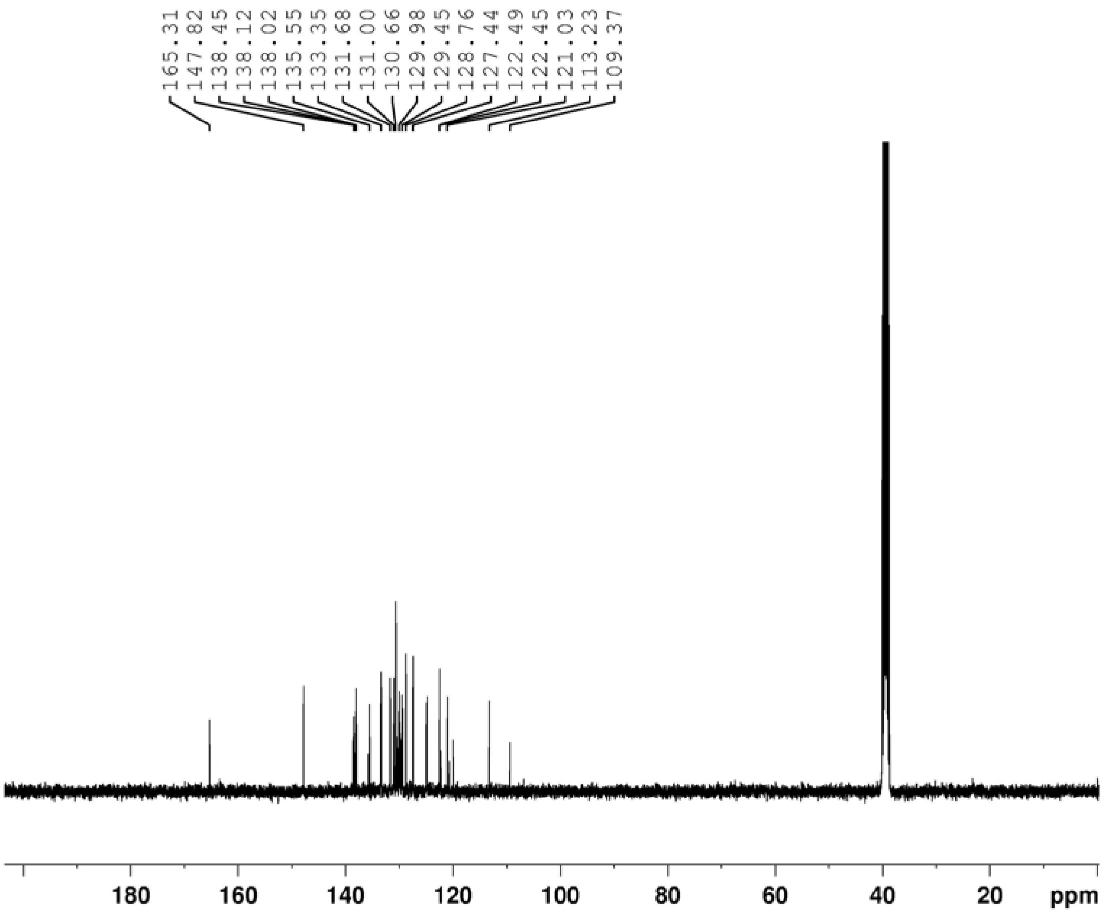

Figure S80. ^{13}C NMR spectrum of compound A22 (101 MHz, DMSO).

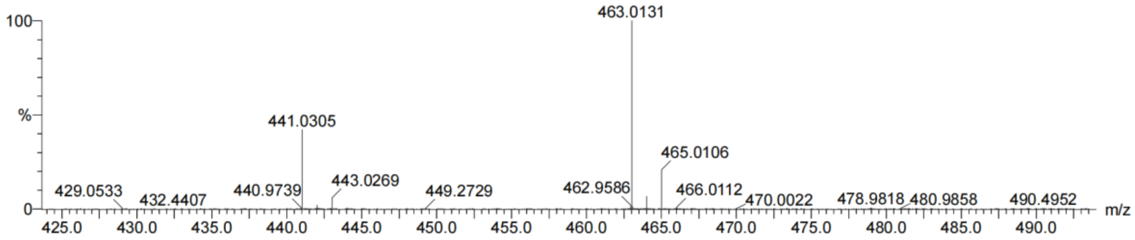

Figure S81. High resolution mass spectrum of compound A22.

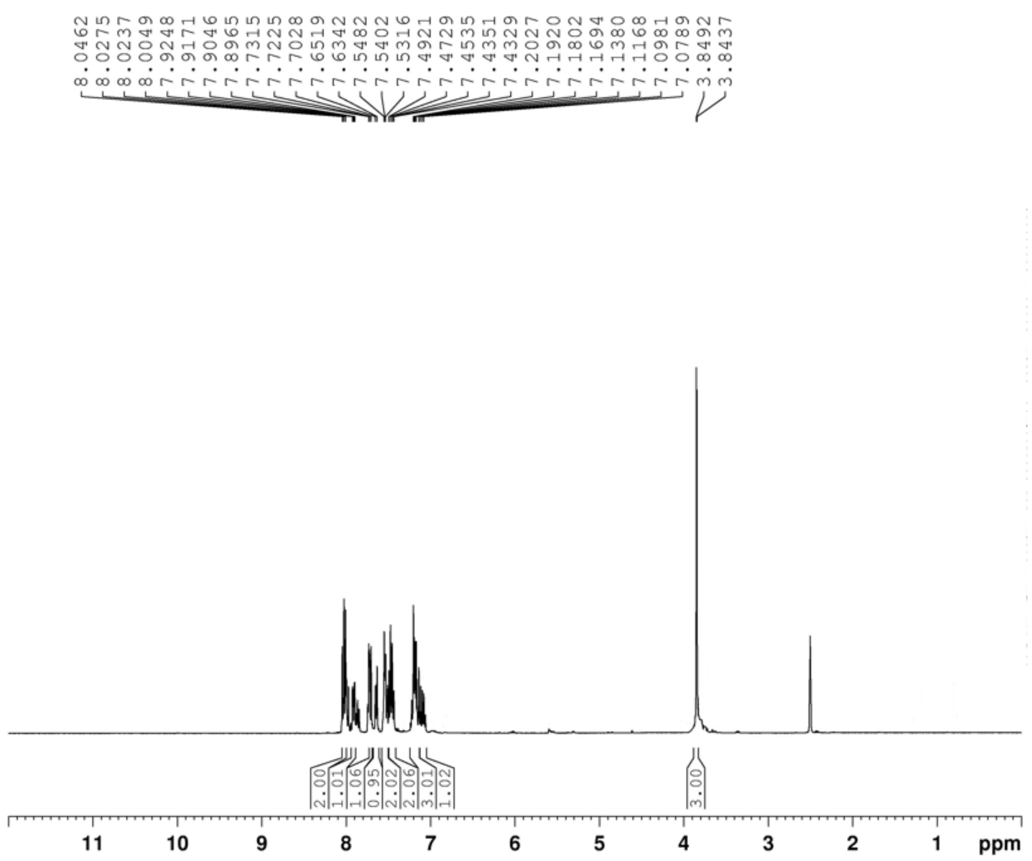

Figure S82. ^{1}H NMR spectrum of compound A23 (400 MHz, DMSO).

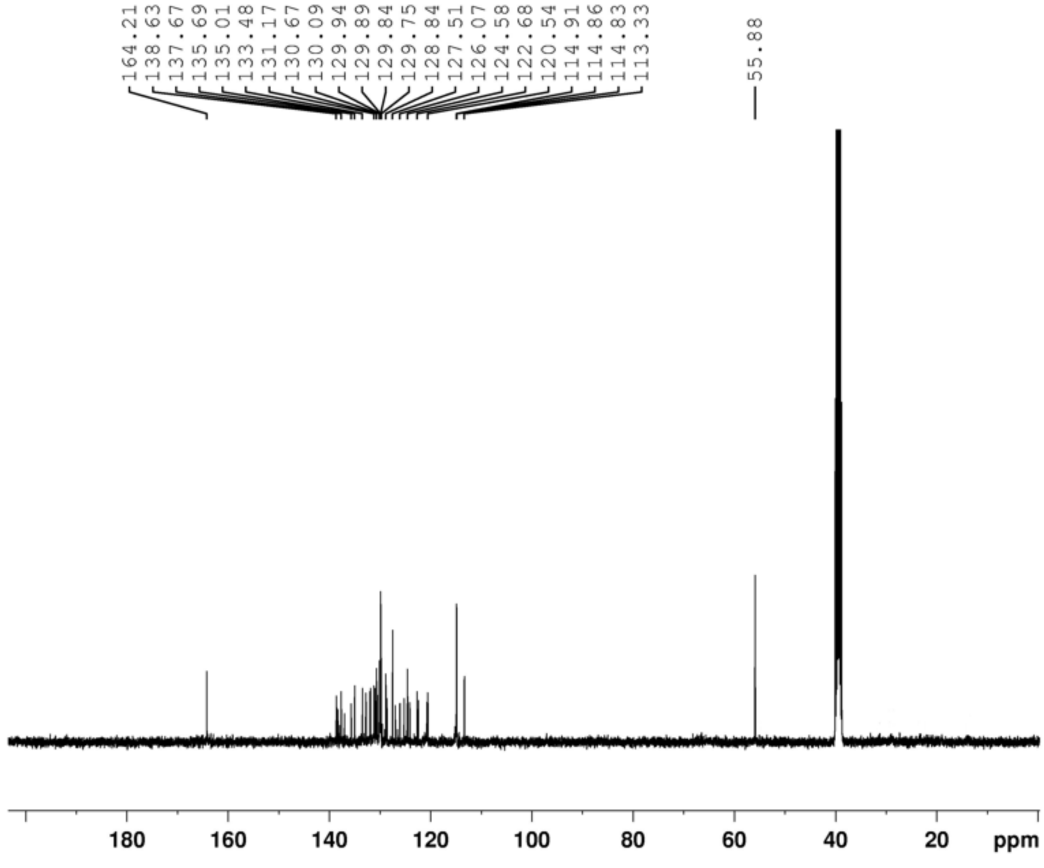

Figure S83. ^{13}C NMR spectrum of compound A23 (101 MHz, DMSO).

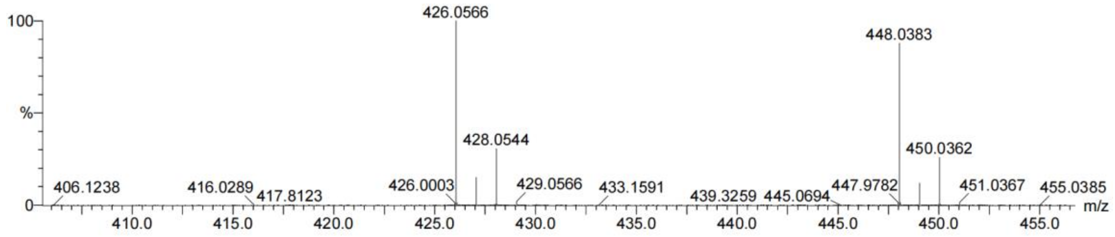

Figure S84. High resolution mass spectrum of compound A23.

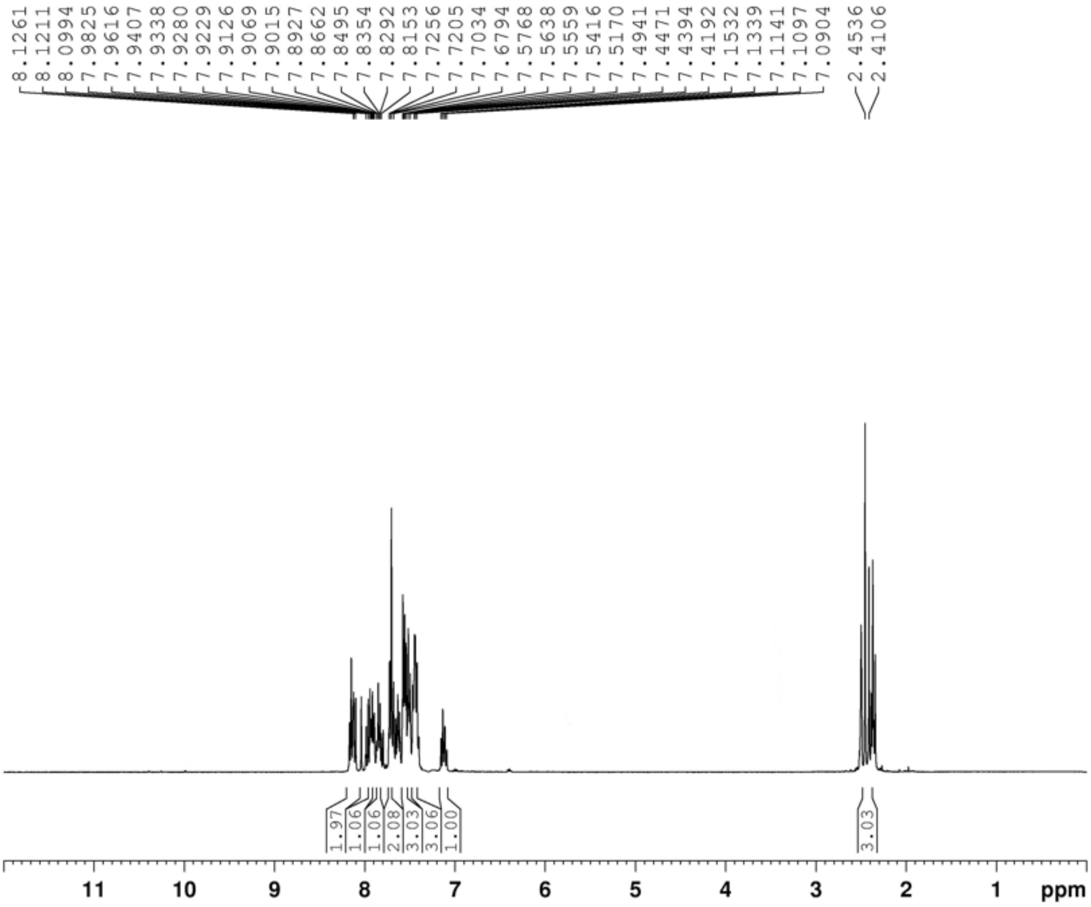

Figure S85. ^{1}H NMR spectrum of compound A24 (400 MHz, DMSO).

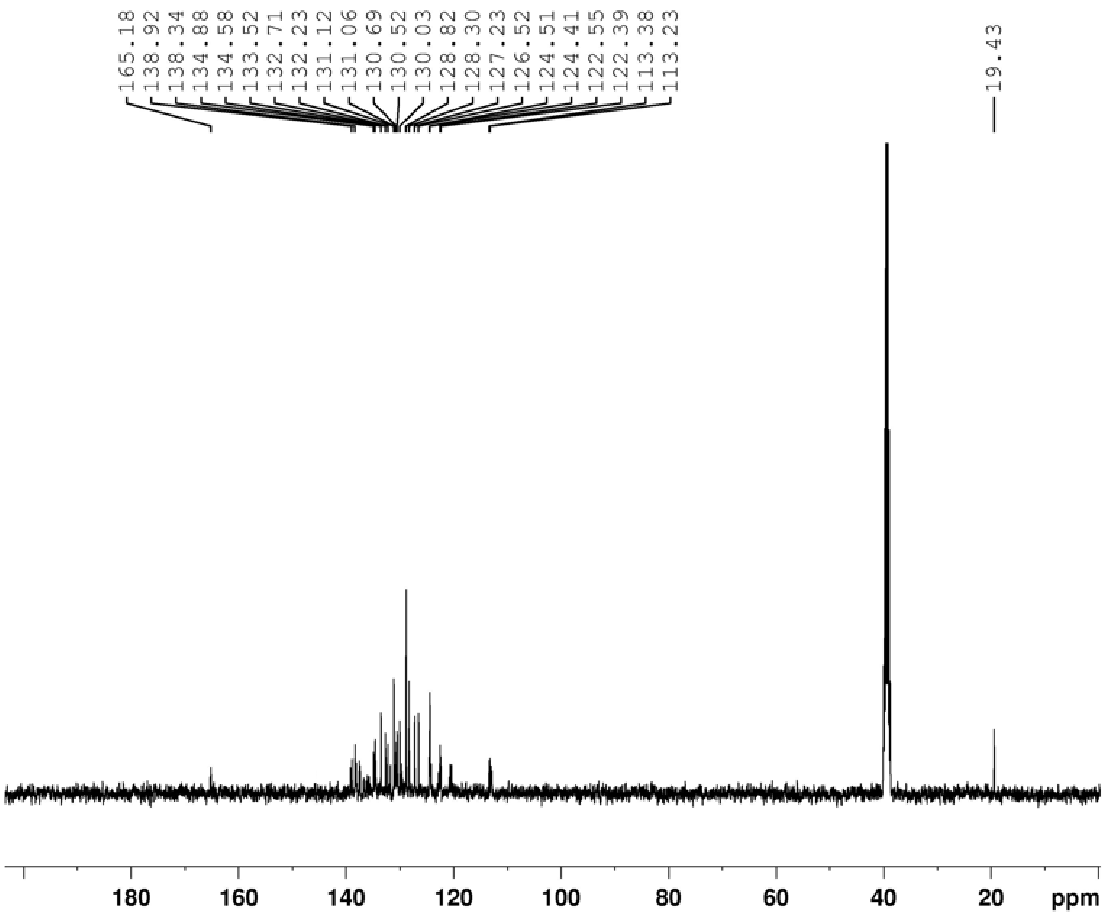

Figure S86. ^{13}C NMR spectrum of compound A24 (101 MHz, DMSO).

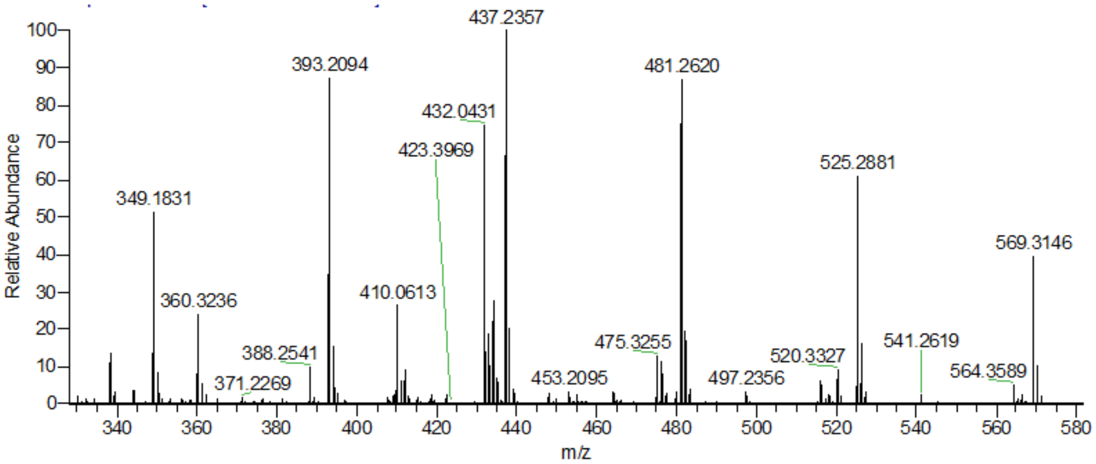

Figure S87. High resolution mass spectrum of compound A24.

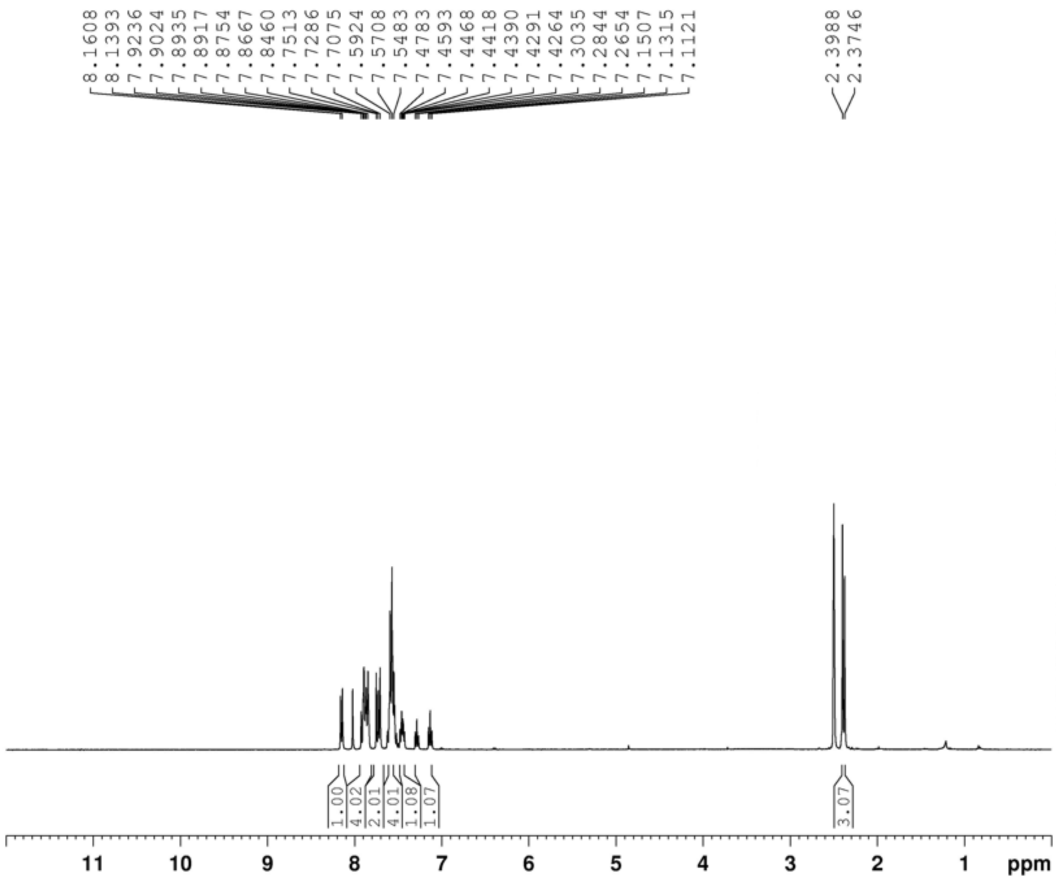

Figure S88. ^{1}H NMR spectrum of compound A25 (400 MHz, DMSO).

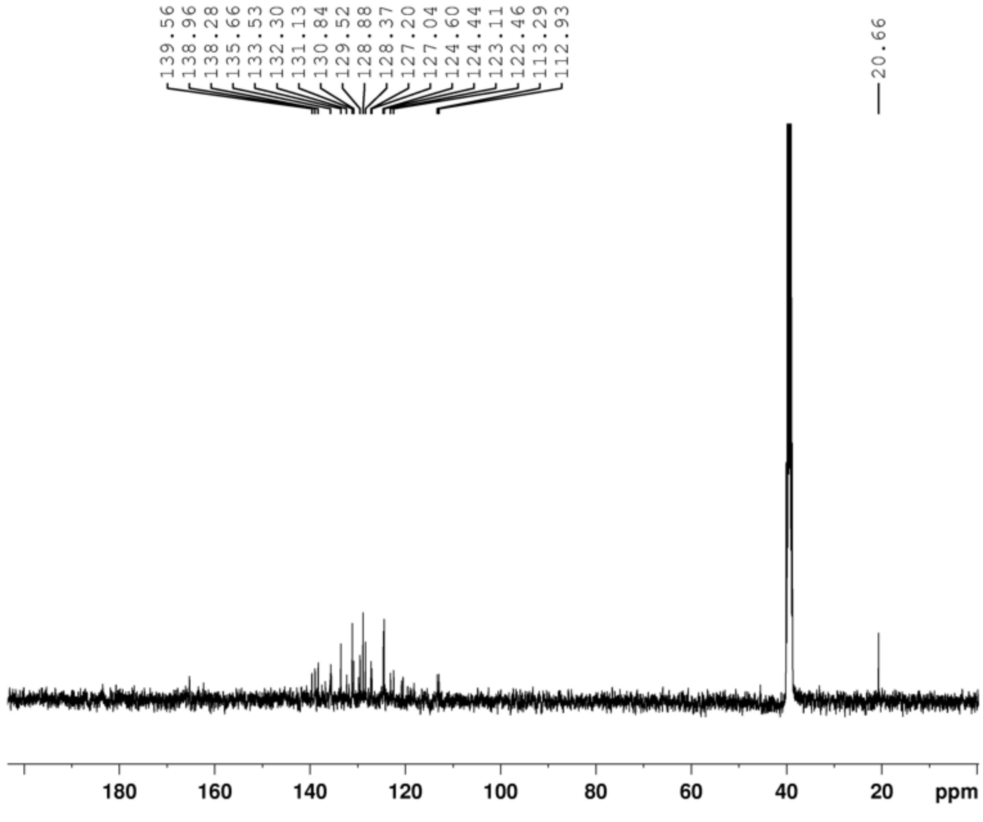

Figure S89. ^{13}C NMR spectrum of compound A25 (101 MHz, DMSO).

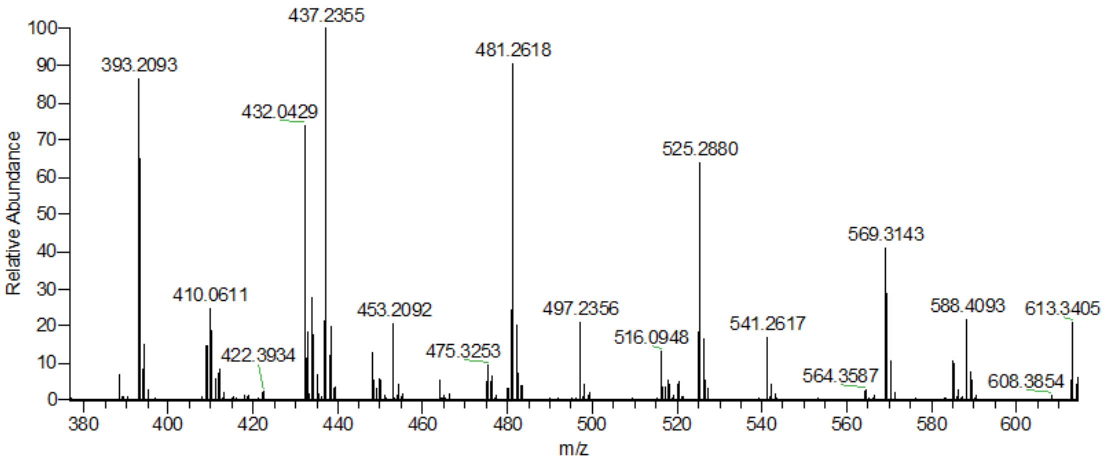

Figure S90. High resolution mass spectrum of compound A25.

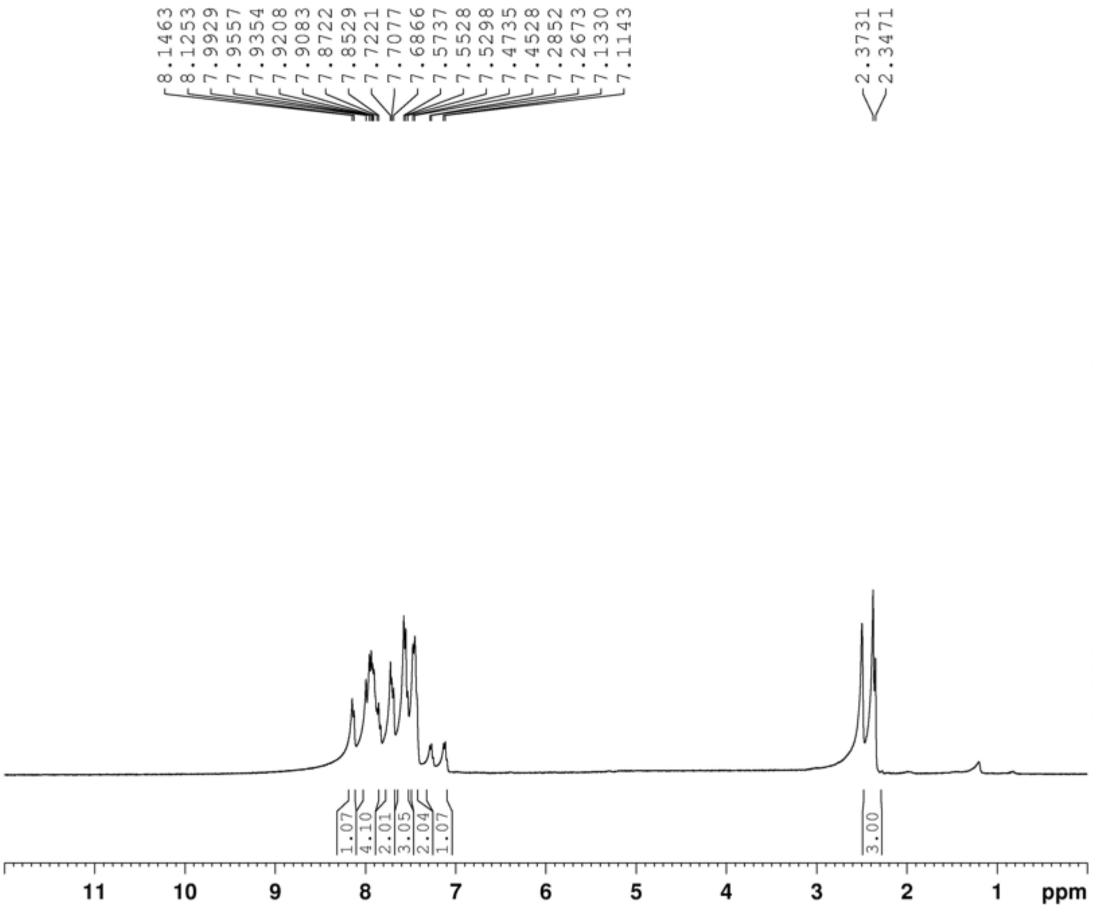

Figure S91. ^{1}H NMR spectrum of compound A26 (400 MHz, DMSO).

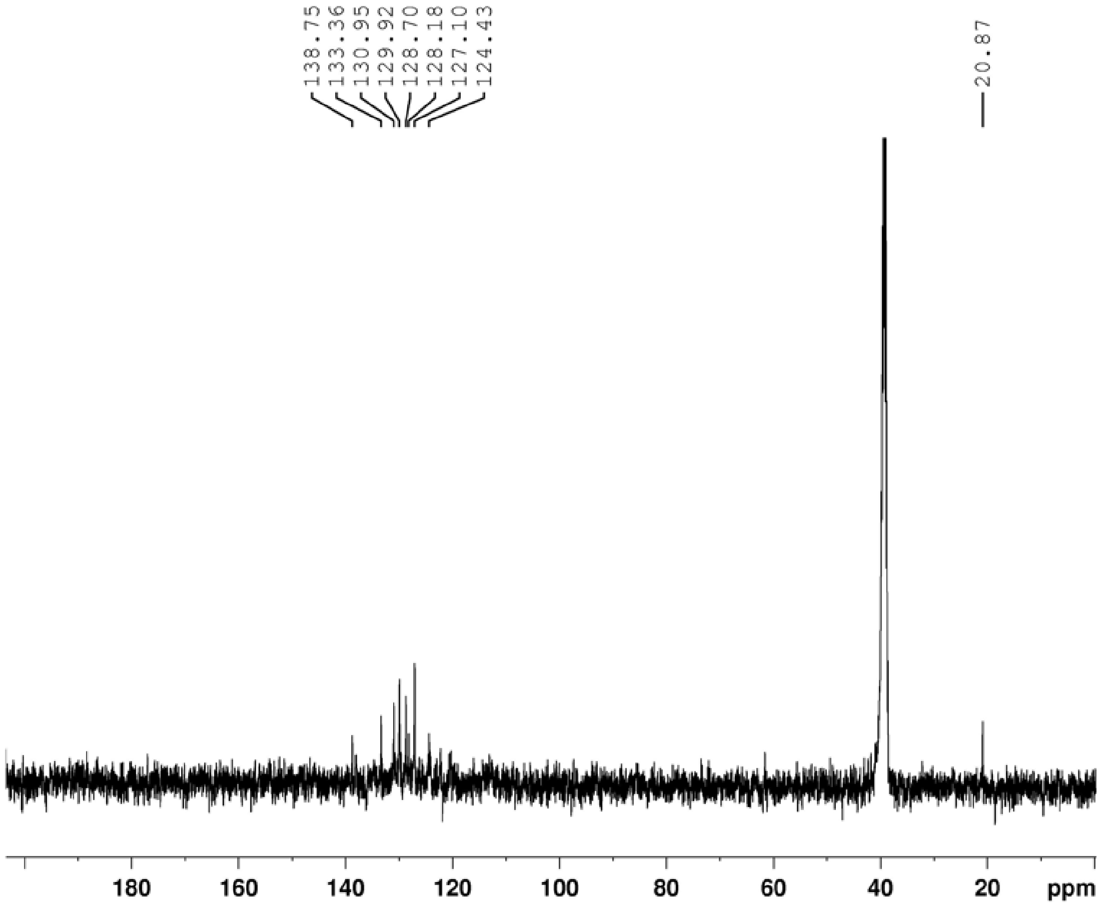

Figure S92. ^{13}C NMR spectrum of compound A26 (101 MHz, DMSO).

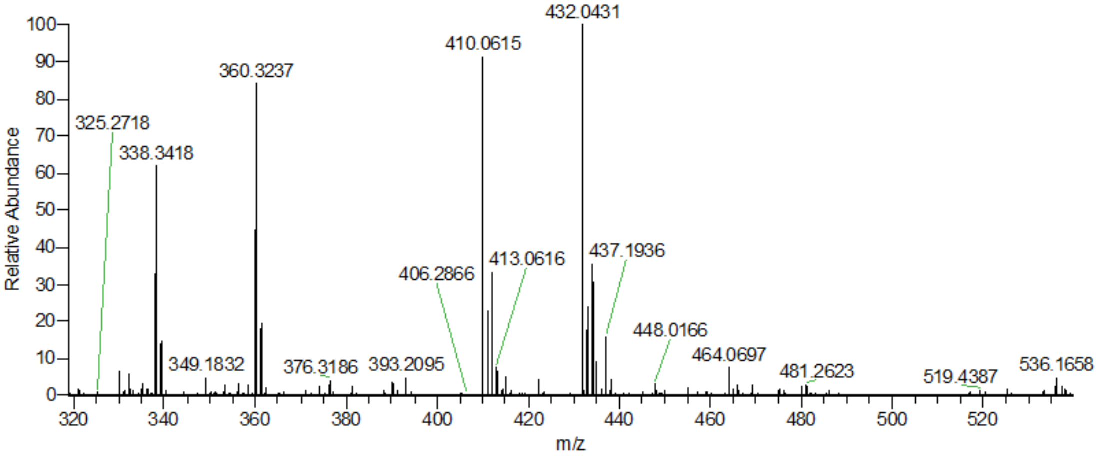

Figure S93. High resolution mass spectrum of compound A26.

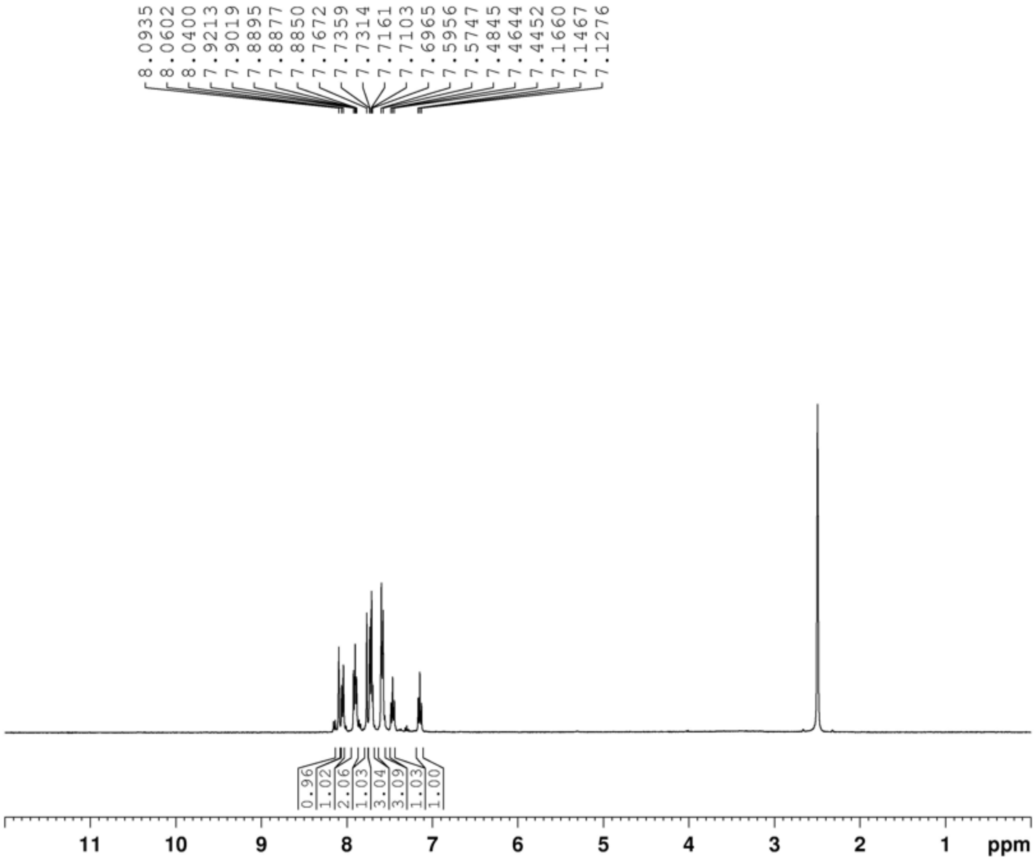

Figure S94. ^{1}H NMR spectrum of compound A27 (400 MHz, DMSO).

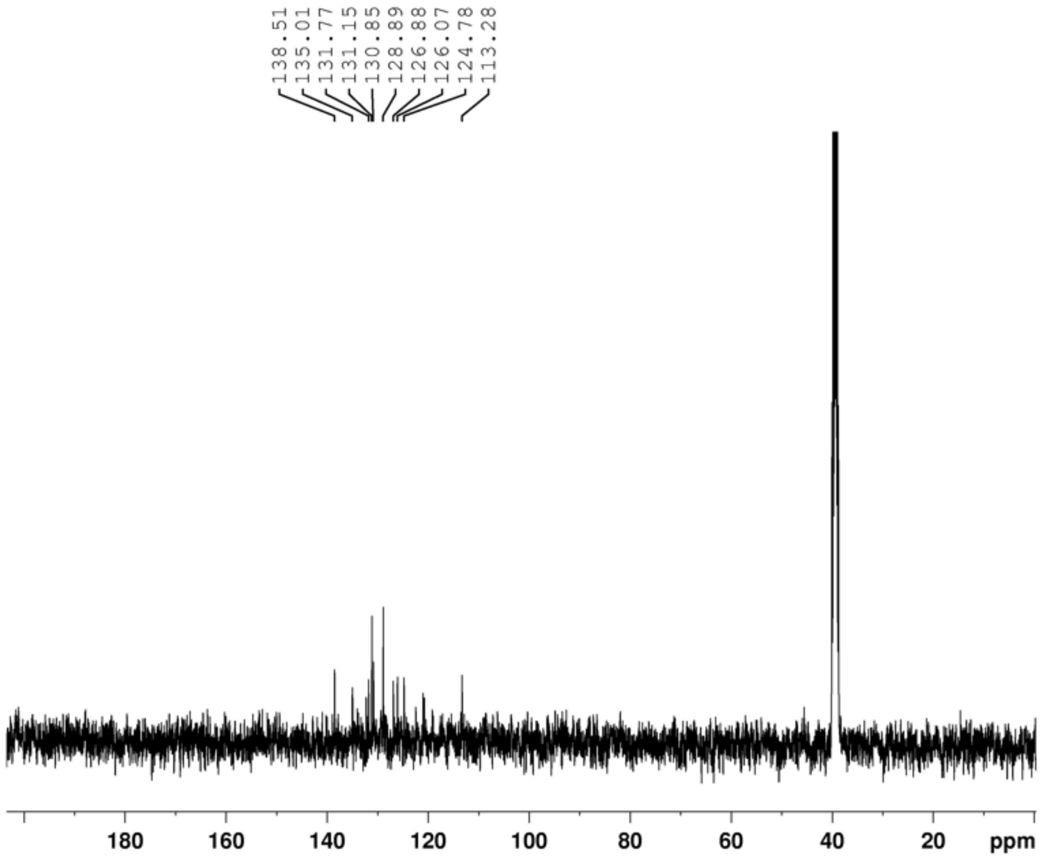

Figure S95. ^{13}C NMR spectrum of compound A27 (101 MHz, DMSO).

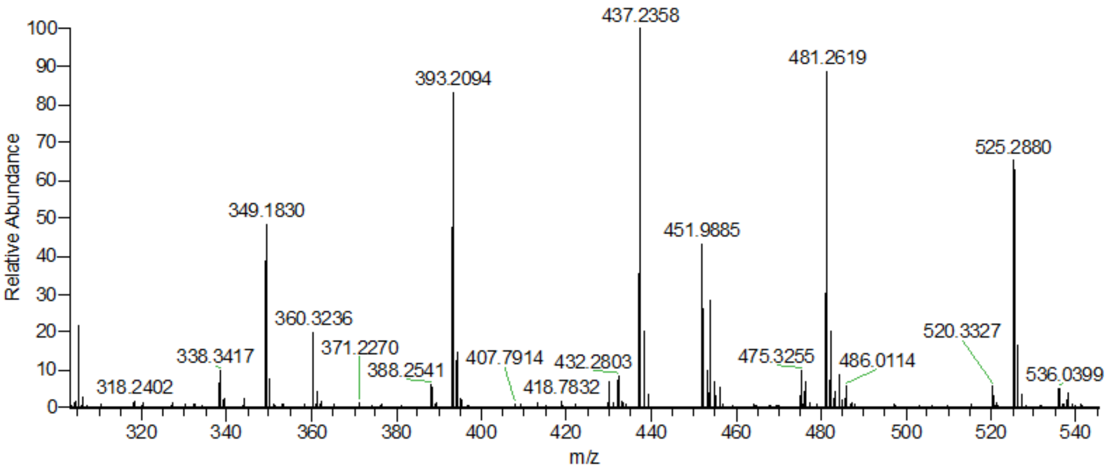

Figure S96. High resolution mass spectrum of compound A27.

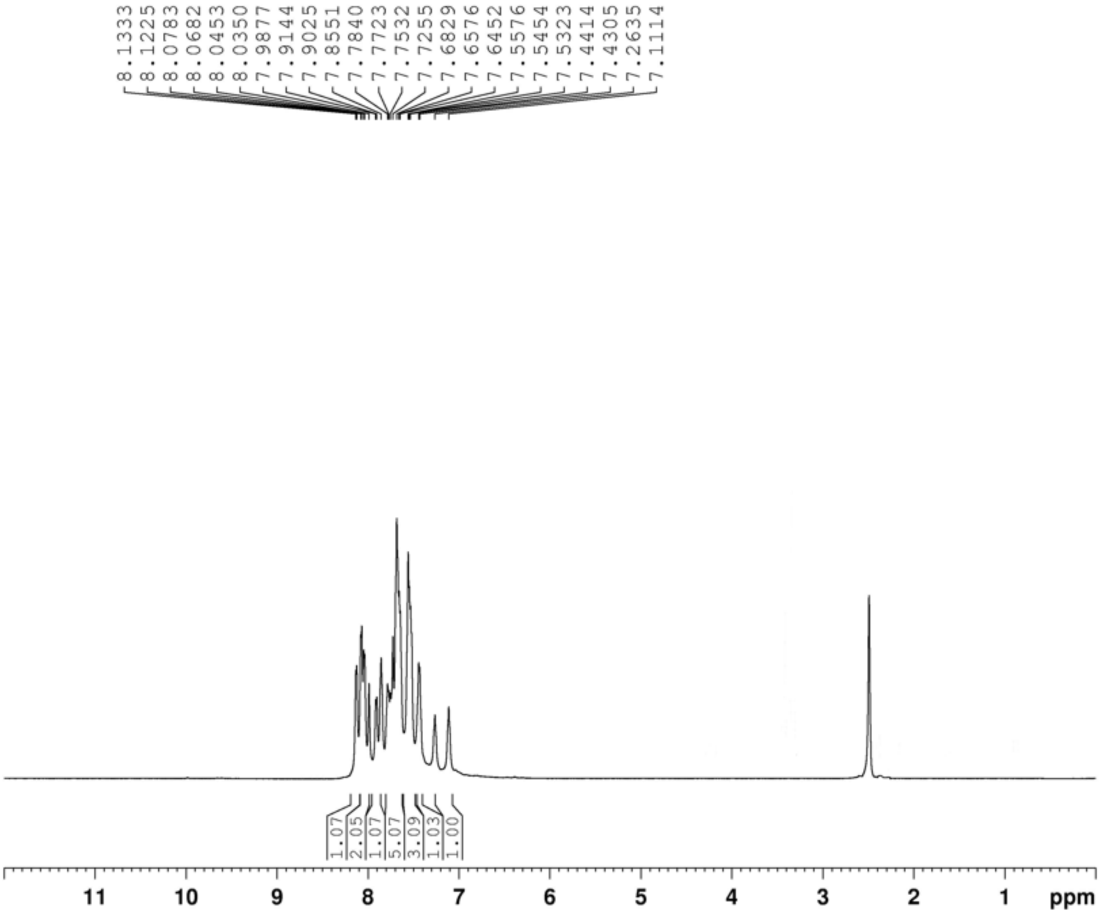

Figure S97. ^{1}H NMR spectrum of compound A28 (600 MHz, DMSO).

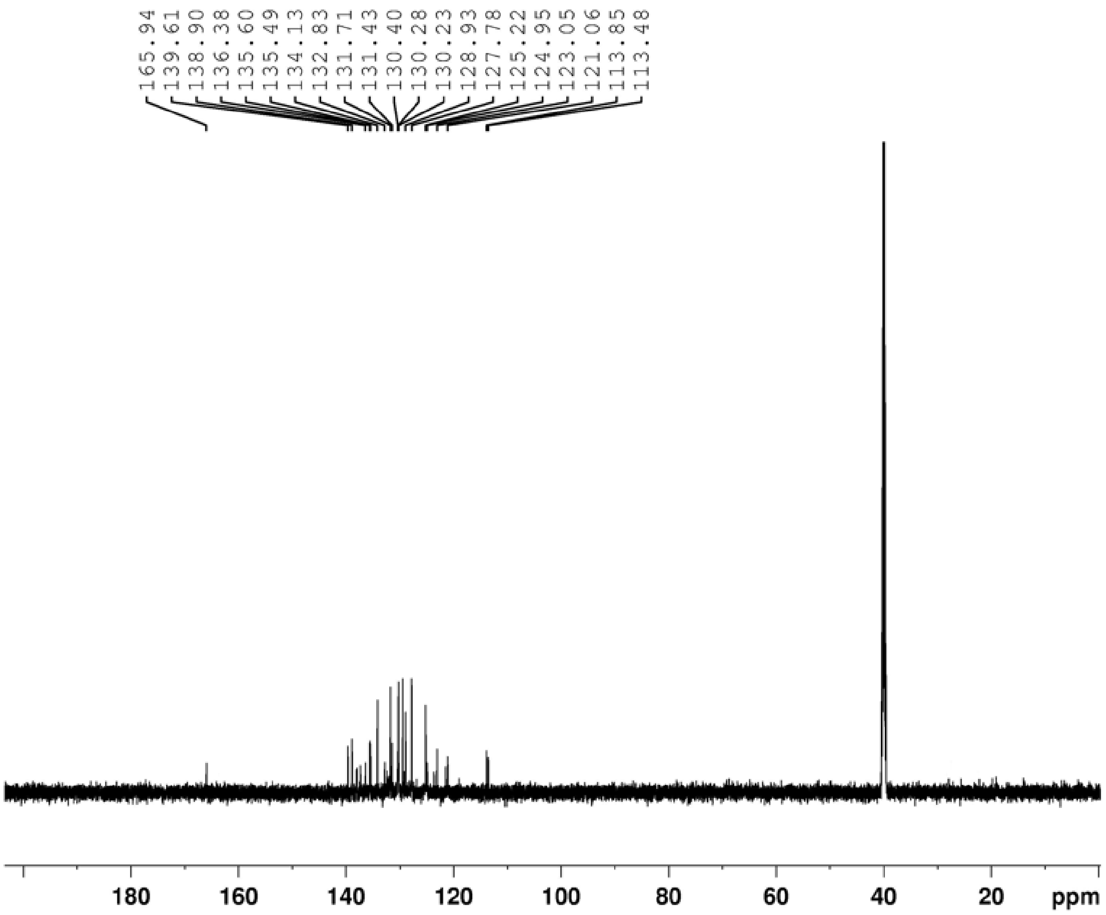

Figure S98. ^{13}C NMR spectrum of compound A28 (151 MHz, DMSO).

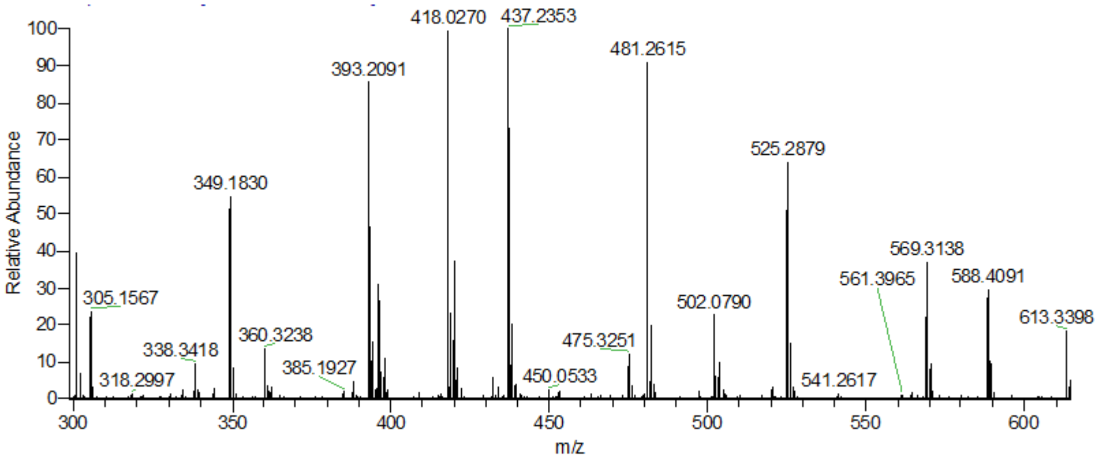

Figure S99. High resolution mass spectrum of compound A28.

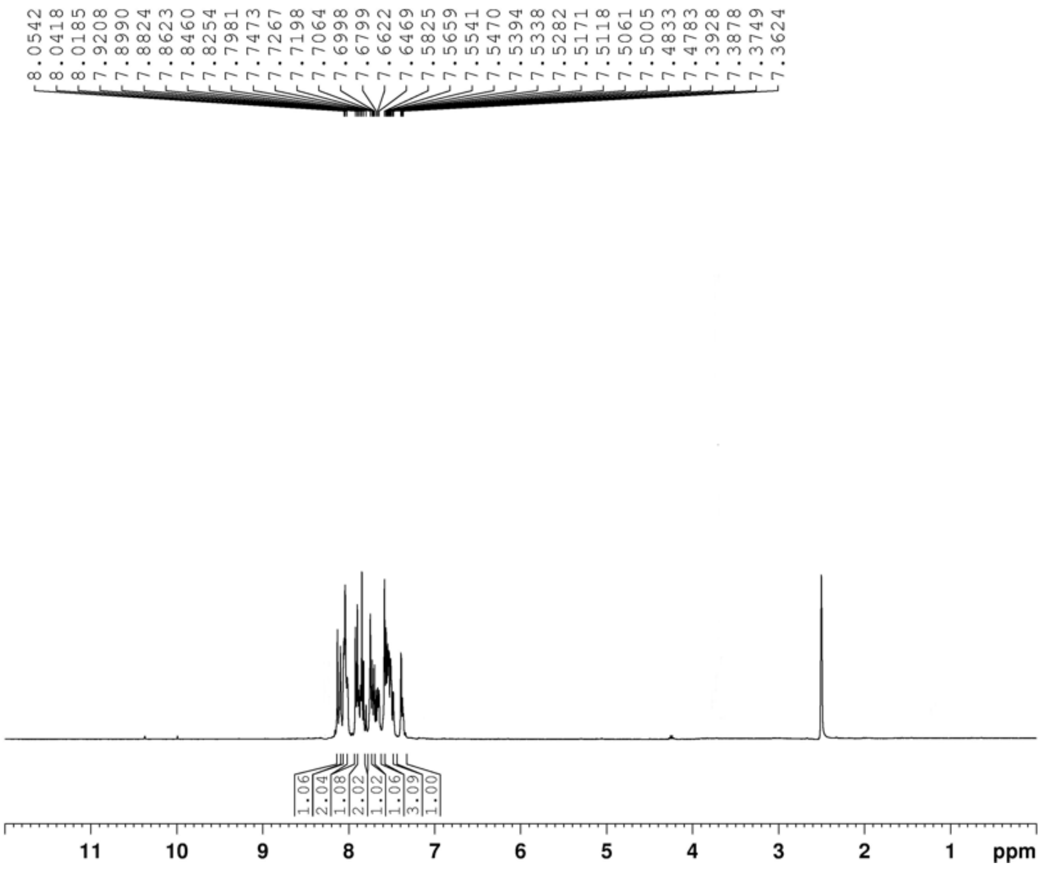

Figure S100. ^{1}H NMR spectrum of compound A29 (400 MHz, DMSO).

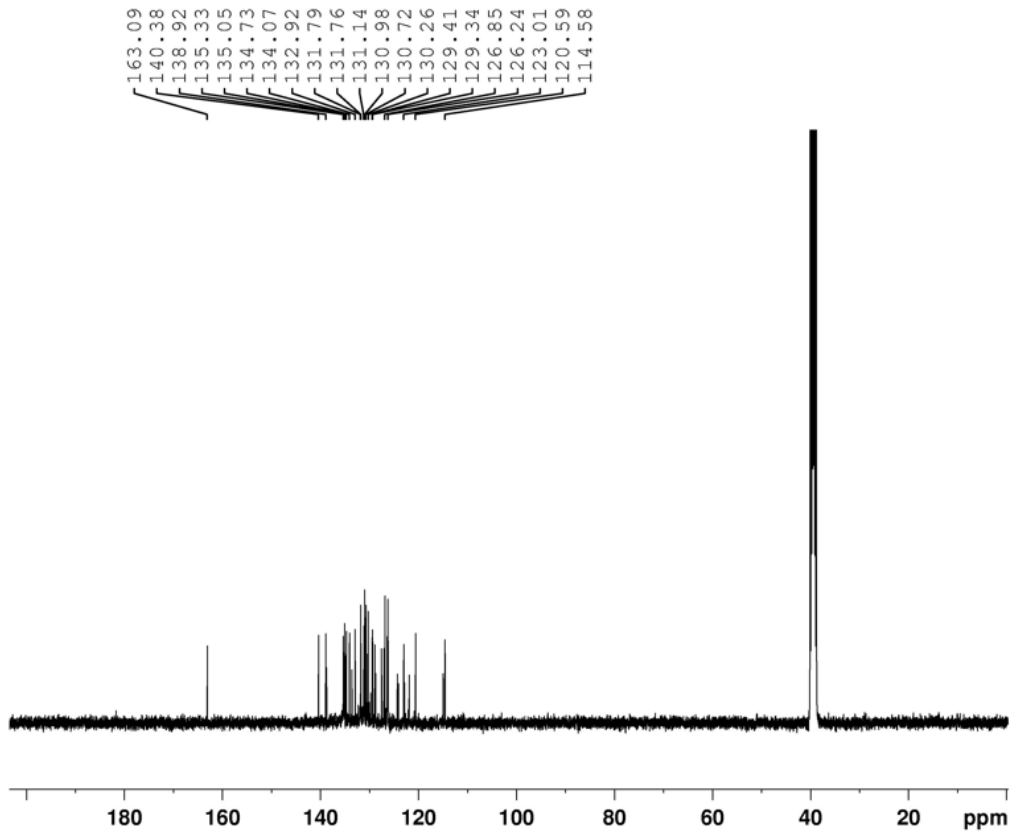

Figure S101. ^{13}C NMR spectrum of compound A29 (101 MHz, DMSO).

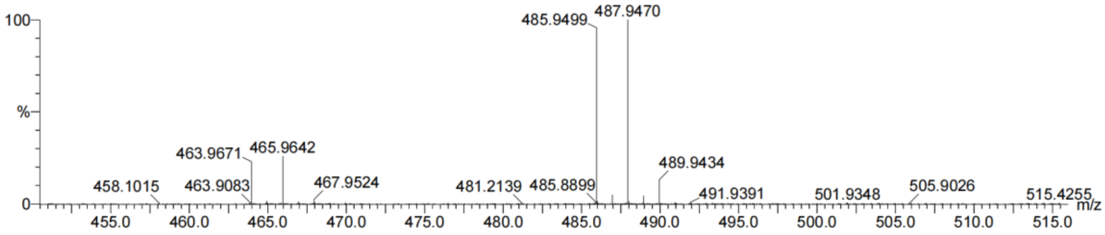

Figure S102. High resolution mass spectrum of compound A29.

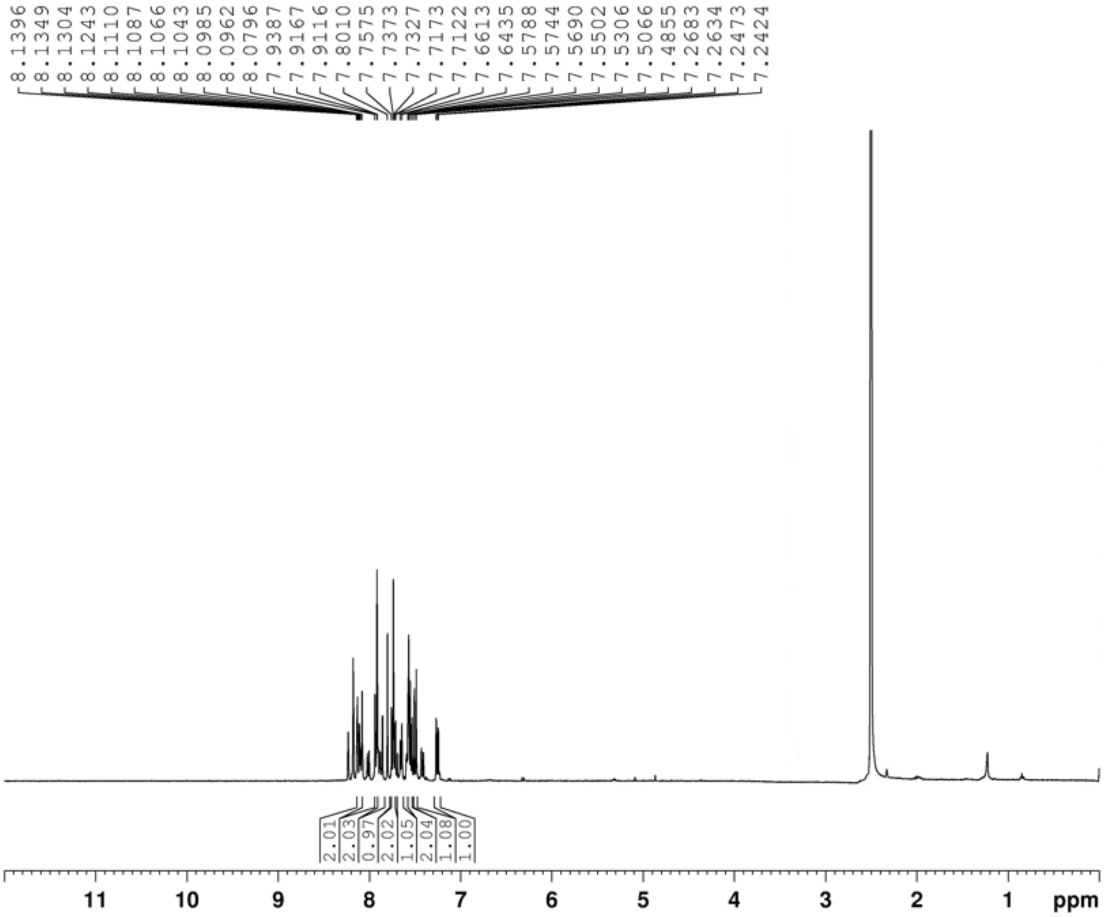

Figure S103. ^{1}H NMR spectrum of compound A30 (400 MHz, DMSO).

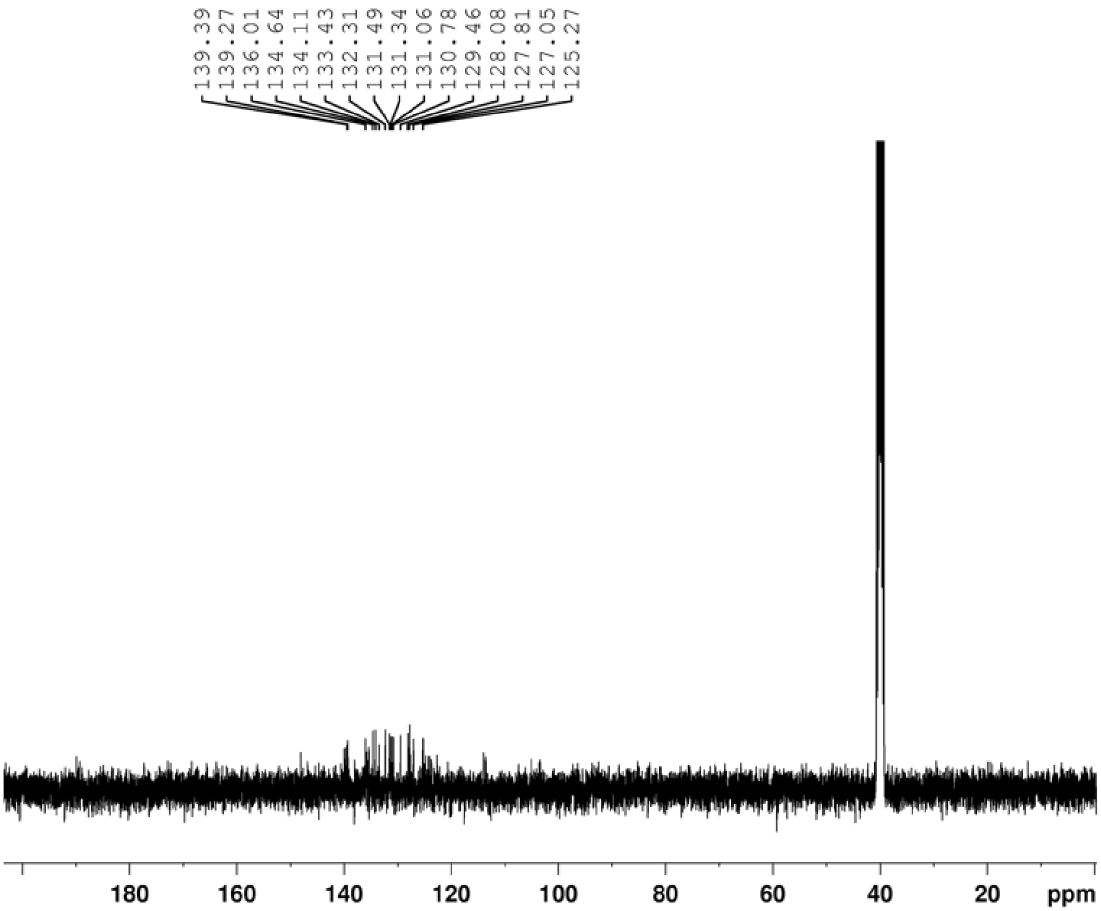

Figure S104. ^{13}C NMR spectrum of compound A30 (101 MHz, DMSO).

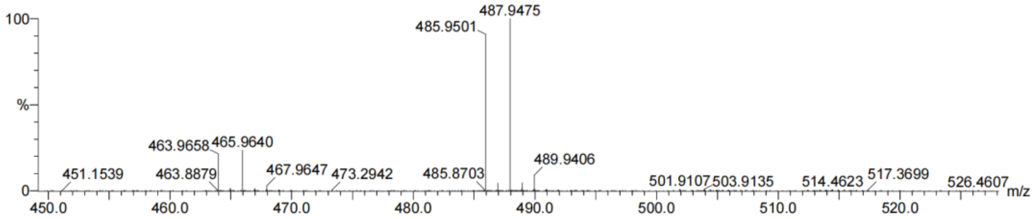

Figure S105. High resolution mass spectrum of compound A30.

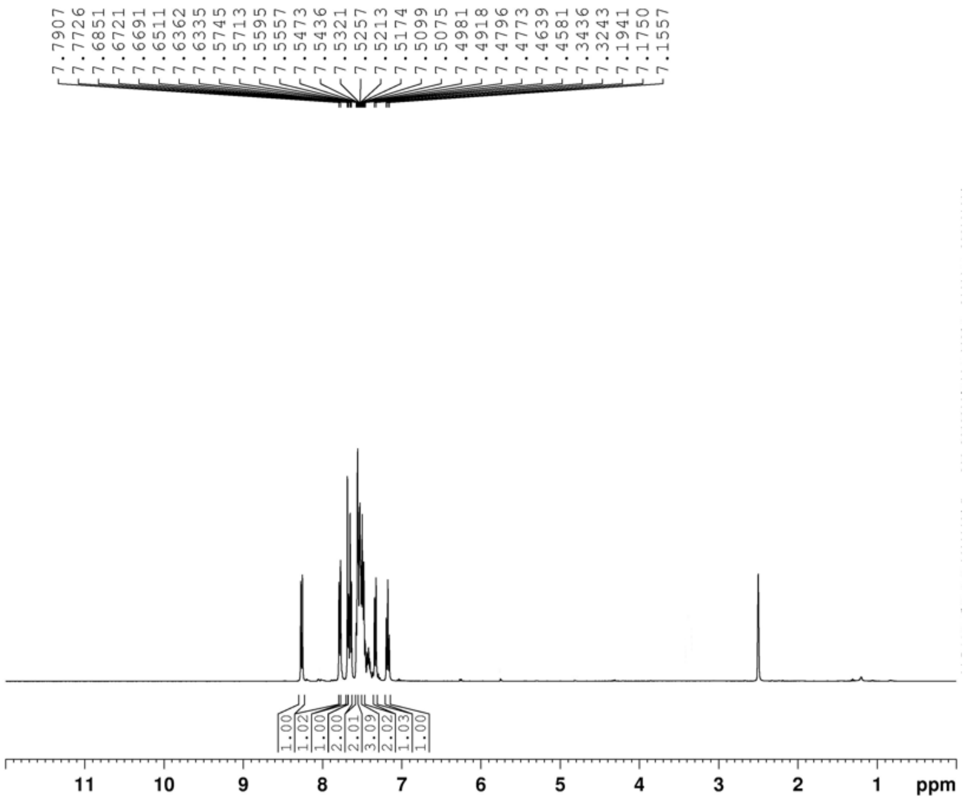

Figure S106. ^{1}H NMR spectrum of compound B1 (400 MHz, DMSO).

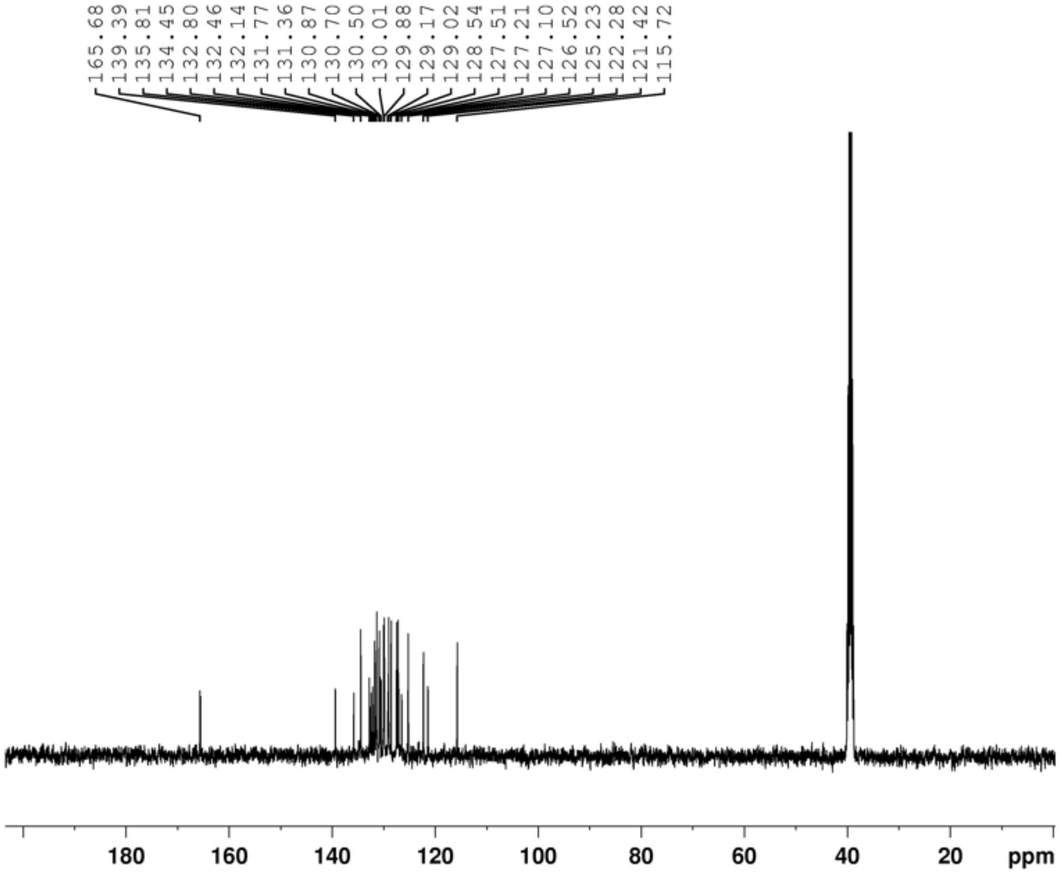

Figure S107. ^{13}C NMR spectrum of compound B1 (101 MHz, DMSO).

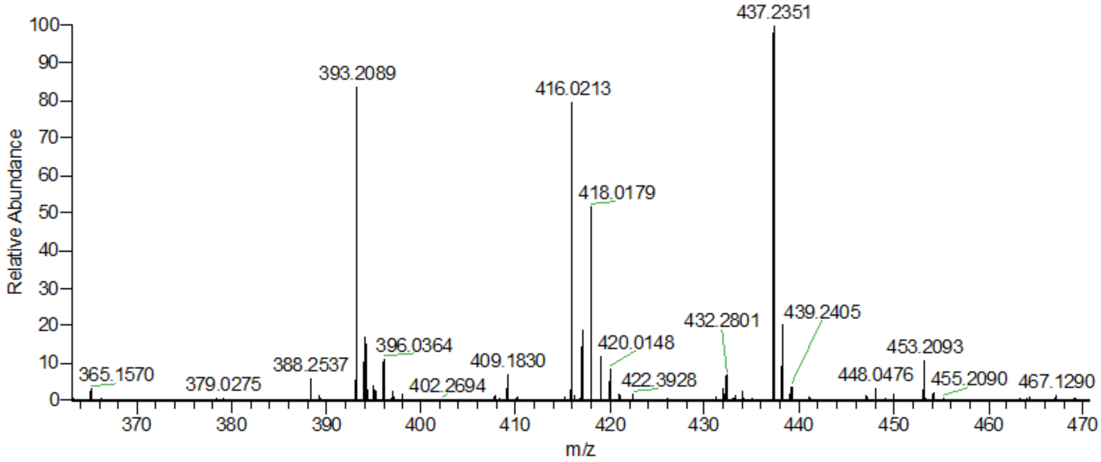

Figure S108. High resolution mass spectrum of compound B1.

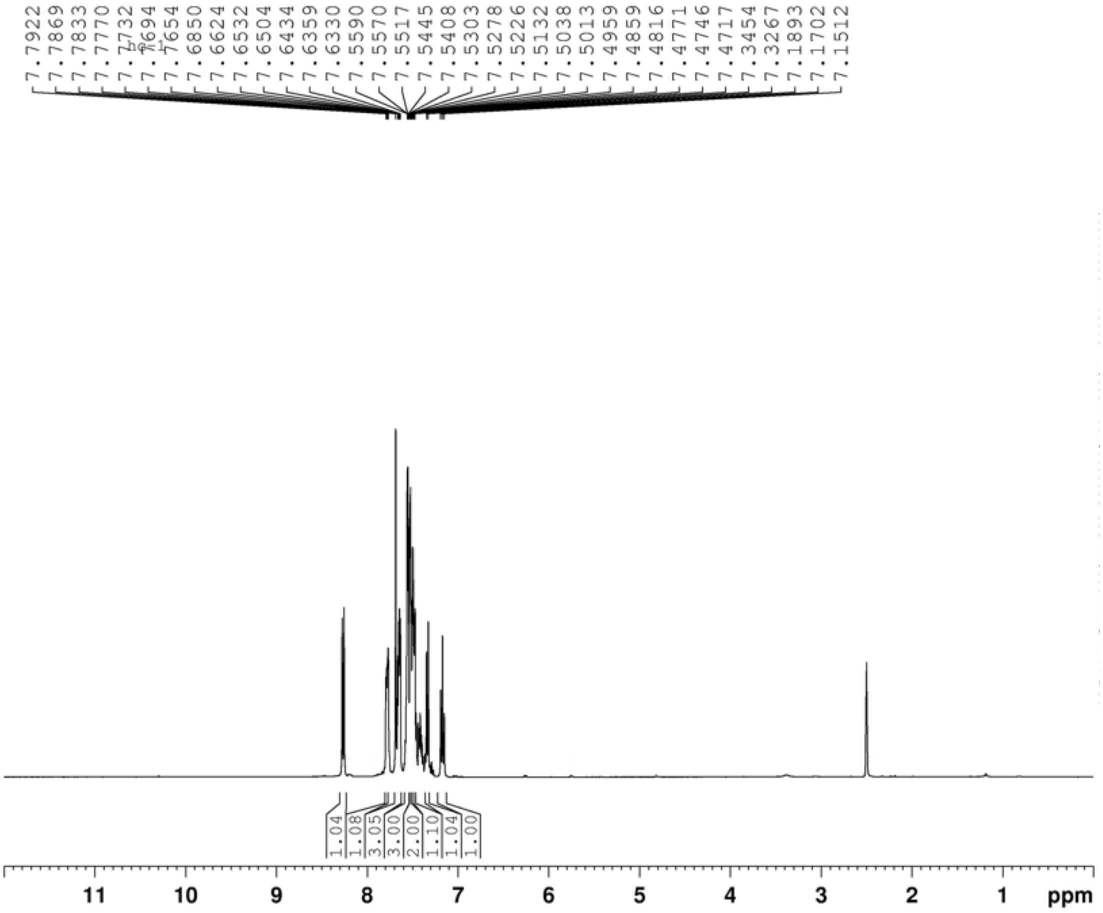

Figure S109. ^{1}H NMR spectrum of compound B2 (400 MHz, DMSO).

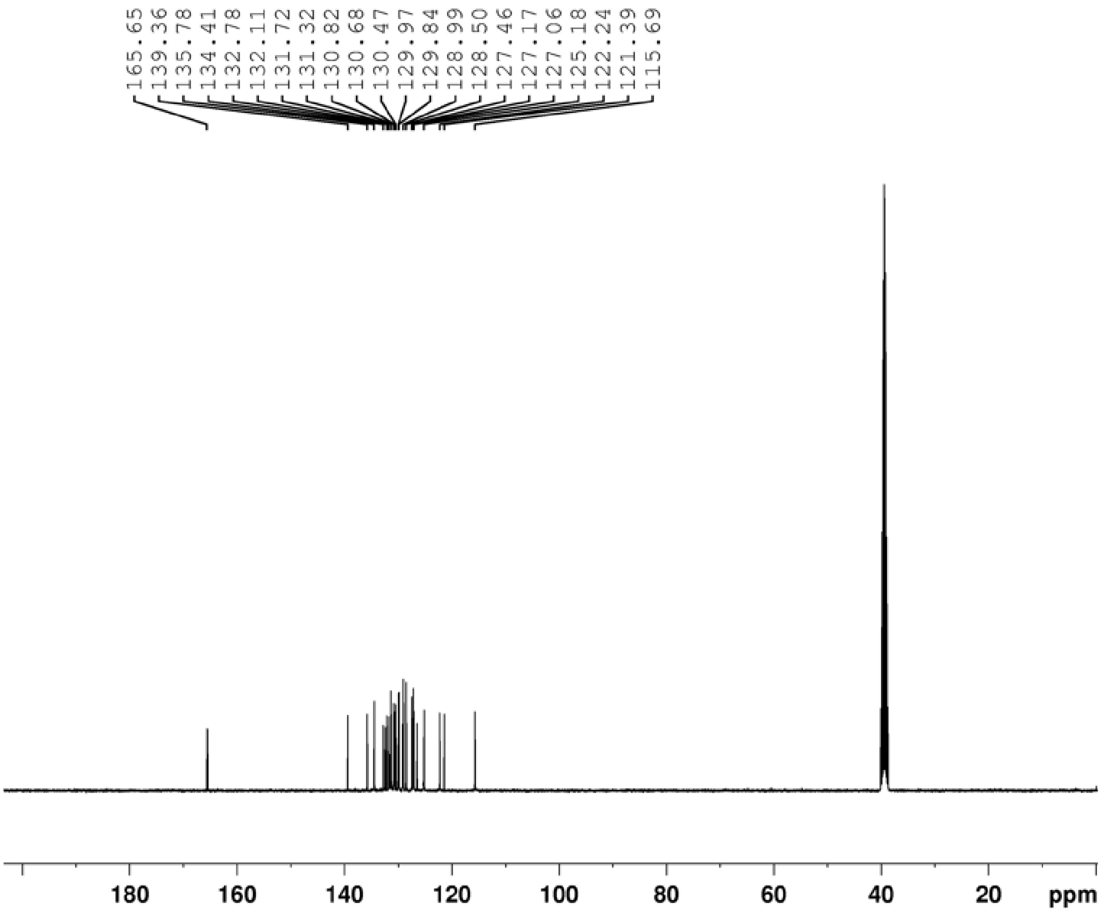

Figure S110. ^{13}C NMR spectrum of compound B2 (101 MHz, DMSO).

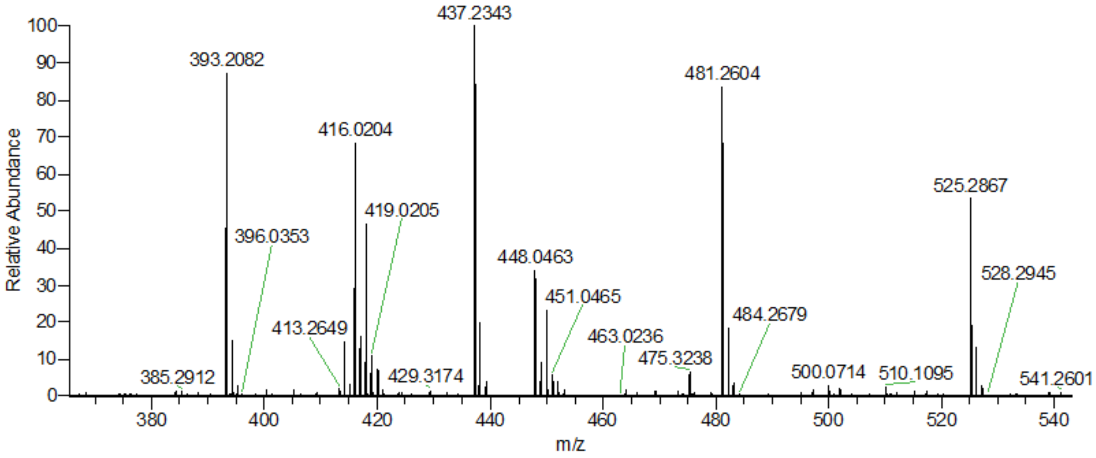

Figure S111. High resolution mass spectrum of compound B2.

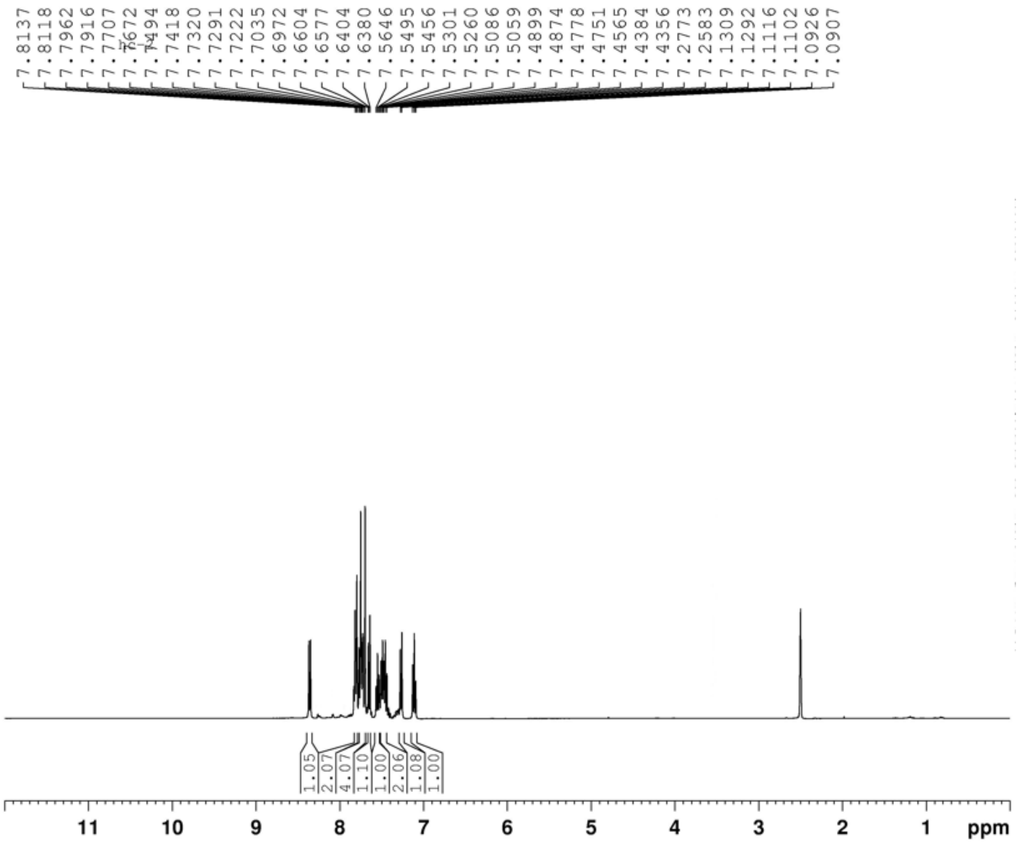

Figure S112. ^{1}H NMR spectrum of compound B3 (400 MHz, DMSO).

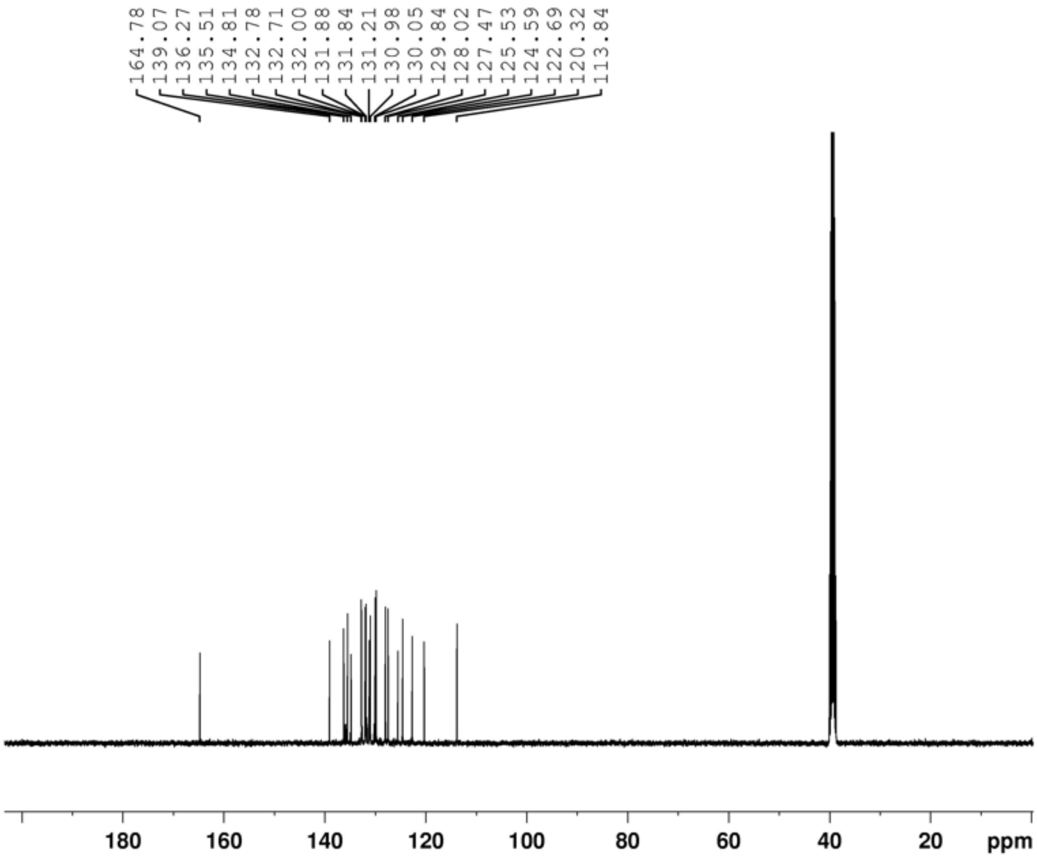

Figure S113. ^{13}C NMR spectrum of compound B3 (101 MHz, DMSO).

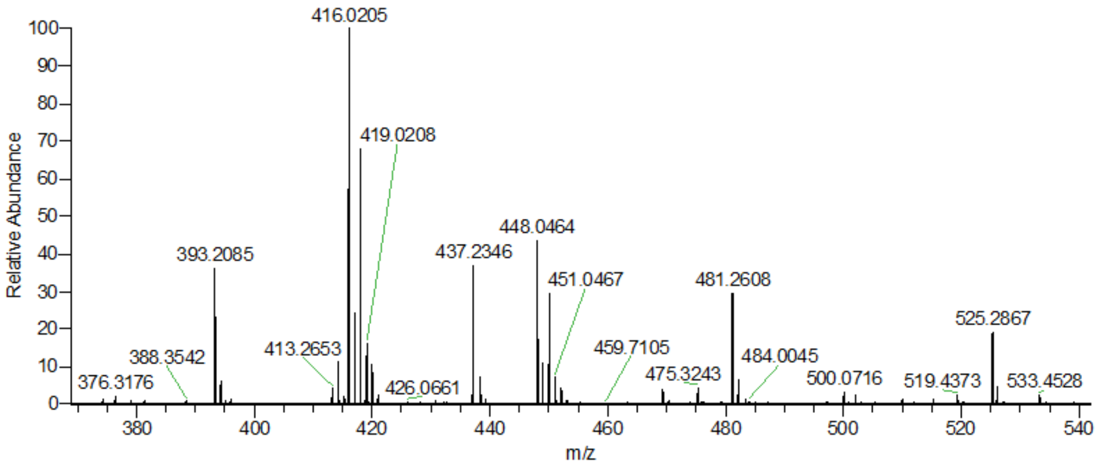

Figure S114. High resolution mass spectrum of compound B3.

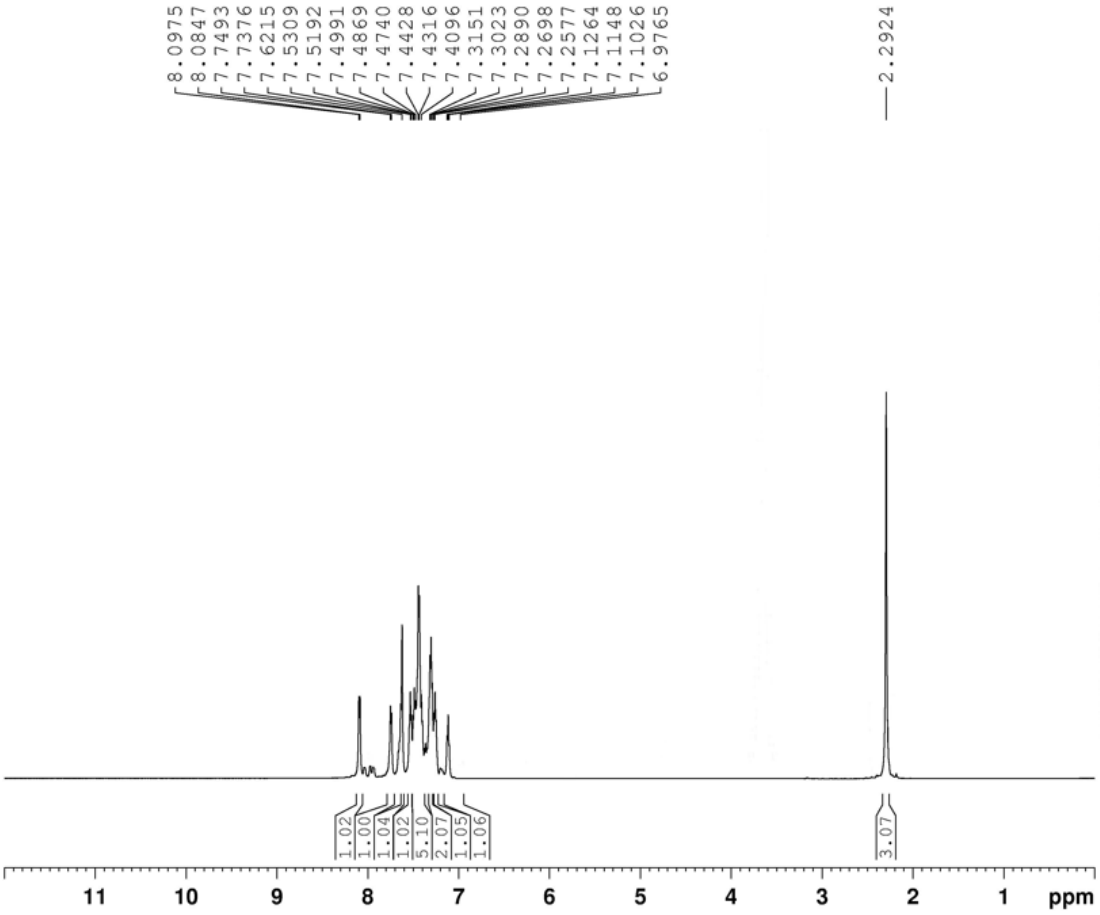

Figure S115. ^{1}H NMR spectrum of compound B4 (600 MHz, DMSO).

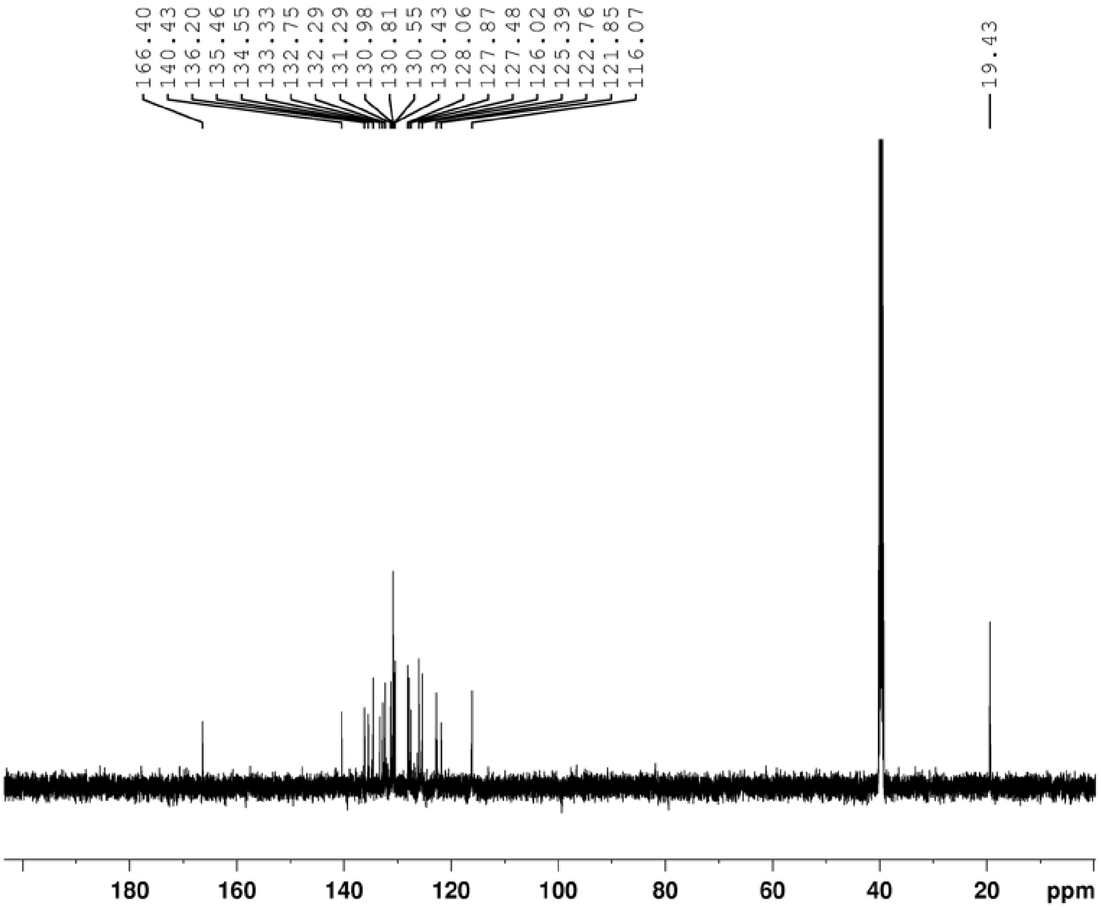

Figure S116. ^{13}C NMR spectrum of compound B4 (151 MHz, DMSO).

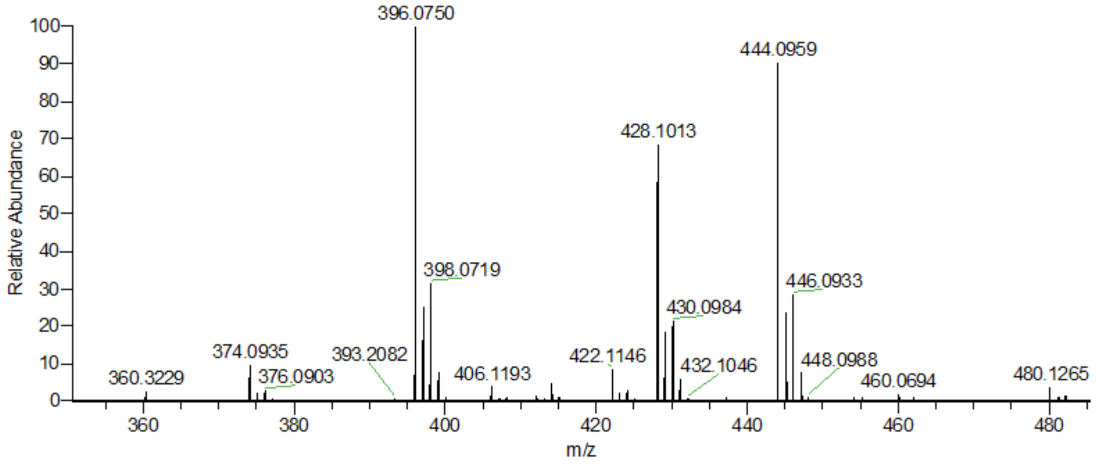

Figure S117. High resolution mass spectrum of compound B4.

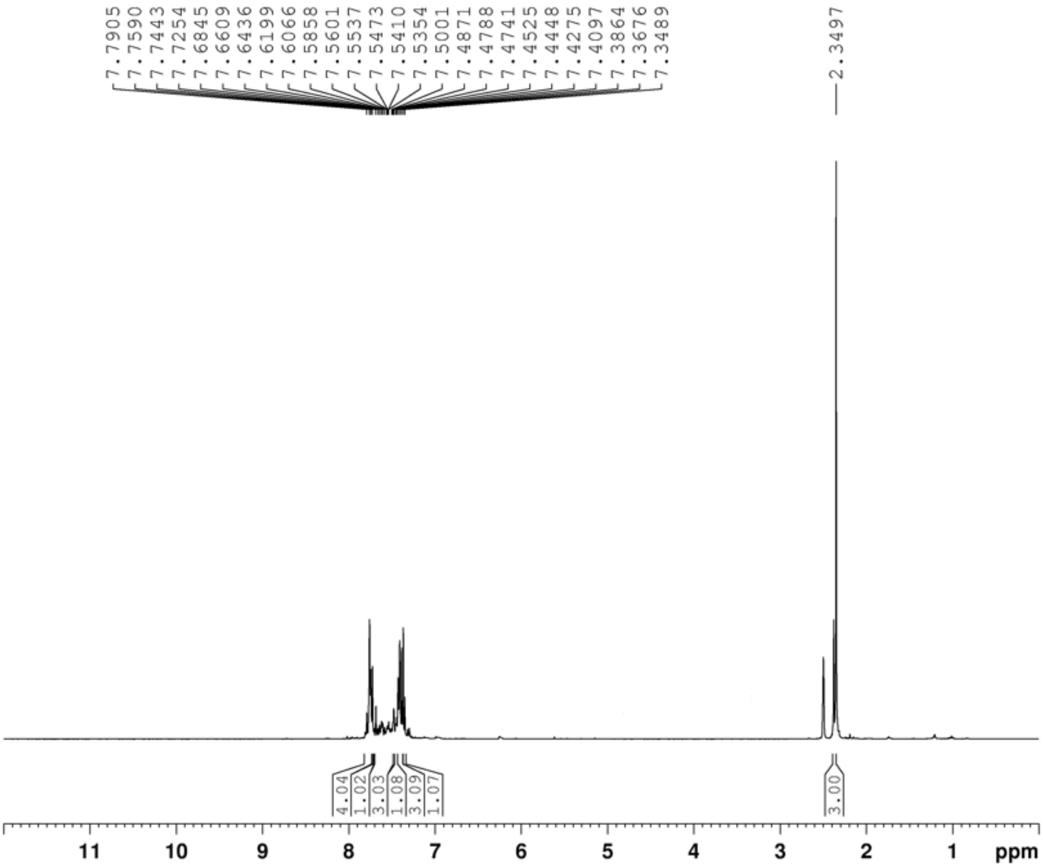

Figure S118. ^{1}H NMR spectrum of compound B5 (400 MHz, DMSO).

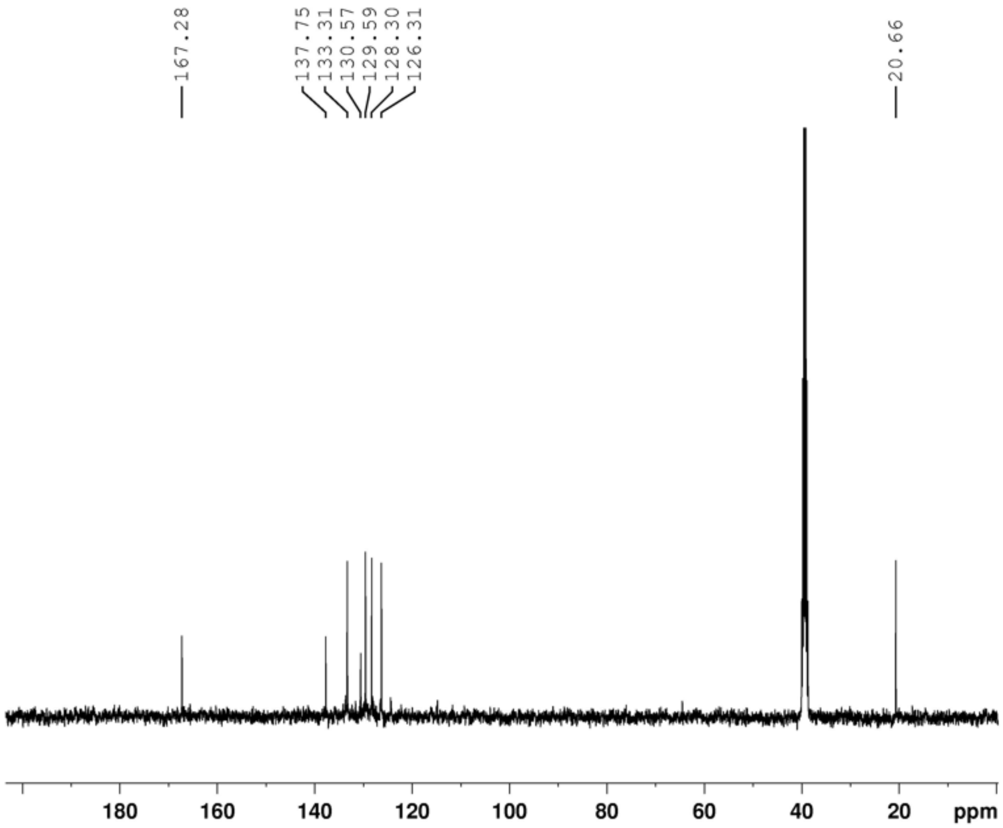

Figure S119. ^{13}C NMR spectrum of compound B5 (101 MHz, DMSO).

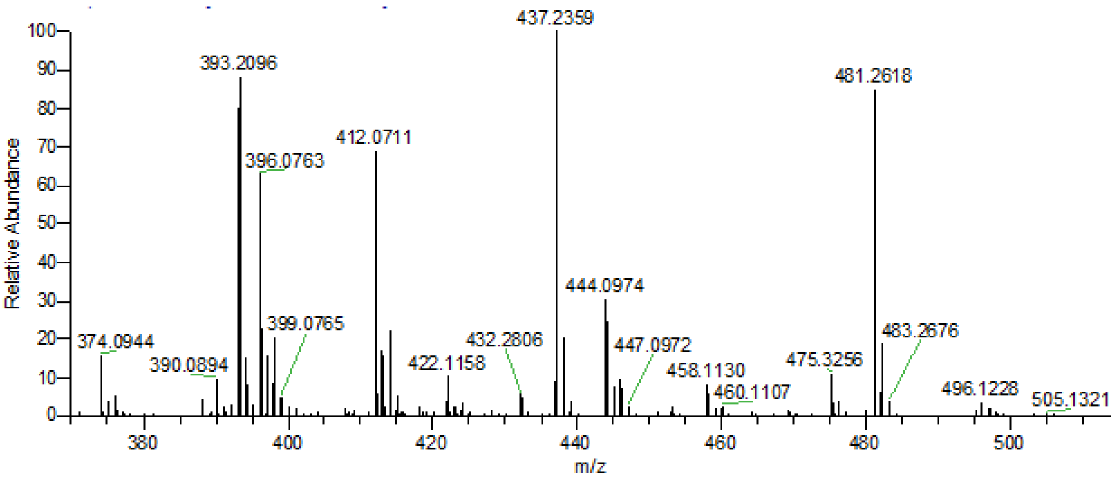

Figure S120. High resolution mass spectrum of compound B5.

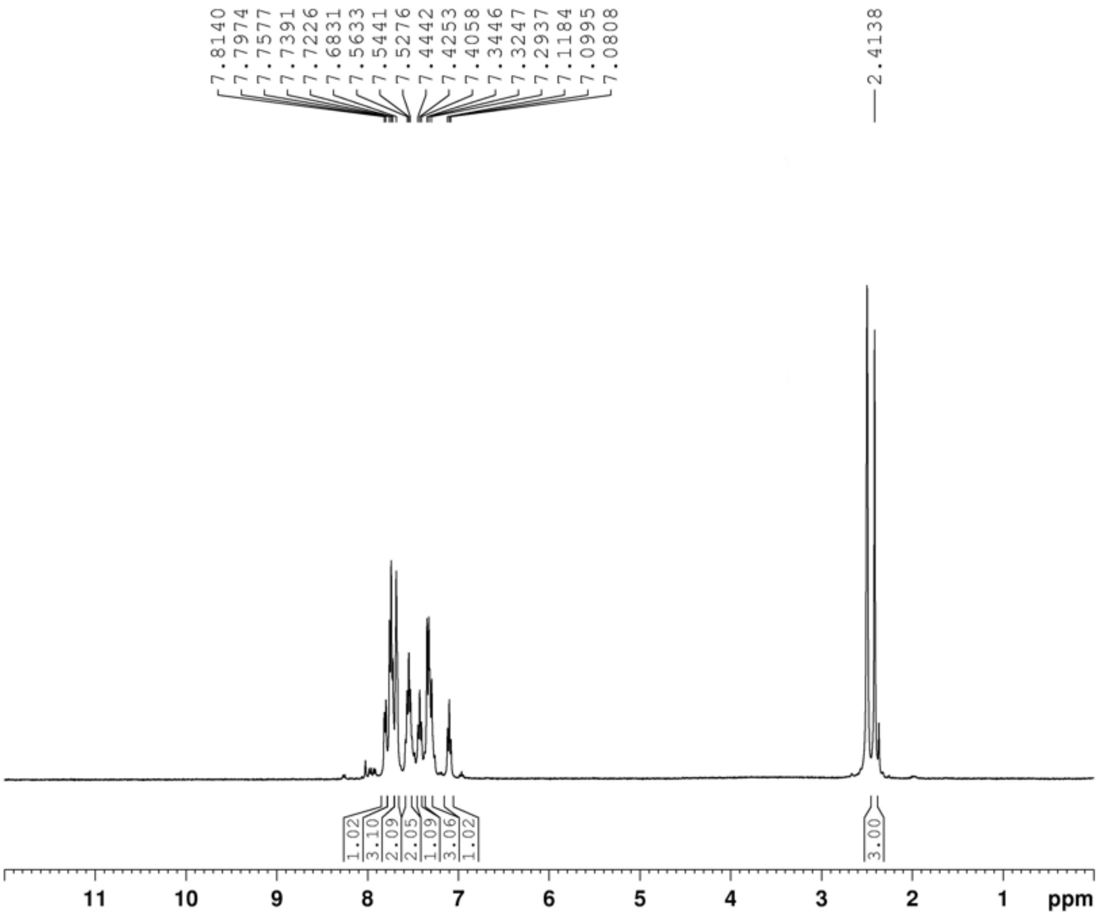

Figure S121. ^{1}H NMR spectrum of compound B6 (400 MHz, DMSO).

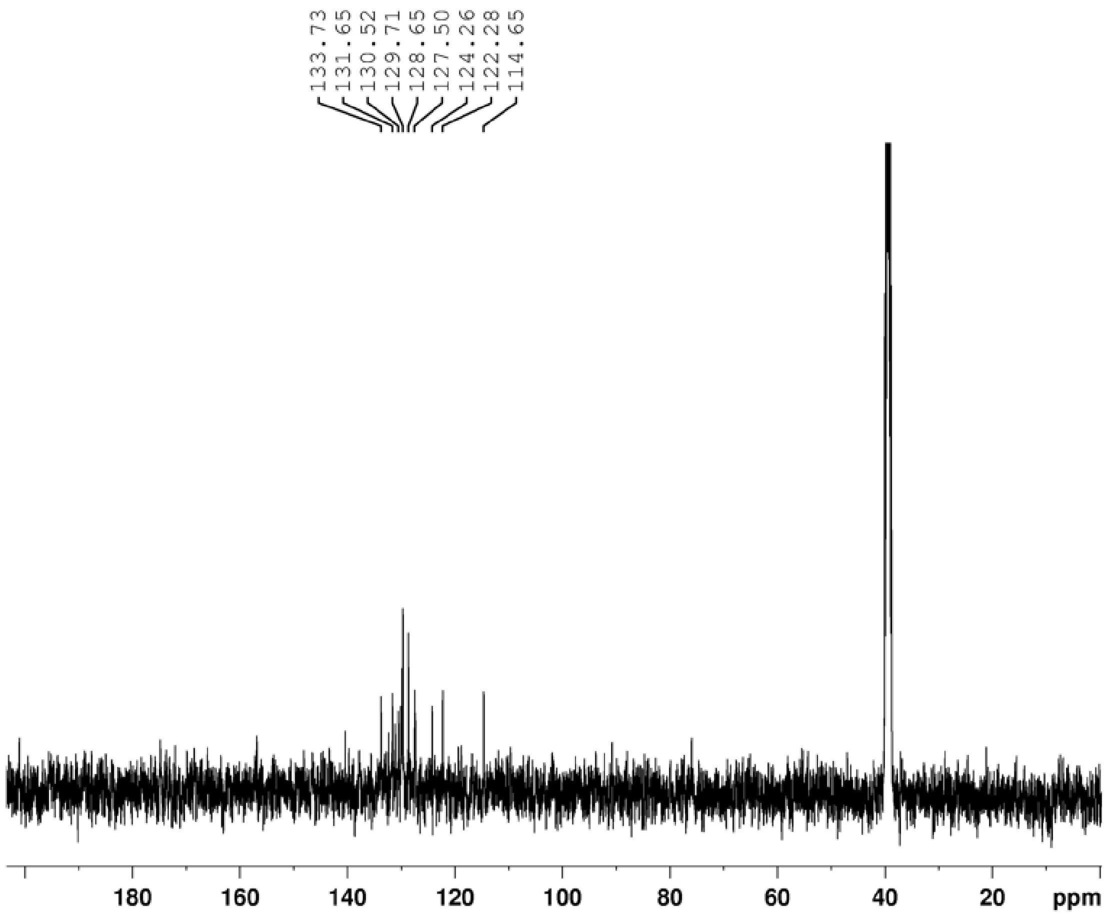

Figure S122. ^{13}C NMR spectrum of compound B6 (101 MHz, DMSO).

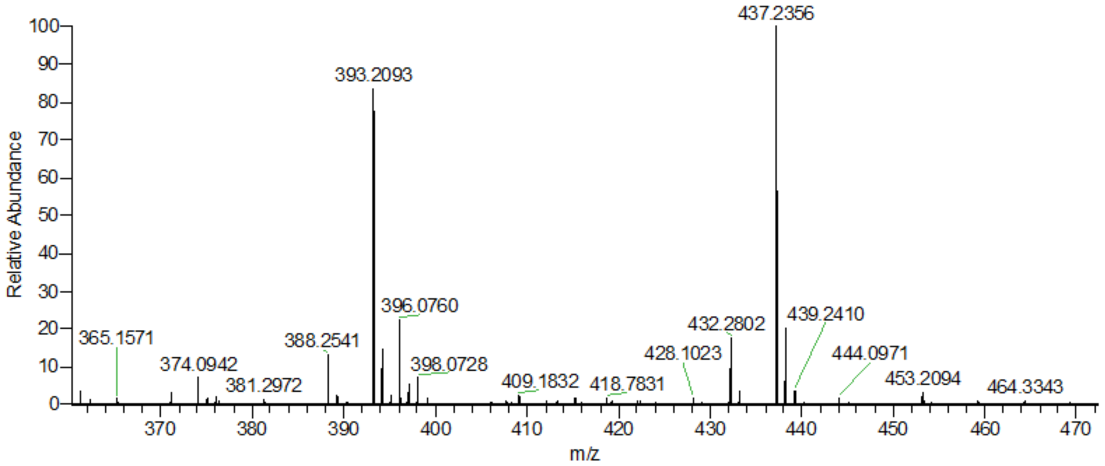

Figure S123. High resolution mass spectrum of compound B6.

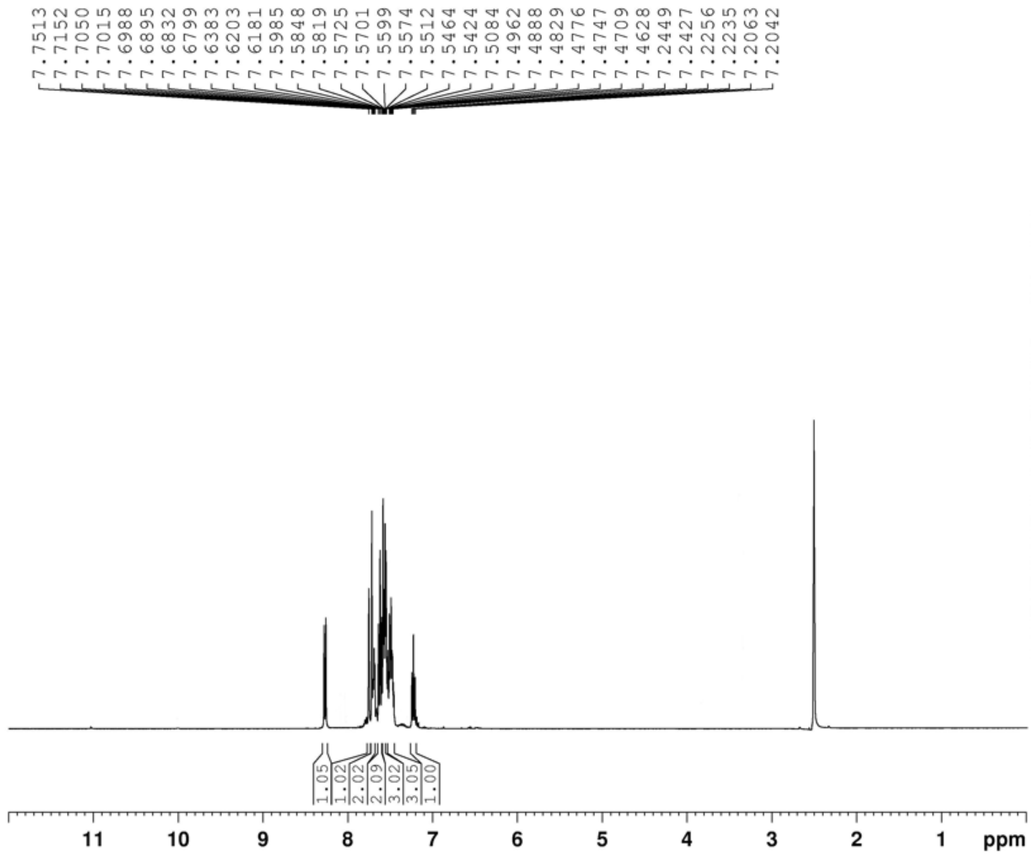

Figure S124. ^1H NMR spectrum of compound B7 (400 MHz, DMSO).

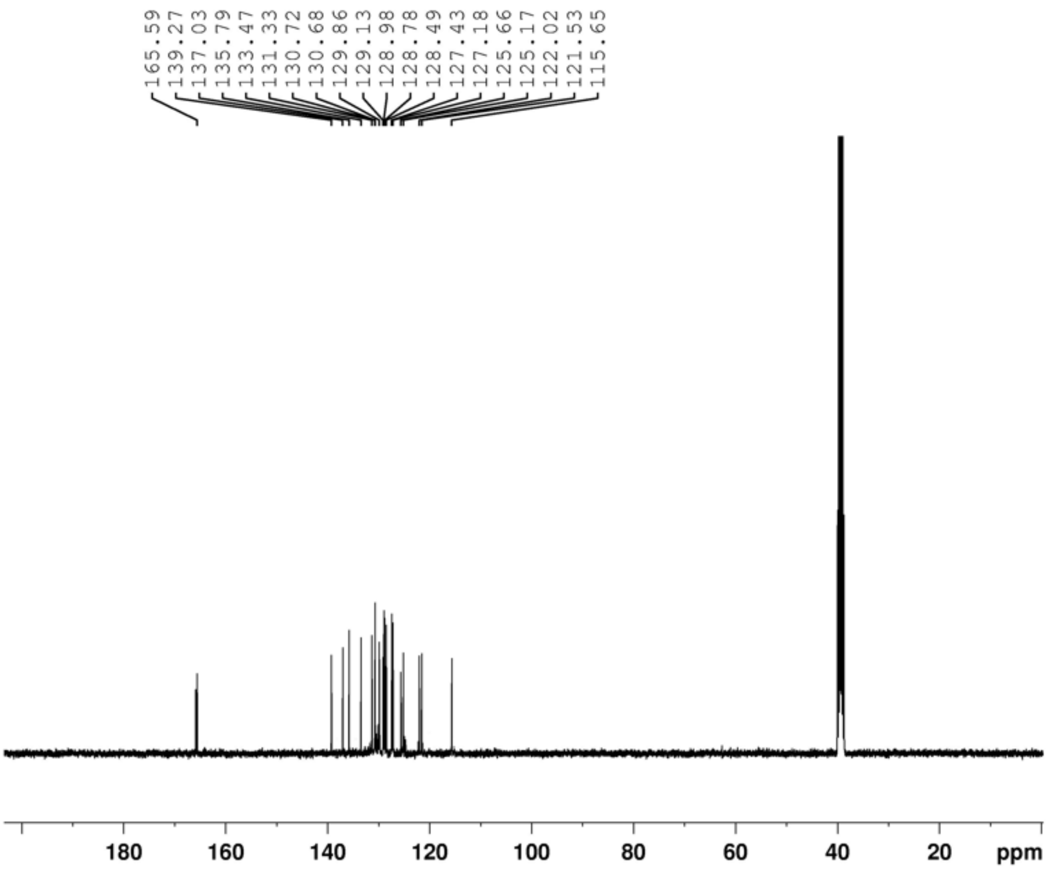

Figure S125. ^{13}C NMR spectrum of compound B7 (101 MHz, DMSO).

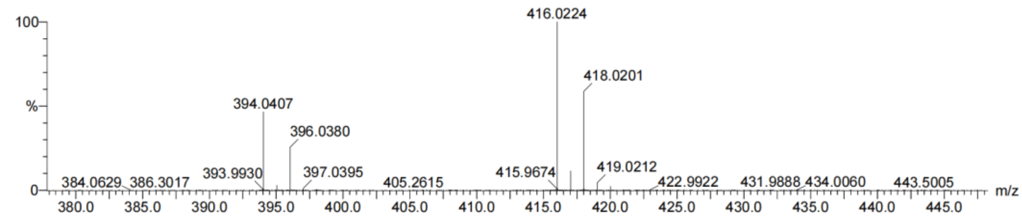

Figure S126. High resolution mass spectrum of compound B7.

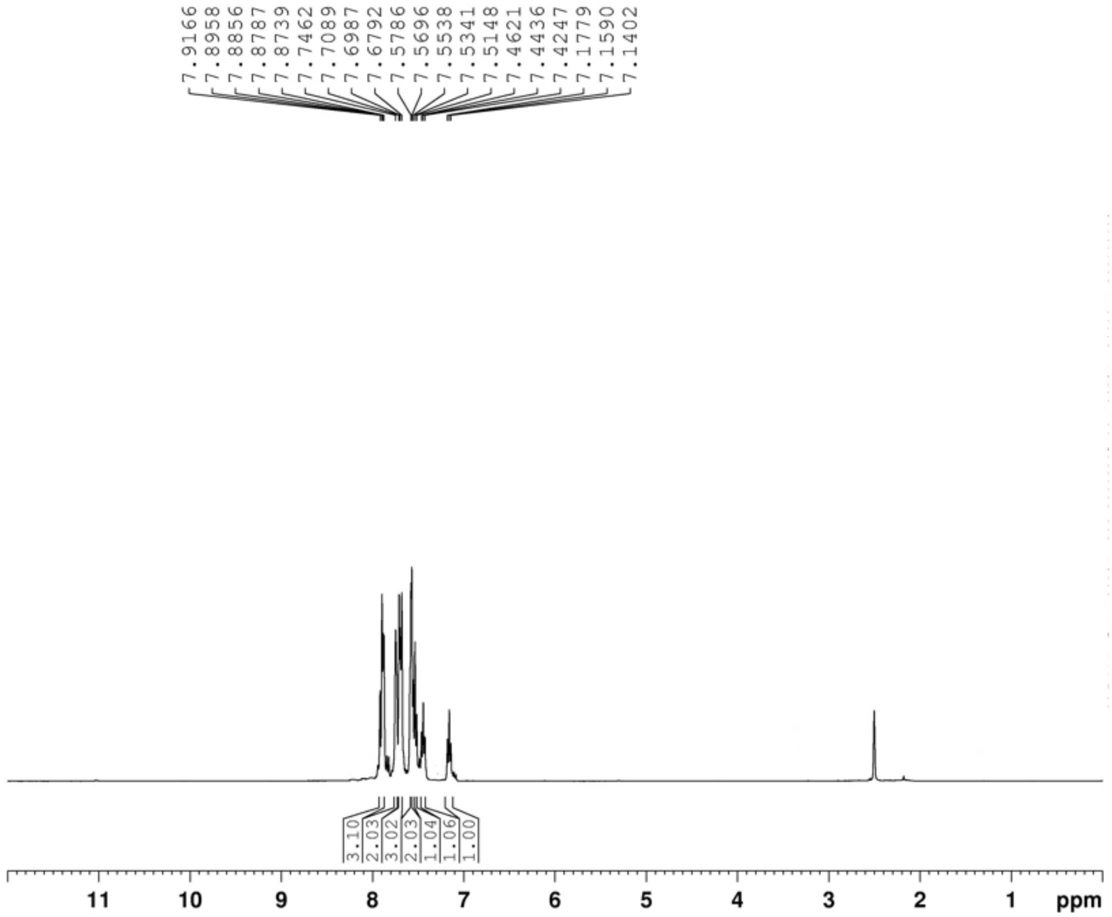

Figure S127. ^{1}H NMR spectrum of compound B8 (400 MHz, DMSO).

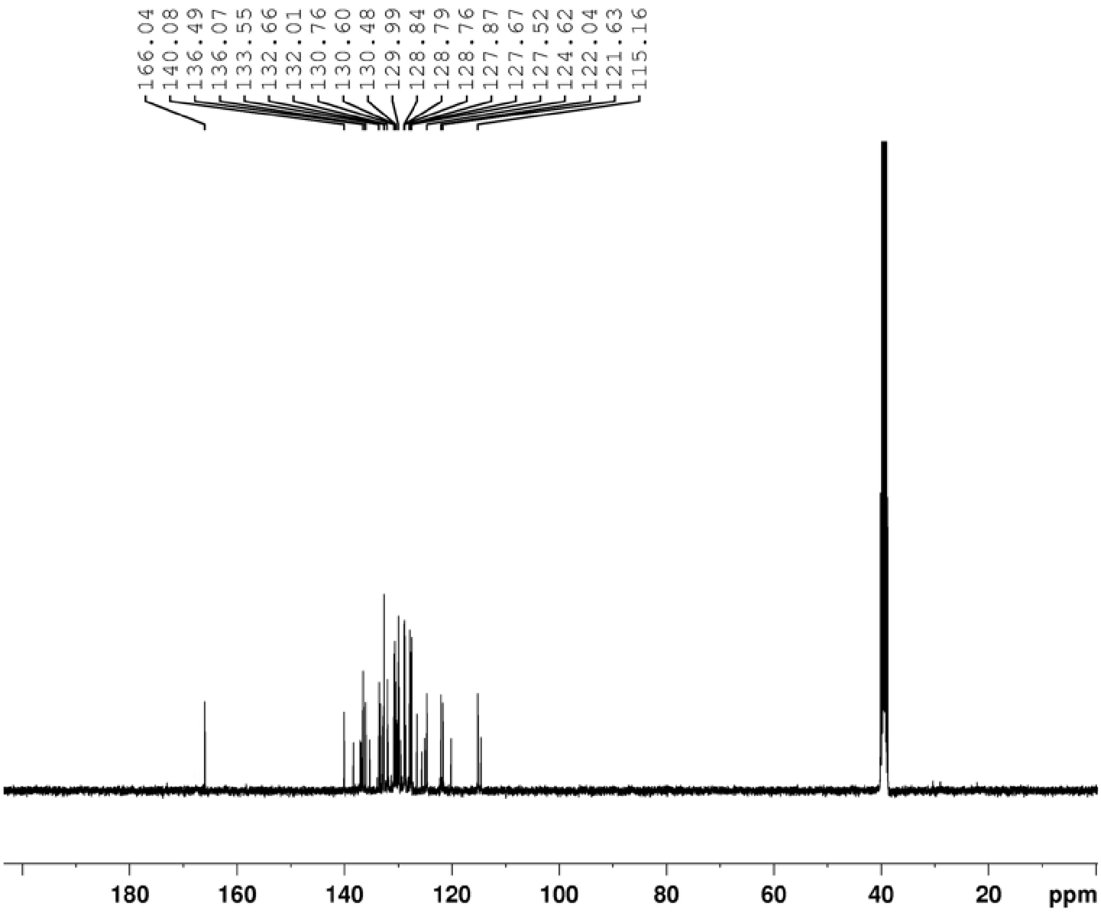

Figure S128. ^{13}C NMR spectrum of compound B8 (101 MHz, DMSO).

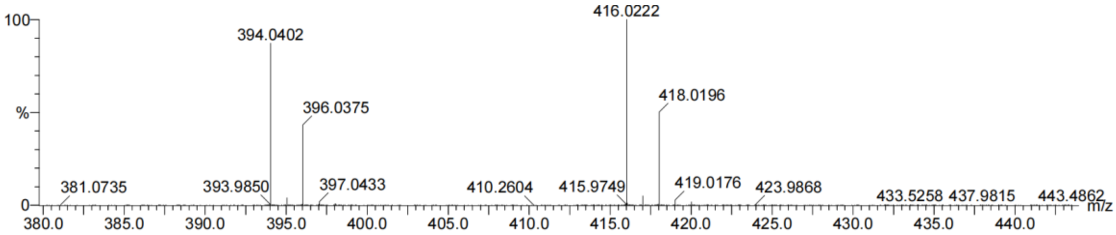

Figure S129. High resolution mass spectrum of compound B8.

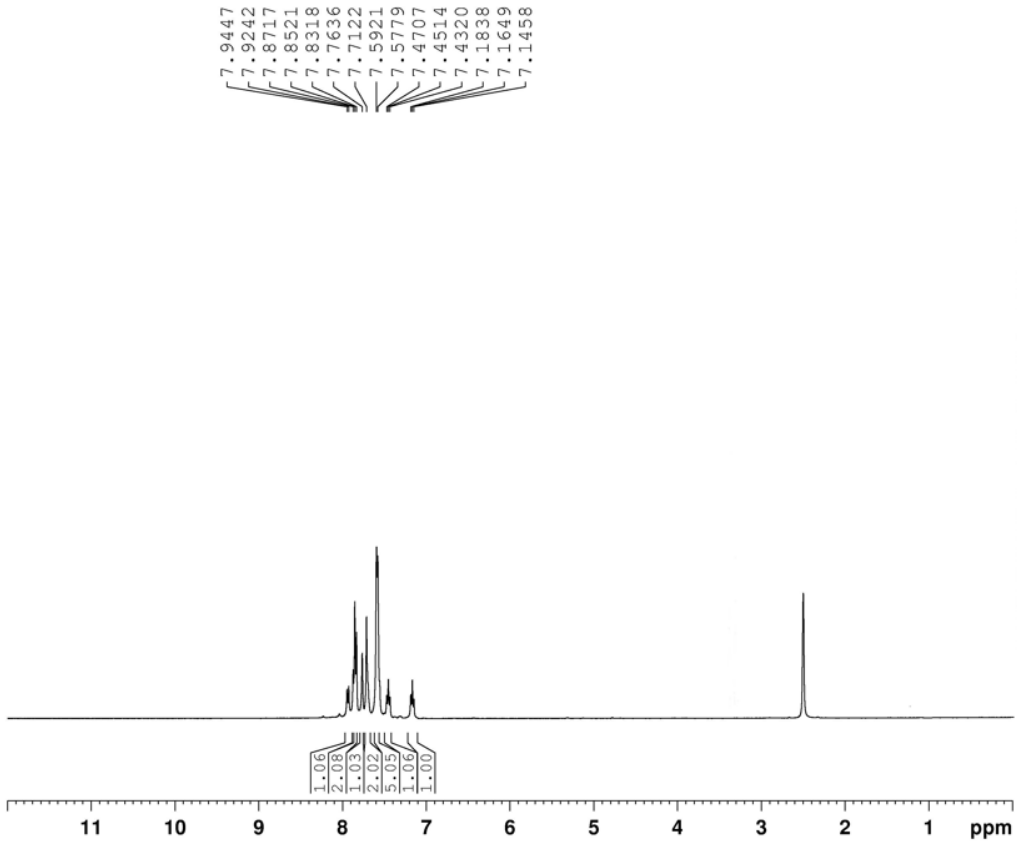

Figure S130. ^{1}H NMR spectrum of compound B9 (400 MHz, DMSO).

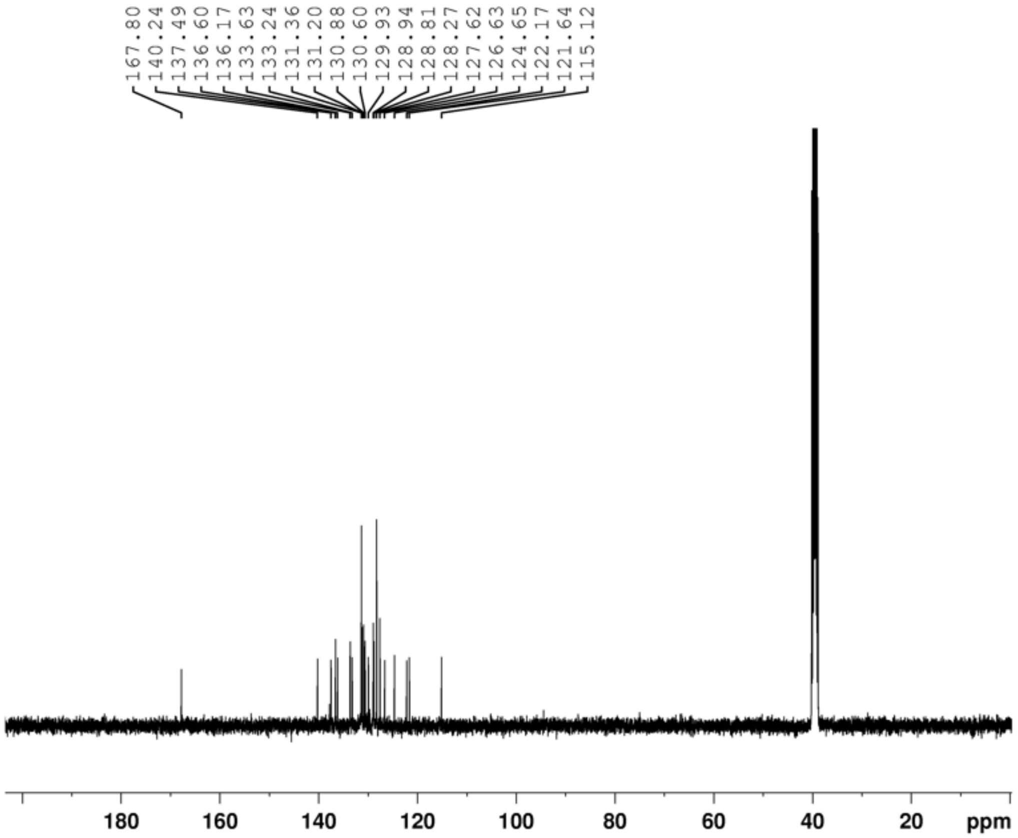

Figure S131. ^{13}C NMR spectrum of compound B9 (101 MHz, DMSO).

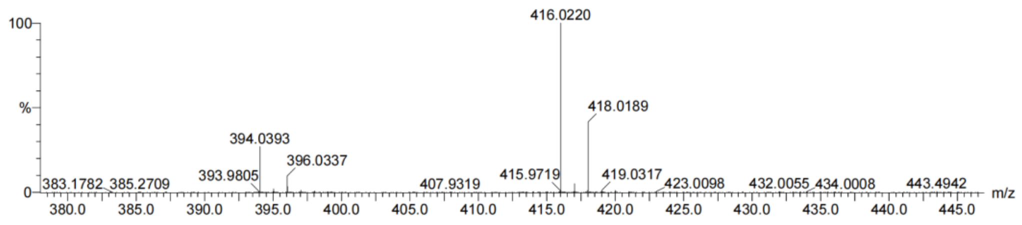

Figure S132. High resolution mass spectrum of compound B9.

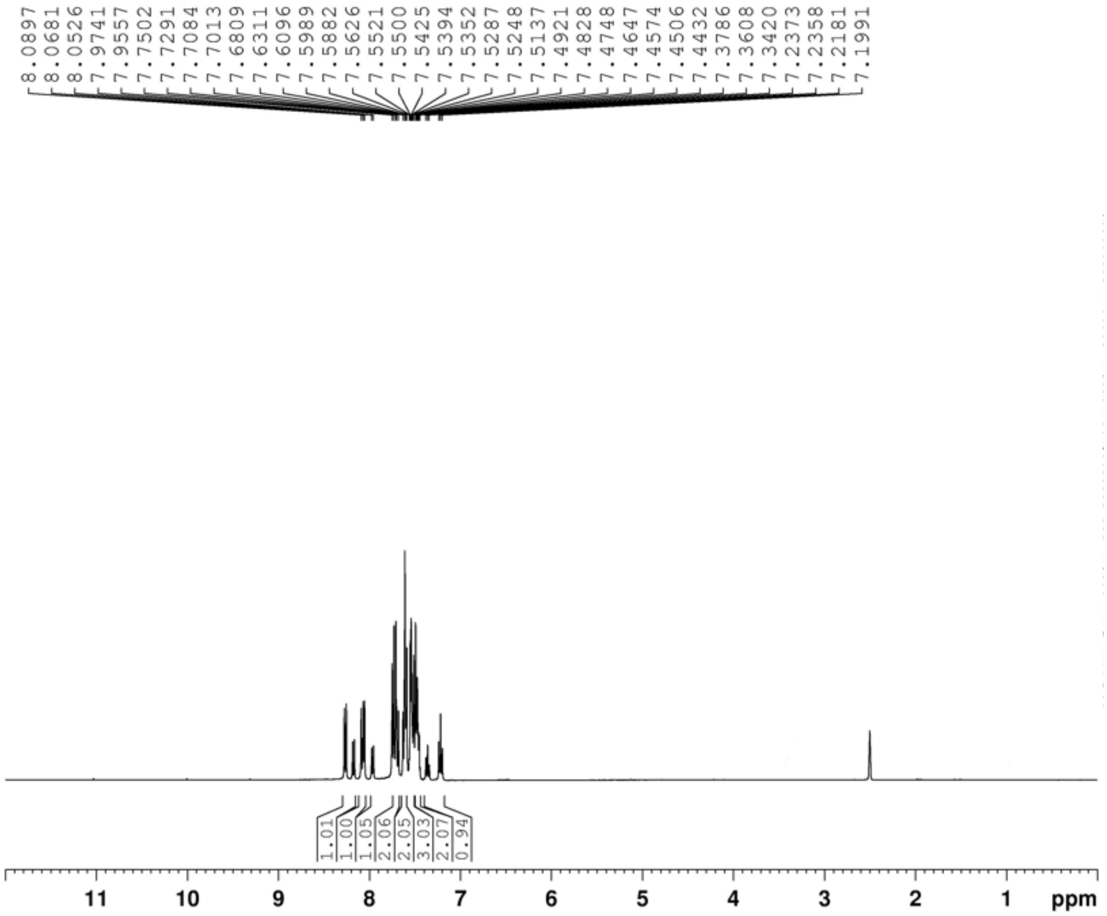

Figure S133. ^{1}H NMR spectrum of compound B10 (400 MHz, DMSO).

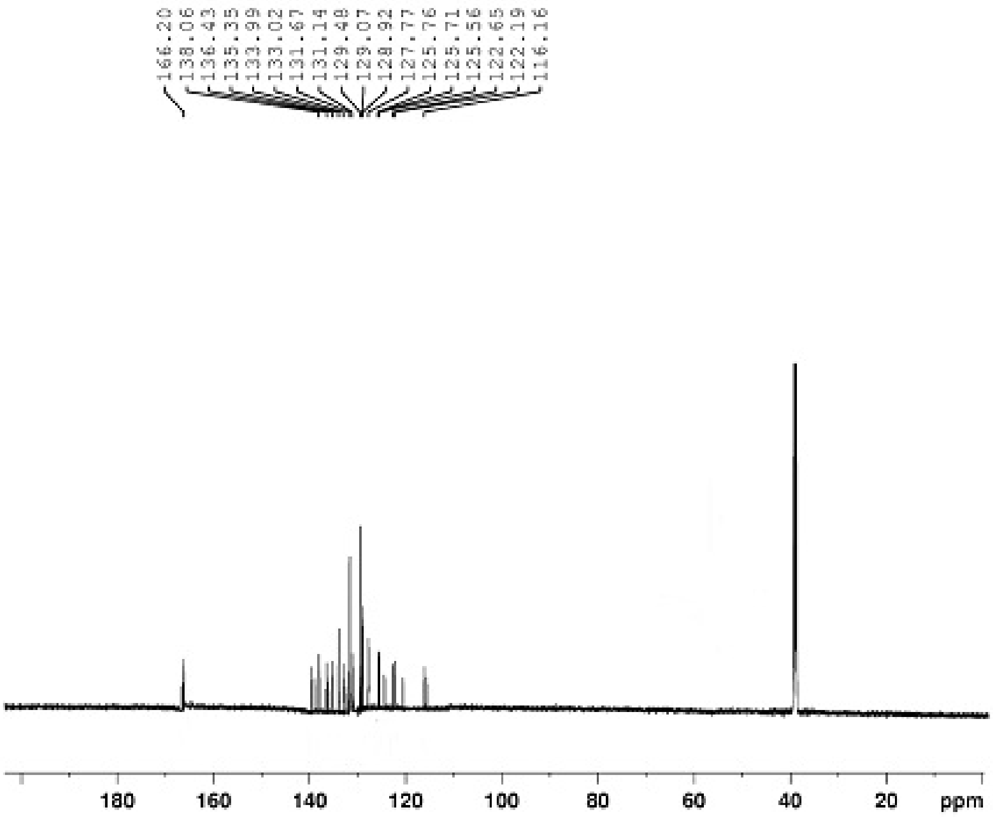

Figure S134. ^{13}C NMR spectrum of compound B10 (101 MHz, DMSO).

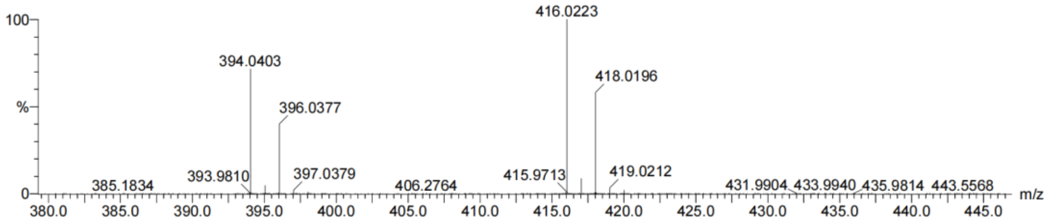

Figure S135: High resolution mass spectrum of compound B10.
